# A biomarker-stratified comparison of top-down versus accelerated step-up treatment strategies for patients with newly diagnosed Crohn's disease (PROFILE): a multicentre, open-label randomised controlled trial

**DOI:** 10.1016/S2468-1253(24)00034-7

**Published:** 2024-02-22

**Authors:** Nurulamin M Noor, James C Lee, Simon Bond, Francis Dowling, Biljana Brezina, Kamal V Patel, Tariq Ahmad, Paul J Banim, James W Berrill, Rachel Cooney, Juan De La Revilla Negro, Shanika de Silva, Shahida Din, Dharmaraj Durai, John N Gordon, Peter M Irving, Matthew Johnson, Alexandra J Kent, Klaartje B Kok, Gordon W Moran, Craig Mowat, Pritash Patel, Chris S Probert, Tim Raine, Rebecca Saich, Abigail Seward, Dan Sharpstone, Melissa A Smith, Sreedhar Subramanian, Sara S Upponi, Alan Wiles, Horace R T Williams, Gijs R van den Brink, Séverine Vermeire, Vipul Jairath, Geert R D'Haens, Eoin F McKinney, Paul A Lyons, James O Lindsay, Nicholas A Kennedy, Kenneth G C Smith, Miles Parkes, PROFILE Study Group

**Affiliations:** a Department of Gastroenterology, Cambridge University Hospitals NHS Foundation Trust, Cambridge, UK; b Cambridge Clinical Trials Unit, Cambridge University Hospitals NHS Foundation Trust, Cambridge, UK; c Department of Radiology, Cambridge University Hospitals NHS Foundation Trust, Cambridge, UK; d Department of Medicine, University of Cambridge School of Clinical Medicine, Cambridge, UK; e Genetic Mechanisms of Disease Laboratory, The Francis Crick Institute, London, UK; f Department of Gastroenterology, UCL Institute of Liver and Digestive Diseases, Royal Free Hospital, London, UK; g Medical Research Council Biostatistics Unit, University of Cambridge, Cambridge, UK; h Department of Gastroenterology, St George's University Hospitals NHS Foundation Trust, London, UK; i Department of Gastroenterology, Royal Devon University Healthcare NHS Foundation Trust, Exeter, UK; j Exeter Inflammatory Bowel Disease and Pharmacogenetics Research Group, University of Exeter, Exeter, UK; k Department of Gastroenterology, James Paget University Hospital, Great Yarmouth, UK; l Department of Gastroenterology, Royal Glamorgan Hospital, Llantrisant, UK; m GI Unit, University Hospitals Birmingham NHS Foundation Trust, Birmingham, UK; n Department of Gastroenterology, The Dudley Group NHS Foundation Trust, Dudley, UK; o Edinburgh IBD Unit, Western General Hospital, Edinburgh, UK; p Department of Gastroenterology, Cardiff and Vale University Health Board, University Hospital of Wales, Cardiff, UK; q Department of Gastroenterology, Royal Hampshire County Hospital, Winchester, UK; r Department of Gastroenterology, Guy's and St Thomas' NHS Foundation Trust, London, UK; s Gastroenterology Department, Luton and Dunstable University Hospital, Luton, UK; t Department of Gastroenterology, King's College Hospital NHS Foundation Trust, London, UK; u Department of Gastroenterology, Royal London Hospital, Barts Health NHS Trust, London, UK; v National Institute of Health Research Nottingham Biomedical Research Centre, University of Nottingham and Nottingham University Hospitals, Nottingham, UK; w Department of Gastroenterology, Ninewells Hospital, Dundee, Scotland, UK; x Department of Gastroenterology, Epsom and St Helier University Hospitals, Carshalton, UK; y Department of Gastroenterology, Liverpool University Hospitals NHS Foundation Trust, Liverpool, UK; z Department of Molecular and Clinical Cancer Medicine, Institute of Systems, Molecular and Integrative Biology, University of Liverpool, Liverpool, UK; aa Department of Gastroenterology, Basingstoke and North Hampshire Hospital, Basingstoke, UK; ab Department of Gastroenterology, West Suffolk NHS Foundation Trust, Bury St Edmunds, UK; ac Department of Gastroenterology, University Hospitals Sussex NHS Foundation Trust, Brighton, UK; ad Department of Gastroenterology, The Queen Elizabeth Hospital King's Lynn NHS Trust, King's Lynn, UK; ae Department of Gastroenterology, Imperial College Healthcare NHS Trust, St Mary's Hospital, London, UK; af Roche Innovation Center Basel, Roche, Basel, Basel-Stadt, Switzerland; ag Department of Gastroenterology and Hepatology, Department of Chronic Diseases and Metabolism, University Hospitals Leuven, KU Leuven, Leuven, Belgium; ah Division of Gastroenterology, Department of Medicine, Western University, London, Ontario, Canada; ai Department of Gastroenterology, Amsterdam University Medical Center, Amsterdam, Netherlands; aj PredictImmune Ltd, Babraham Research Campus, Cambridge, UK; ak Cambridge Institute of Therapeutic Immunology and Infectious Disease, Jeffrey Cheah Biomedical Centre, Cambridge, UK

## Abstract

**Background:**

Management strategies and clinical outcomes vary substantially in patients newly diagnosed with Crohn's disease. We evaluated the use of a putative prognostic biomarker to guide therapy by assessing outcomes in patients randomised to either top-down (ie, early combined immunosuppression with infliximab and immunomodulator) or accelerated step-up (conventional) treatment strategies.

**Methods:**

PROFILE (PRedicting Outcomes For Crohn's disease using a moLecular biomarker) was a multicentre, open-label, biomarker-stratified, randomised controlled trial that enrolled adults with newly diagnosed active Crohn's disease (Harvey-Bradshaw Index ≥7, either elevated C-reactive protein or faecal calprotectin or both, and endoscopic evidence of active inflammation). Potential participants had blood drawn to be tested for a prognostic biomarker derived from T-cell transcriptional signatures (PredictSURE-IBD assay). Following testing, patients were randomly assigned, via a secure online platform, to top-down or accelerated step-up treatment stratified by biomarker subgroup (IBDhi or IBDlo), endoscopic inflammation (mild, moderate, or severe), and extent (colonic or other). Blinding to biomarker status was maintained throughout the trial. The primary endpoint was sustained steroid-free and surgery-free remission to week 48. Remission was defined by a composite of symptoms and inflammatory markers at all visits. Flare required active symptoms (HBI ≥5) plus raised inflammatory markers (CRP >upper limit of normal or faecal calprotectin ≥200 μg/g, or both), while remission was the converse—ie, quiescent symptoms (HBI <5) or resolved inflammatory markers (both CRP ≤ the upper limit of normal and calprotectin <200 μg/g) or both. Analyses were done in the full analysis (intention-to-treat) population. The trial has completed and is registered (ISRCTN11808228).

**Findings:**

Between Dec 29, 2017, and Jan 5, 2022, 386 patients (mean age 33·6 years [SD 13·2]; 179 [46%] female, 207 [54%] male) were randomised: 193 to the top-down group and 193 to the accelerated step-up group. Median time from diagnosis to trial enrolment was 12 days (range 0–191). Primary outcome data were available for 379 participants (189 in the top-down group; 190 in the accelerated step-up group). There was no biomarker–treatment interaction effect (absolute difference 1 percentage points, 95% CI –15 to 15; p=0·944). Sustained steroid-free and surgery-free remission was significantly more frequent in the top-down group than in the accelerated step-up group (149 [79%] of 189 patients *vs* 29 [15%] of 190 patients, absolute difference 64 percentage points, 95% CI 57 to 72; p<0·0001). There were fewer adverse events (including disease flares) and serious adverse events in the top-down group than in the accelerated step-up group (adverse events: 168 *vs* 315; serious adverse events: 15 *vs* 42), with fewer complications requiring abdominal surgery (one *vs* ten) and no difference in serious infections (three *vs* eight).

**Interpretation:**

Top-down treatment with combination infliximab plus immunomodulator achieved substantially better outcomes at 1 year than accelerated step-up treatment. The biomarker did not show clinical utility. Top-down treatment should be considered standard of care for patients with newly diagnosed active Crohn's disease.

**Funding:**

Wellcome and PredictImmune Ltd.

**Research in context**

**Evidence before this study**

We searched PubMed for articles in any language published up to Sept 17, 2023, with the search terms “Crohn's disease”, “early disease”, “combined immunosuppression”, “treatment algorithm”, and “biomarker-stratified”. This produced no results. Dropping the requirement for “biomarker-stratified” yielded abstracts for three high quality interventional clinical trials. The Step-Up/Top-Down trial recruited 133 immunosuppressant and anti-TNF-naive patients with moderate to severely active Crohn's disease within 4 years of diagnosis. Patients were randomised to early combined anti-TNF and immunosuppressant induction followed by intermittent anti-TNF treatment to control disease flares, or conventional step-up therapy. At week 52, 61% and 42%, respectively, were in corticosteroid-free symptomatic remission (p=0·028), with no differences in serious adverse events between groups. The CALM trial randomised 244 adults with Crohn's disease (mean duration 1 year) and endoscopic activity to tight control (treatment escalation for elevated CRP or calprotectin as well as symptoms) versus conventional management (treatment escalation for symptomatic flares). More patients in the tight control group achieved the primary endpoint of mucosal healing at week 48 (46% *vs* 30%), with no difference in adverse events**.** Although REACT was framed as testing early combined immunosuppression with a TNF antagonist and antimetabolite, its participants had an average disease duration of over 12 years. The primary endpoint of steroid-free remission (using a symptom-based score alone without corroboration by objective evidence of inflammation) at 12 months, was similar between the 921 patients treated with early combined immunosuppression and the 806 treated conventionally (66% *vs* 62%; p=0·52). However, the composite secondary outcome of serious adverse events (complications, hospitalisation, surgery) was lower at 24 months in the early combined immunosuppression group. Although previous trials support earlier use of anti-TNF therapy, typically in combination with an immunomodulator, an accelerated step-up approach to the management of Crohn's disease, where treatment is escalated in intensity until the tendency to relapse is controlled, remains the most common strategy in the UK and globally. Under this strategy, the course of Crohn's disease, including the frequency of disease flares, varies substantially between patients and is unpredictable. To better understand the basis of these heterogeneous outcomes and enable personalised therapy in IBD a blood-based biomarker derived from transcriptional signatures in T-cell subsets was developed and shown in observational datasets to be associated with need for future treatment escalation. Testing its utility, however, would require a biomarker-stratified randomised controlled trial.

**Added value of this study**

Patients with newly diagnosed active Crohn's disease were recruited to a biomarker-stratified interventional trial based on a pragmatic clinical trial design. The biomarker did not demonstrate clinical utility to guide the treatment strategy for patients living with Crohn's disease. However, top-down treatment with combination infliximab and immunomodulator was significantly better than accelerated step-up (conventional) treatment in maintaining steroid-free and surgery-free remission throughout 48 weeks of follow-up. Top-down treatment also showed greater efficacy in achieving endoscopic remission, improved quality of life, and reduced number of flares requiring treatment escalation. Top-down treatment was safer than accelerated step-up treatment, with fewer adverse and serious adverse events, no increased rate of infection, and reduced need for urgent abdominal surgery.

**Implications of all the available evidence**

Top-down treatment with combination infliximab and immunomodulator should be adopted as the standard of care for most patients with newly diagnosed active Crohn's disease.

## Introduction

Crohn's disease is a chronic, relapsing form of inflammatory bowel disease (IBD) characterised by recurrent flares that can lead to progressive bowel damage.^1^ Conventional management involves treatment of acute flares with corticosteroids and, where required, addition of immunomodulators and advanced therapies to achieve sustained remission. However, the disease course and clinical outcomes for a newly diagnosed patient are highly uncertain. The frequency and severity of flares, likelihood of response to any particular treatment, and rate of progression to complications requiring surgery, including the intestinal strictures and fistulae that characterise Crohn's disease, are all unpredictable.^2^ The advent of biologic treatments, including anti-TNF therapy, has helped mitigate adverse outcomes but surgical resection is still required in 17–25% of patients within 5 years of diagnosis.^3^, ^4^

Ideally, a safe and affordable treatment strategy would be available that was effective in all patients from diagnosis. Thiopurines appear ineffective in this context and the efficacy of advanced therapies from diagnosis remains largely unstudied.^5^, ^6^ Indeed, Crohn's disease trials usually enrol patients years after diagnosis and typically show benefit of active comparator over placebo of only 10–20%.^7^ However, one consistent observation based on both post-hoc clinical trial analyses and retrospective cohorts is that the earlier patients receive advanced treatments the more effective they are.^8^, ^9^ Notably, D'Haens and colleagues^10^ randomised 133 patients within 4 years of Crohn's diagnosis to either infliximab induction followed by azathioprine or conventional step-up care. Remission at week 52 was significantly higher in patients receiving early combined immunosuppression. Similarly, the CALM and REACT trials, which enrolled patients with established Crohn's disease in flare, found that earlier escalation to anti-TNF therapy resulted in fewer major adverse outcomes than treatment with conventional step-up strategies.^11^, ^12^

These studies, combined with the advent of substantially cheaper biosimilars, have resulted in more widespread and earlier use of anti-TNF therapies in the management of Crohn's disease. Nevertheless, most patients are not prescribed biologics from diagnosis, but only when conservative management fails or other therapies, including immunomodulators, prove ineffective—a strategy supported by several international guidelines.^13^, ^14^, ^15^ The ability to predict which patients would benefit from early advanced therapies would enable targeting of these treatments to patients who truly need them while minimising their use in others.^16^ Several biomarkers have been proposed in Crohn's disease. These include a 17-gene blood-based prognostic biomarker previously shown to categorise patients into two approximately equal groups termed IBDhi and IBDlo. These groups were associated with higher or lower risk of requiring future treatment escalation but not clinical, biochemical, or imaging markers of severity at the time of blood draw.^17^ To date neither this nor any other biomarker has been formally tested in IBD.

Here we report the results of PROFILE (PRedicting Outcomes For Crohn's disease using a moLecular biomarker), a randomised controlled trial designed to test the clinical utility of the 17-gene blood-based biomarker in guiding therapy. The hypothesis was that patients in the biomarker-defined IBDhi group would be at higher risk of recurrent flares and hence gain more benefit from early advanced therapy than those in the IBDlo group. Patients newly diagnosed with Crohn's disease were therefore randomised to either top-down (infliximab plus immunomodulator) or accelerated step-up (conventional) therapy, stratified by the biomarker.

## Methods

### Study design

**Figure 1:**
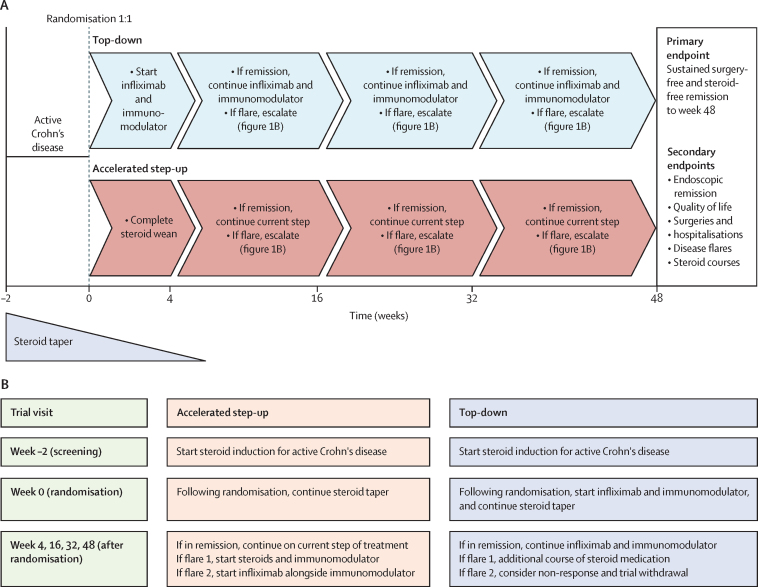
Trial design (A) Trial design. (B) Trial visits and escalation summary.

PROFILE was a randomised, open-label, active-controlled, biomarker-stratified trial. Patients were randomised to either top-down or accelerated step-up treatment strategies (figure 1). Cambridge South research ethics committee approved the protocol (number 17/EE/0382),^18^ and the trial followed Good Clinical Practice guidelines.

### Participants

Patients aged 16–80 years with newly diagnosed active Crohn's disease were enrolled. All patients gave written informed consent. Data on biological sex (male/female) was collected based on self-report by patients. Inclusion required all of: (1) Crohn's disease diagnosed within 6 months using standard clinical, endoscopic, histological, and radiological methods; (2) active, symptomatic disease (corresponding to Harvey-Bradshaw Index [HBI] ≥7); (3) biochemical evidence of active inflammation with either serum C-reactive protein (CRP) above the upper limit of normal (ULN), faecal calprotectin of 200 μg/g or more, or both; (4) endoscopic evidence of active Crohn's disease (Simple Endoscopic Score for Crohn's disease [SES-CD] ≥4 for ileal-only disease or ≥6 for ileocolonic/colonic disease); and (5) naive to immunomodulator and biologic therapy. Patients with clinically significant obstructive or peri-anal disease were excluded (appendix 1 pp 5–6).

At the screening visit Crohn's disease activity was assessed using the HBI, concurrent blood test results, and findings of ileo-colonoscopy performed within 6 months. Potential participants underwent blood draw, including for the biomarker assay,^17^ and stool sampling for faecal calprotectin. All patients were prescribed an 8-week course of oral steroids with prednisolone or budesonide (local investigator decision) for active Crohn's disease.

At the baseline visit, 2 weeks later, all results were reviewed, excluding biomarker results, which remained blinded to patients and investigators. Patients meeting eligibility criteria were randomised to either combination intravenous infliximab plus immunomodulator (top-down therapy) or protocolised accelerated step-up therapy. Local investigators decided which immunomodulator—azathioprine, low-dose mercaptopurine with allopurinol, or methotrexate. Although sites could undertake thiopurine drug monitoring, levels-based dose optimisation was not protocolised. The protocol did specify that patients could have an accelerated steroid wean after commencing infliximab but could not have dose intensification of infliximab.

### Randomisation and masking

Eligible participants attended a baseline visit and were randomised 1:1 to top-down or accelerated step-up treatment. Stratified block randomisation was used, stratifying on biomarker subgroup (IBDhi *vs* IBDlo), disease location (colon only *vs* other), and mucosal inflammation (mild *vs* moderate *vs* severe), with a randomly generated block size (block size four or six). Mucosal inflammation was judged as mild, moderate, or severe by local investigators based on subjective, clinical assessment of endoscopic appearances. Block size was maintained within the strata, which did not include sites. Sealed Envelope version 17.2.1 was used for randomisation and patient allocation. This secure, online software allowed local site investigators to register individuals, work through eligibility criteria for PROFILE, and then randomise patients. Blinding to biomarker status was maintained throughout.

### Procedures

For participants starting on an immunomodulator, the choice was at the local investigator's discretion, between azathioprine, low-dose mercaptopurine with allopurinol, or methotrexate (for dosing details see appendix 2).

For participants starting on infliximab, a 5 mg/kg dose was used with standard induction at 0, 2, and 6 weeks followed by maintenance infusions every 8 weeks (appendix 2). Patients with non-response after induction had early treatment withdrawal and reverted back to standard care with their local clinical team.

Participants were reviewed at weeks 4, 16, 32, and 48 post-baseline or at ad-hoc visits if unwell between scheduled visits (figure 1). HBI and any adverse events were recorded, with blood and stool samples sent at each visit. Flares were defined by symptoms of active Crohn's disease (HBI ≥5) with biochemical evidence of active inflammation (CRP >ULN or faecal calprotectin ≥200 μg/g, or both). The protocol mandated a course of steroids to treat such flares. Participants in the accelerated step-up group were also prescribed an immunomodulator with this course of steroids, and if a further flare occurred, infliximab was commenced alongside the immunomodulator. Those not meeting flare criteria continued current management without therapy escalation. The end-of-trial visit occurred 48 weeks after randomisation.

Ileo-colonoscopy to assess disease activity using SES-CD was performed and where possible video-recorded by local site investigators before randomisation and 48 weeks after randomisation. The 17-gene biomarker test to determine IBDhi or IBDlo subgroup assignment was performed using the PredictSURE-IBD assay (PredictImmune Ltd; appendix 1 p 3).

### Outcomes

The primary endpoint was the incidence of sustained surgery-free and steroid-free remission from completion of the protocolised (maximum 8-week) steroid induction course through to week 48. The definitions of flare and remission states were each the composite of symptoms and inflammatory markers. Flare required active symptoms plus raised inflammatory markers. Remission was the converse—ie, symptoms had resolved (HBI <5) or inflammatory markers had settled (both CRP ≤ULN and calprotectin <200 μg/g) or both at all trial visits after baseline (appendix 1 p 7). The definition of remission in the primary endpoint being a composite of both clinical and biochemical remission (as opposed to symptoms only) was clarified in the statistical analysis plan before database lock and before any analysis being undertaken (appendix 2).

The hierarchical secondary endpoints were: (1) endoscopic remission at week 48 defined by absence of ulceration (including aphthous ulceration)—ie, SES-CD ulcer subscore of 0—based on centrally read endoscopic scores,^19^ or where ileo-colonoscopies had not been video-recorded, locally read SES-CD scores; (2) quality of life assessment averaged across weeks 16, 32, and 48 using the IBD-Q score; (3) number of flares requiring treatment escalation by week 48; (4) cumulative steroid exposure by week 48; and (5) the number of Crohn's-related hospital admissions and surgeries by week 48.

Tertiary endpoints were unranked and included assessments of time to event from baseline to first and to second flare or surgery; CRP response at weeks 4, 16, 32, and 48 (comparison of median CRP in each group); calprotectin response at weeks 16, 32, and 48 (comparison of median calprotectin in each group); and week 48 biochemical remission (CRP ≤ULN and calprotectin <200 μg/g), endoscopic response (defined as SES-CD decrease ≥50% from baseline), and deep endoscopic remission (total SES-CD of 0). Additional tertiary endpoints are listed in appendix 1 (pp 8–9).

### Statistical analysis

The primary estimand was the interaction between treatment and biomarker for the primary endpoint. The sample size was calculated based on estimated remission rates through to week 48 for IBDhi of 0·3 with accelerated step-up and 0·7 with top-down treatment; and for IBDlo of 0·8 with accelerated step-up and 0·9 with top-down treatment. A sample size of 333 was needed to achieve 92% power at a two-sided 5% significance level. To allow for a 17% withdrawal rate, we aimed to enrol 400 participants.

The primary analysis of PROFILE focused on the biomarker–treatment interaction. Key and complementary analyses prespecified in the statistical analysis plan were to compare treatment and safety effects between the accelerated step-up and top-down therapy groups. All primary, secondary, and tertiary endpoint analyses used the full analysis population defined as all participants who met PROFILE eligibility criteria and were randomised (equivalent to an intention-to-treat analysis). Patients receiving dose-intensified infliximab or who had to stop infliximab or immunomodulators due to intolerance were included in the full trial population primary analysis but excluded from the per-protocol analysis.

The safety population additionally required receipt of some trial treatment; in reality this matched the full analysis population. Additional prespecified analyses were performed using a modified per-protocol treatment population, defined as all participants not substantially deviating from the treatment protocol. This was determined by an expert adjudication committee who also assessed the total number of steroid courses and treatment escalations for each participant. Time was accounted for using orthogonal quadratic polynomials in the longitudinal regression model, and we report the main effect comparisons of treatment and biomarker.

Missing values were assumed missing at random. Regression analyses that adjusted for covariates or accounted for within-participant correlation in repeat-measures analysis were performed using multiple imputation to account for missing baseline values. If their proportion fell below 5% then no further sensitivity analyses were undertaken. For all endpoints, the main focus was on estimating the interaction between treatment and biomarker, defined as a difference in differences. The treatment effect within each subgroup was also estimated.

To formally control for multiple testing, a closed testing procedure was performed over the primary and five secondary analyses, testing the biomarker–treatment interaction and restricting the family-wise type I error rate to an overall 5% significance level. The analyses estimated for the main effect of the biomarker and treatment, and the interaction between the biomarker and treatment, adjusting for baseline variables. These were endoscopic mucosal inflammation (mild, moderate, or severe); disease location (ileal, colonic, or ileocolonic); disease behaviour (inflammatory or other); smoking status (never, former, or current); age (16–39, 40–64, or ≥65 years); BMI (0–19·9, 20·0–24·9, 25·0–29·9, or ≥30 kg/m^2^); CRP (continuous); calprotectin (continuous); course of steroids prior to trial enrolment (yes or no); time from endoscopy to screening; and HBI score. Tertiary time-to-event endpoints were summarised using Kaplan-Meier curves. Safety analyses were summarised by treatment group and biomarker subgroup and presented as incidence (number of patients affected). All statistical analyses were performed using R version 4.3.1. The protocol and statistical analysis plan are provided (appendix 2). An independent data monitoring committee was established for the PROFILE trial, which was registered with the ISRCTN registry, number 11808228.

### Role of funding source

The funders had no role in study design, data collection or analysis, or writing of this report. All authors had access to the final study data, reviewed and approved the final report, and take full responsibility for the accuracy of the data and the statistical analysis. The corresponding author had final responsibility for the decision to submit for publication.

## Results

**Figure 2:**
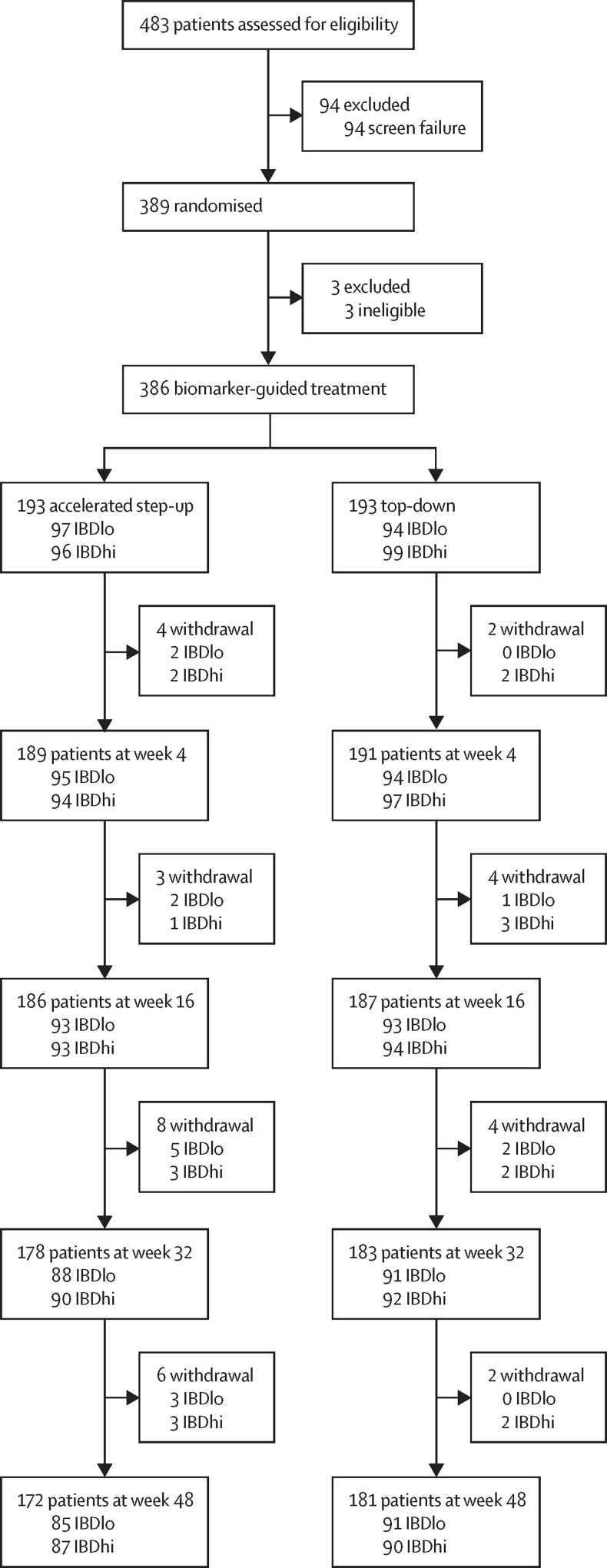
Trial profile

Between Dec 29, 2017, and Jan 5, 2022, 483 patients from 40 hospitals in the UK were screened (appendix p 10). 94 were excluded and 389 patients were randomised to top-down or accelerated step-up treatment, stratified by the biomarker result. Three patients (two in the top-down group and one in the accelerated step-up group) were subsequently excluded for not meeting eligibility criteria, leaving 386 randomised participants (193 in the top-down group and 193 in the accelerated step-up group; figure 2).

Median time from diagnosis to trial enrolment and treatment initiation with the index course of steroids was 12 days (range 0–191). Patient characteristics were similar across all groups, as was baseline disease activity (table 1, appendix 1 pp 11–13).

**Table 1:** Baseline characteristics

		**Step-up group (n=193)**	**Top-down group (n=193)**
**Baseline characteristics**			
Age (years)		34·0 (13·3)	33·3 (13·2)
Sex			
	Female	88/193 (46%)	91/193 (47%)
	Male	105/193 (54%)	102/193 (52%)
Ethnicity			
	White	168/193 (87%)	171/192 (89%)
	Other	25/193 (13%)	21/192 (11%)
Current smoker		42/193 (22%)	49/191 (26%)
Weight (kg)		74·9 (17·5)	74·7 (19·3)
Disease location			
	Ileal	63/193 (33%)	65/192 (34%)
	Colonic	50/193 (26%)	53/192 (28%)
	Ileocolonic	80/193 (41%)	74/192 (39%)
Disease behaviour			
	Inflammatory (B1)	161/190 (85%)	169/192 (88%)
	Stricturing (B2)	27/190 (14%)	22/192 (11%)
	Penetrating (B3)	2/190 (1%)	1/192 (1%)
Mean HBI score		9·8 (2·9)	10·0 (2·9)
Mean CRP (mg/L)		21 (26)	19 (27)
Median CRP (mg/L)		12 (5–24·2)	11 (5–20)
Mean calprotectin (μg/g)		993 (797)	1035 (991)
Median calprotectin (μg/g)		835 (322–>1800)	747 (381–>1800)
Mean SES-CD		10·4 (6·0)	10·9 (6·6)
Median SES-CD		9 (7–13)	9 (7–14)
Steroid course prior to enrolment		40/192 (21%)	30/193 (16%)
Mean time from diagnosis to enrolment (days)		31·2 (40·0)	24·1 (34·4)
Median time from diagnosis to enrolment (days; min–max)		14 (0–191)	9 (0–168)
**Randomisation stratification factors**			
Biomarker status			
	IBDhi	97/193 (50%)	94/193 (49%)
	IBDlo	96/193 (50%)	99/193 (51%)
Disease location			
	Colonic	51/193 (26%)	50/193 (26%)
	Other	142/193 (74%)	143/193 (74%)
Endoscopic inflammation			
	Mild	14/193 (7%)	13/193 (7%)
	Moderate	136/193 (70%)	136/193 (70%)
	Severe	43/193 (22%)	44/193 (23%)

Data are n/N (%), mean (SD), or median (IQR) unless otherwise stated. Baseline characteristics broken down by biomarker status are shown in appendix 1 (pp 11–13)

Primary outcome data were available for analysis in 379 patients (189 in the top-down group and 190 in the accelerated step-up group). All patients in the top-down group received at least one dose of anti-TNF therapy, with 175 (93%) of 189 patients receiving this as combination therapy with an immunomodulator. Median time from randomisation to first dose of infliximab in the top-down group was 15 days (IQR 13–20). 161 (85%) of 190 patients in the accelerated step-up group required escalation to an immunomodulator, with 50% doing so within 100 days of enrolment. Additionally, 77 (41%) patients in the accelerated step-up group had escalated to anti-TNF therapy by week 48.

**Figure 3:**
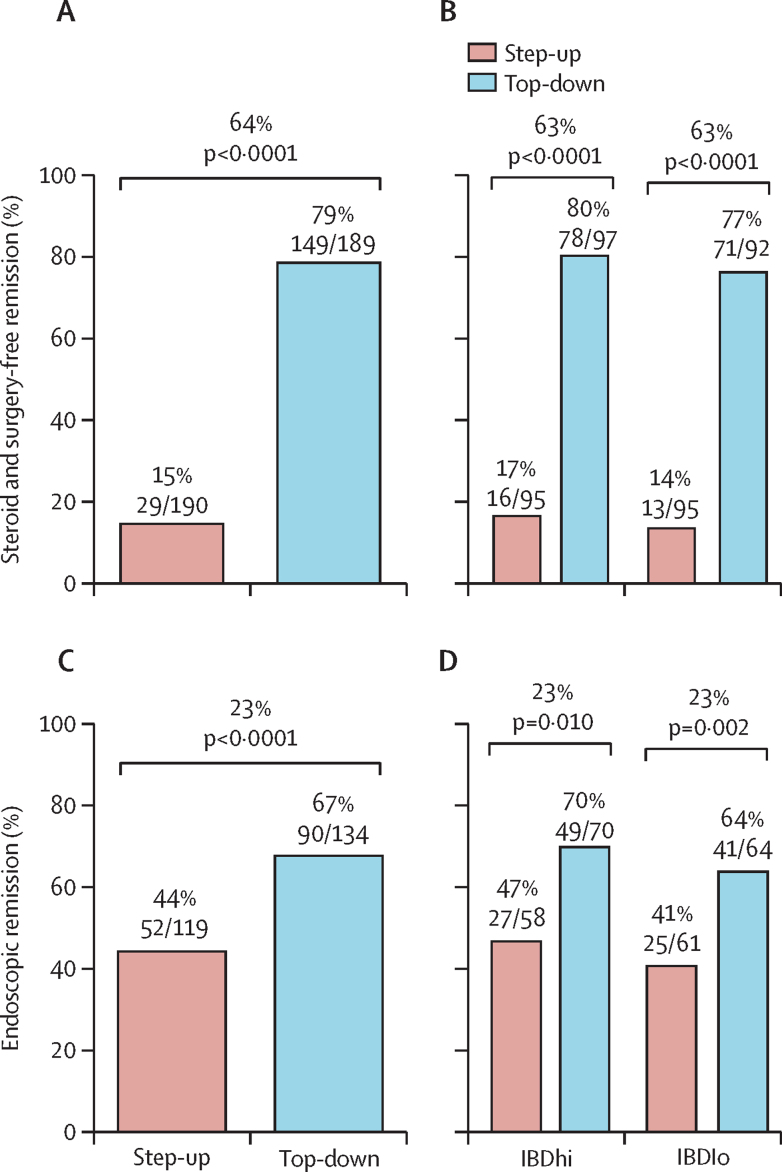
Primary endpoint and key secondary endpoint (A) Sustained steroid-free and surgery-free remission until week 48 for treatment groups. (B) Sustained steroid-free and surgery-free remission until week 48 for biomarker–treatment subgroups. (C) Endoscopic remission (absence of ulceration) at week 48 for treatment groups. (D) Endoscopic remission (absence of ulceration) at week 48 for biomarker-treatment subgroups.

Sustained steroid-free and surgery-free remission to week 48 was more frequent in the top-down group (149 [79%] of 189 patients) than in the accelerated step-up group (29 [15%] of 190 patients), with an absolute difference of 64 percentage points (95% CI 57 to 72; p<0·0001; figure 3). There was no significant biomarker–treatment interaction effect (absolute difference 1 percentage point, 95% CI –15 to 15; p=0·944; table 2).

**Table 2:** Primary and secondary outcomes

		**Treatment effect (difference between groups; 95% CI)**	**p value**
**Top-down *vs* accelerated step-up treatment effect**			
Primary outcome measure			
	Sustained steroid-free and surgery-free remission	64 percentage points (57 to 72)	<0·0001
Secondary outcome measure			
	Endoscopic remission	23 percentage points (11 to 36)	<0·0001
	Quality of life (IBD-Q) numerical score	8·54 (3·51 to 13·60)	<0·0001
	Number of flares	−1·29 (−1·42 to −1·16)	<0·0001
	Number of steroid courses	−0·87 (−0·97 to −0·76)	<0·0001
	Number of hospital admissions and surgeries	−0·12 (−0·23 to −0·02)	0·023
**Biomarker–treatment interaction effect**			
Primary outcome measure			
	Sustained steroid-free and surgery-free remission	1 percentage point (−15 to 15)	0·944
Secondary outcome measure			
	Endoscopic remission	2 percentage points (−24 to 25)	0·902
	Quality of life (IBD-Q) numerical score	1·42 (−8·76 to 11·60)	0·784
	Number of flares	0·06 (−0·33 to 0·20)	0·640
	Number of steroid courses	0·05 (−0·16 to 0·26)	0·638
	Number of hospital admissions and surgeries	−0·11 (−0·32 to 0·11)	0·332

Sustained steroid-free and surgery-free remission and endoscopic remission data are presented as absolute differences (absolute difference in percentage points). Quality of life data are presented as absolute differences using the IBD-Q numerical score. Number of flares, steroid courses, hospital admissions and surgeries are presented as difference in number of events per patient per year.

All secondary outcomes were significantly better in the top-down versus the accelerated step-up, group but there was no evidence of biomarker–treatment interaction for any of them (table 2). Endoscopic remission (SES-CD ulcer subscore of 0) was more frequent in the top-down group (90 [67%] of 134 patients) than in the accelerated step-up group (52 [44%] of 119 patients (table 2, figure 3). Within the accelerated step-up group, endoscopic remission at week 48 was achieved in 24 (35%) of 68 patients who did not escalate to infliximab and 28 (55%) of 51 who did. Data for the secondary endpoints of IBD-Q, number of flares requiring escalation, and cumulative steroid exposure by week 48 in each group are shown in table 2 and appendix 1 (pp 27–30); there were more disease flares, higher need for steroids, and lower quality of life with accelerated step-up treatment than with top-down therapy.

11 urgent abdominal surgeries were required during the trial. One patient was in the top-down group and had gallstone ileus. Nine patients were in the accelerated step-up group, all requiring surgery for obstructive or penetrating complications of Crohn's disease; one patient required two operations (appendix 1 p 14). Six of the nine patients in the accelerated step-up group were classified as having Montreal B1 inflammatory disease at enrolment. The post-hoc odds ratio for needing abdominal surgery was 0·095 (95% CI 0·001–0·505). One patient in each group required surgery for perianal disease. Hospital admission data including surgeries are presented in appendix 1 (p 15).

The most frequent adverse event was a disease flare (table 3, appendix 1 pp 16–17). There were fewer adverse events or serious adverse events in the top-down group than in the accelerated step-up group (adverse events: 168 *vs* 315; serious adverse events: 15 *vs* 42). Further information on adverse events and serious adverse events is reported in table 3. Although not powered to show a difference in safety endpoints, there was no difference in risk of serious infection between treatment strategies and no reported malignancies or deaths during the trial (table 3).

**Table 3:** Adverse events

		**Step-up group (n=193)**	**Top-down group (n=193)**
**Any adverse event**			
Flare of Crohn's disease		225, 132 (68%)	30, 26 (13%)
Infection		20, 12 (6%)	23, 16 (8%)
Thiopurine intolerance		59, 48 (25%)	87, 62 (32%)
Methotrexate intolerance		9, 4 (2%)	8, 6 (3%)
Infliximab intolerance		0	8, 8 (4%)
Malignancy		0	0
Other		2, 2 (1%)	12, 8 (4%)
**Serious adverse events**			
Hospitalisation for flare of Crohn's disease		15, 12 (6%)	3, 3 (2%)
Surgery for disease complication		11, 10 (5%)	2, 2 (1%)
	Abdominal surgery	10, 9 (5%)	1, 1 (1%)
	Perianal surgery	1, 1 (1%)	1, 1 (1%)
Medication related		1, 1 (1%)	1, 1 (1%)
Serious infection		8, 4 (2%)	3, 3 (2%)
Malignancy		0	0
Death		0	0
Other		7, 6 (3%)	6, 4 (2%)

Data are number of events, number of patients (%). Adverse events presented by biomarker status are shown in appendix 1 (p 16).

**Figure 4:**
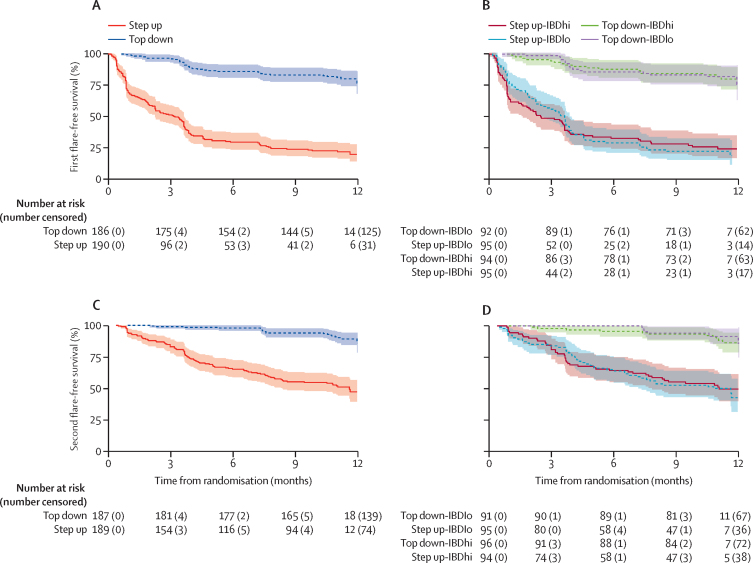
Kaplan-Meier analysis of time to flare, surgery, or both (A) Time to first event by treatment group with data censored at 12 months. (B) Time to first event by biomarker–treatment group with data censored at 12 months. (C) Time to second event by treatment group with data censored at 12 months. (D) Time to second event by biomarker–treatment group with data censored at 12 months. Numbers of patients at risk at 0 months may not match the total number of patients randomised to each group due to missing data.

The time-to-event analyses considered events as a composite of disease flare, treatment escalation, or surgery. Time to both first and second event were longer for participants in the top-down group than in the accelerated step-up group, with no difference between biomarker subgroups (figure 4).

At week 48, endoscopic response (SES-CD improvement ≥50%) was more frequent in the top-down than in the accelerated step-up group (124 [82%] of 151 patients *vs* 79 [63%] of 125 patients; appendix 1 p 18), as was deep endoscopic remission (SES-CD of 0; 81 [52%] of 155 *vs* 49 [37%] of 134; appendix 1 p 19) and in a post-hoc analysis of symptomatic and endoscopic remission (composite HBI <5 plus SES-CD ulcer sub-score of 0; 70 [53%] of 133 *vs* 41 [35%] of 117; appendix 1 p 20). Sustained steroid-free and surgery-free symptomatic remission without biochemical corroboration was also more frequent in the top-down group than in the accelerated step-up group (94 [50%] of 189 *vs* 18 [9%] of 190). A post-hoc analysis of symptomatic remission (HBI <5) at week 48 showed only a modest difference between the treatment arms (136 [78%] of 175 patients in the top-down group *vs* 121 [74%] of 163 in the accelerated step-up group; appendix 1 p 21), while symptomatic plus biochemical remission (composite HBI <5 plus CRP ≤ULN or faecal calprotectin <200 μg/g) was seen in 114 (65%) of 175 patients in the top-down group compared with 64 (41%) of 157 in the accelerated step-up group (appendix 1 p 22). There were notable differences between the treatment groups in the CRP and calprotectin responses throughout the study period, with more rapid (even by week 4) complete and sustained normalisation in the top-down group than in the accelerated step-up group and higher rates of biochemical remission at week 48 (appendix 1 pp 23–25).

The adjustment for baseline covariates in the primary analysis estimated a conditional odds ratio for their influence on the primary endpoint. The likelihood of being in sustained steroid-free and surgery-free remission appeared lower for patients who received steroids before enrolment and higher for those with colonic involvement versus pure ileal disease and longer time from ileo-colonoscopy to trial enrolment (appendix 1 p 26). Moderate or severe inflammation at ileo-colonoscopy appeared to be associated with a higher likelihood of remission (appendix 1 p 26). None of the other baseline covariates were associated with outcome, including CRP, faecal calprotectin, smoking status, or BMI (appendix 1 p 26); and no covariates had an interaction with treatment response or biomarker–treatment interaction.

The conclusions of the modified per-protocol analysis were consistent with the full analysis across all endpoints (data not shown). 351 (91%) of 386 participants (164 [85%] patients in the accelerated step-up group and 187 [97%] in the top-down group) adhered to the PROFILE treatment protocol. A sensitivity analysis to assess the impact of COVID-19 showed no significant difference for any outcome measure between pre-pandemic (n=104) and peri-pandemic populations (n=285; data not shown). Primary endpoint data were missing for seven of 386 of participants—ie, less than 2%. Data completeness was similarly good for all secondary outcomes apart from endoscopic remission. With most endoscopy units closing during the pandemic,^20^ end-of-trial ileo-colonoscopies were not performed in 133 (34%) of 386 participants. These data were analysed as missing at random. A sensitivity analysis comparing CRP and calprotectin between participants who did or did not undergo end-of-trial ileo-colonoscopy did not show a systematic difference (appendix 1 p 31). 166 (66%) of 253 end-of-trial colonoscopies were video-recorded and scored by central readers. Week 48 endoscopic remission (absence of ulceration) restricted to these was again more frequent in the top-down group than in the accelerated step-up group (55 [60%] of 92 *vs* 33 [45%] of 74; appendix 1 p 32) but attenuated compared with the whole group.

## Discussion

The PROFILE trial produced two key findings. First, the blood-based biomarker that the study was primarily designed to test did not show clinical utility for guiding therapy in Crohn's disease. Second, the results demonstrated that a top-down treatment approach, initiated at the time of diagnosis, was both highly effective and safe. Compared to a standard accelerated step up approach, top-down treatment resulted in a 64 percentage point difference in sustained steroid-free and surgery-free remission throughout the study period and substantial benefits across all secondary endpoints including endoscopic remission.

These findings are potentially transformative for the management of Crohn's disease. The need for a prognostic biomarker was predicated on the lack of an effective, safe, and affordable treatment strategy for newly diagnosed patients. While PROFILE did not identify a clinically useful biomarker, it has provided clear evidence with regards to the optimal treatment strategy from diagnosis. Indeed, the scale of the benefit with top-down management quantified in PROFILE would, if sustained, substantially weaken the case-of-need for a prognostic biomarker.

With patients randomised between top-down and accelerated step-up groups, the treatment outcomes from PROFILE build on evidence from previous trials regarding the benefits of early anti-TNF therapy.^10^, ^12^ Despite these earlier trials, use of biologics from diagnosis remains unusual outside specialist centres, probably reflecting uncertainty regarding treatment timing and concerns regarding cost and safety.^21^ Instead reactive accelerated step-up therapy is the norm, as advocated by some (but not all) international guidelines.^13^, ^14^, ^15^

In PROFILE, the markedly higher frequency and rate at which patients relapsed and required treatment escalation in the accelerated step-up arm was highlighted in the time-to-event analysis (figure 4). Substantial differences were also observed for all secondary endpoints. The endoscopic remission rate of 67% seen in the top-down group at week 48 is greater than almost all previous Crohn's disease trials, which have typically reported rates of approximately 30% at 1 year.^22^ Endoscopic remission even in the accelerated step-up group was an impressive 44% (55% in those who escalated to infliximab and 35% in those who did not), highlighting the benefits of early intervention. The smaller delta for endoscopy outcomes compared with the primary endpoint reflects persisting subclinical endoscopic activity.

Endoscopic remission is well recognised to correlate with better long-term outcomes, including reduced need for surgery.^23^ Notably in PROFILE abdominal surgery was required in just one patient in the top-down group, for gallstone ileus, whereas nine patients in the accelerated step-up group required intestinal resection for stricturing or fistulating complications. These endoscopic and surgical outcomes are particularly important in being less influenced by the study design and open treatment allocation than the primary outcome, and thus provide important context for the primary endpoint analysis. Clinicians know that biologics effectively maintain remission, and hence the difference between treatment arms for the primary outcome might have been expected. The benefit of top-down treatment in reducing need for surgery by a factor of approximately 10 even within the first year and despite widespread early therapy escalation in the accelerated step-up group may be more surprising. This further underlines the importance of initiating highly effective treatment to control inflammation as soon as possible after diagnosis. Population-level data corroborate this: even in the most recent eras more than 10% of (conventionally managed) patients require surgery within a year of diagnosis of Crohn's disease.^24^

In building on the findings from the REACT and CHARM trials, which both correlated earlier biologic therapy with decreased need for surgery albeit in more longstanding disease,^11^, ^25^ the results from PROFILE cast doubt on the historic suggestion that aggressive early treatment with anti-TNF therapy incurs unnecessary risks. The reality appears to be the converse. Inadequate treatment of active disease confers the greatest risks to individual patients, particularly young adults who are most commonly affected by Crohn's disease and in whom serious drug side-effects are rare. Moreover, with a median time from diagnosis to enrolment of just 12 days and then a median time from randomisation to first dose of infliximab of 15 days in the top-down group, PROFILE provides previously unappreciated insights into the window of opportunity in Crohn's disease and how very early advanced therapy can maximise benefit for patients.

Of note, the large treatment effect in PROFILE was demonstrated across the severity spectrum. Inclusion required biochemical and endoscopic evidence of active inflammation but not all participants had moderate to severe disease—conventionally viewed as the gateway to advanced therapy. The magnitude of the treatment benefit might reflect this breadth of severity as well as the short disease duration, perhaps catching mucosal immune dysfunction at a stage when it was still amenable to rectification.^26^

Several clinical factors have previously been suggested to correlate with Crohn's disease course and treatment response. The covariates used in the primary analysis provide some information about these, although interpretation requires caution due to their observational nature. None appeared to affect the treatment response or biomarker–treatment interaction, but some did show evidence of association with disease outcomes (appendix 1 p 25). 70 (18%) of participants had completed a steroid course in the 6 months before trial enrolment; these patients appeared (perhaps expectedly) to have a lower likelihood of maintaining remission during the trial, as did those with exclusively ileal involvement. Counterintuitively, patients with moderate or severe mucosal inflammation at baseline ileo-colonoscopy had a higher likelihood of remission compared to those with mild inflammation, although only 7% of participants were mildly inflamed, which limits interpretation of this finding. There was no association with outcome for CRP, faecal calprotectin, smoking status, age, or BMI (appendix 1 p 26).

PROFILE is the first randomised trial in IBD to use a biomarker-stratified interventional design. The biomarker had been developed to predict a CD8 T-cell transcriptional signature previously correlated with need for treatment escalation in IBD.^27^ This signature related to differences in T-cell exhaustion, providing a plausible biological rationale for different clinical outcomes.^27^, ^28^ Care had been taken during biomarker development to prevent over-fitting and independently validate associations with clinical outcome in two real-world cohorts.^17^ In the event, the associations were not observed in the larger PROFILE population.

Failure to demonstrate clinical utility underlines why randomised trials are critical to test potential biomarkers and why they should be a mandatory step before clinical implementation. The exact reasons for the biomarker failing remain unclear, but may include inaccurate prediction of the CD8 transcriptional signature,^14^ unknown confounders that were better distributed across biomarker and treatment groups in PROFILE than previous observational cohorts, and the high rate of early treatment intensification in the accelerated step-up group, particularly in the IBDlo biomarker subgroup, where lower relapse rates had been expected. The latter may reflect closer monitoring and a lower threshold for treatment escalation in PROFILE compared to the real-world practice on which the biomarker development was based. The comparability of discovery and validation cohorts critically determines whether a predictive model will be generalisable in other cohorts.^29^ While the failure to demonstrate clinical utility in PROFILE can inform future attempts to validate biomarkers for personalised medicine, it should not preclude them.

PROFILE had a number of limitations. First, treating clinicians were blinded to the biomarker group but not treatment allocation, potentially resulting in over-estimation of the treatment benefit of the top-down approach. Willingness to diagnose a flare may have been biased according to treatment group, although the requirement for objective evidence of inflammation alongside symptoms was designed to mitigate this. Second, patients with normal inflammatory markers, minimal symptoms, or minimal inflammation at baseline ileo-colonoscopy were excluded. Whether such patients would also benefit from top-down therapy is unknown. Third, therapeutic drug monitoring and dose optimisation, which might have further enhanced the efficacy of infliximab, were not included in the trial protocol. Fourth, during the COVID-19 pandemic use of remote consultations adversely affected blood and stool sampling, although high levels were still maintained (appendix 1 p 4). Fifth, a third of patients did not have an end-of-trial ileo-colonoscopy, largely due to pandemic-related service shutdowns,^20^ although no systematic differences were seen in inflammatory markers between patients undergoing or not undergoing end-of-trial ileo-colonoscopy (appendix 1 p 31). Additionally, 89 (35%) of 255 patients had their end-of-trial procedures scored only by unblinded local investigators. The difference in endoscopic remission rates between top-down and accelerated step-up approaches was 15 percentage points if only centrally read procedures were considered, and 23 percentage points in the whole group, suggesting confirmation bias in local endoscopy reading. Finally, although the high rates of endoscopic remission seen with top-down treatment suggest that the benefits of this strategy in reducing the need for surgery should be durable and potentially cost-effective well beyond 1 year, the relevant long-term follow-up data and health economic analyses are not yet available.

PROFILE also had several key strengths. It robustly tested the biomarker and is the first randomised trial in Crohn's disease to have compared top-down and accelerated step-up treatment strategies from diagnosis. Participant inclusion required active symptoms plus objective biochemical and endoscopic evidence of active inflammation, and a wide spectrum of disease severity was represented. Meeting the primary endpoint required participants to remain in remission throughout the study period, rather than at one or two timepoints. The benefit of top-down treatment across all secondary endpoints underpins confidence in the result. A unique feature of PROFILE was the short time (median 12 days) from diagnosis to enrolment and treatment initiation. This provides previously unappreciated insights into the true efficacy of early top-down therapy.^30^

PROFILE raises important questions for future research. Longer term follow-up could inform the need for continued immunomodulator therapy alongside infliximab. Given the logistics and costs of intravenous drug delivery, relevant questions are whether subcutaneous infliximab or alternative, cheaper formulations of anti-TNF would show the same benefits. This would potentially improve access globally. Another question is whether the benefit seen in the anti-TNF-based top-down group would also be seen with other advanced therapies or combinations.

PROFILE provides definitive evidence for the benefit of top-down over accelerated step-up treatment, at least for patients meeting the trial inclusion criteria of active symptoms, raised CRP or calprotectin of 200 μg/g or more, plus active inflammation on ileo-colonoscopy. Given that this definition encompasses the majority of patients newly presenting with Crohn's disease, the case appears clearcut for implementation of top-down treatment as the standard of care for most patients as soon as possible after diagnosis.

## Data sharing

Access to the trial data and other materials is managed by the Cambridge University Hospitals R&D Study Review Committee. Please contact the corresponding author.

## Declaration of interests

NMN reports personal fees from Galapagos, Janssen, Lilly, SBK Healthcare, and Takeda outside the submitted work; and grants from Dr Falk, Pfizer, Pharmacosmos, and Tillotts Pharma outside the submitted work. JCL reports consultancy fees from AbbVie, AgPlus Diagnostics, PredictImmune, and C4X Discover outside the submitted work; and grants from GSK outside the submitted work. KVP reports personal fees from AbbVie, DrFalk, Galapagos, Janssen, PredictImmune, Pfizer, and Takeda outside the submitted work; grants from AbbVie, Celltrion, Dr Falk, Ferring, Janssen, Takeda, and Tillotts Pharma outside the submitted work; and fees from advisory board membership of AbbVie, Galapagos, Janssen, and Pfizer outside the submitted work. TA reports personal fees from Amgen, Celltrion, Janssen, Lilly, Pfizer, and Tillotts Pharma; and grants from Biogen, Celltrion, Galapagos, Nova Pharmaceuticals, Pfizer, Roche, and Takeda outside the submitted work. JWB reports personal fees from Janssen outside the submitted work. RC reports personal fees from AbbVie, Galapagos, and Lilly outside the submitted work, grants from Celltrion, Ferring, and Tillotts Pharma outside the submitted work; and fees from advisory board membership of AbbVie and Bristol Myers Squibb outside the submitted work. SD reports personal fees from AbbVie, Ferring, Janssen, and Takeda outside the submitted work; grants from AbbVie, Dr Falk, and Janssen, outside the submitted work; and fees from advisory board membership of AbbVie outside the submitted work. DD reports personal fees from Takeda outside the submitted work; and grants from AbbVie and Janssen outside the submitted work. PMI reports personal fees from AbbVie, Arena, Boehringer-Ingelheim, BMS, Celgene, Celltrion, Dr Falk, Galapagos, Genentech, Gilead, Hospira, Janssen, Lilly, MSD, Pfizer, Prometheus, Sandoz, Samsung Bioepis, Sapphire Medical, Takeda, Topivert, VH2, Vifor Pharma, and Warner Chilcott outside the submitted work; and grants from AbbVie and Takeda outside the submitted work. AJK reports personal fees from AbbVie, Galapagos, and Takeda outside the submitted work; grants from AbbVie, Janssen, and Tillotts Pharma outside the submitted work; and fees from advisory board membership of AbbVie and Janssen outside the submitted work. CM reports grants from Janssen outside the submitted work. CSP reports personal fees from Dr Falk outside the submitted work. TR reports personal fees from AbbVie, Arena, Aslan, AstraZeneca, Boehringer-Ingelheim, BMS, Eli Lilly, Ferring, Galapagos, Gilead, GSK, Heptares, LabGenius, Novartis, Numab, Janssen, Pfizer, Roche, Takeda, UCB, and XAP therapeutics outside the submitted work; grants from AbbVie outside the submitted work; and fees from data monitoring board membership of UCB outside the submitted work. SS reports personal fees from AbbVie, Celltrion, Dr Falk, Ipsen, Janssen, and Takeda outside the submitted work. HRTW reports fees from advisory board membership of Pfizer outside the submitted work. SV reports personal fees from AbbVie, Abivax, AbolerISPharma, AgomAb, Alimentiv (formerly Robarts Clinical Trials), Arena Pharmaceuticals, AstraZeneca, BMS, Boehringer Ingelheim, Celgene, Cytoki Pharma, Dr Falk Pharma, Ferring, Galapagos, Genentech-Roche, Gilead, GSK, Hospira, Imidomics, Janssen, J&J, Lilly, Materia Prima, Mestag Therapeutics, MiroBio, Morphic, MrMHealth, MSD, Mundipharma, Pfizer, Prodigest, Progenity, Prometheus, Surrozen, Takeda, Theravance, Tillotts Pharma, VectivBio, Ventyx, and Zealand Pharma outside the submitted work; and grants from AbbVie, Galapagos, J&J, Pfizer, and Takeda outside the submitted work. VJ reports personal fees from AbbVie, Alimentiv (formerly Robarts Clinical Trials), Arena Pharmaceuticals, Asahi Kasei Pharma, Asieris, AstraZeneca, Avoro Capital, BMS, Celltrion, Endpoint Health, Enthera, Ferring, Flagship Pioneering, Fresenius Kabi, Galapagos, Gilde Healthcare, GSK, Genentech, Gilead, Innomar, JAMP, Janssen, Lilly, Merck, Metacrine, Mylan, Pandion, Pendopharm, Pfizer, Protagonist, Prometheus, Reistone Biopharma, Roche, Roivant, Sandoz, SCOPE, Second Genome, Sorriso Pharmaceuticals, Takeda, Teva, Topivert, Ventyx, and Vividion outside the submitted work; and fees from advisory board membership of AbbVie, Alimentiv (formerly Robarts Clinical Trials), Arena Pharmaceuticals, Asahi Kasei Pharma, Asieris, AstraZeneca, BMS, Celltrion, Ferring, Flagship Pioneering, Fresenius Kabi, Galapagos, Gilde Healthcare, GSK, Genentech, Gilead, Innomar, JAMP, Janssen, Lilly, Merck, Metacran, Mylan, Pandion, Pendopharm, Pfizer, Protagonist, Prometheus, Reistone Biopharma, Roche, Sandoz, SCOPE, Second Genome, Sorriso Pharmaceuticals, Takeda, Teva, Topivert, Ventyx, and Vividion outside the submitted work. GRDH reports personal fees from AbbVie, Alimentiv, AstraZeneca, Immunic, J&J, Lilly, Pfizer, Takeda, Tillotts, and Ventyx outside the submitted work; grants from AbbVie, BMS, Lilly, Pfizer, and Takeda outside the submitted work; and fees from advisory board membership of AstraZeneca, Galapagos, and Seres Health outside the submitted work. EFM reports being a co-founder and receiving consultancy fees from PredictImmune; and holds PredictImmune stock or stock options. PAL reports being a co-founder and receiving consultancy fees from PredictImmune; and holds PredictImmune stock or stock options. JOL reports personal fees from AbbVie, Atlantic Healthcare, Bristol Meyer Squibb, Celgene, Celltrion, Engytix, Eli Lilly, Ferring, Galapagos, Gilead, GSK, Janssen, MSD, Norgine, Pfizer, Shire, and Takeda outside the submitted work; and grants from AbbVie, Ferring, Gilead, Takeda, and Shire outside the submitted work. NAK reports personal fees from Amgen, Bristol Myers Squibb, Celltrion, Falk, Galapagos, Janssen, Pfizer, Pharmacosmos, Takeda, and Tillotts Pharma outside the submitted work; and reports data monitoring board membership for the BEACON study outside the submitted work. KGCS reports being a co-founder and receiving consultancy fees from Predictimmune; consultancy fees from GSK outside the submitted work; and holds PredictImmune stock or stock options. MP reports personal fees from Janssen and Takeda outside the submitted work; and grants from AstraZeneca, Galapagos, Gilead, and Pfizer outside the submitted work. All other authors declare no competing interests.
