# Correction to *Lancet Gastroenterol Hepatol* 2024; 9: 415–27

**DOI:** 10.1016/S2468-1253(25)00203-1

**Published:** 2025-07-09

*Noor NM, Lee JC, Bond S, et al, on behalf of the PROFILE Study Group. A biomarker-stratified comparison of top-down versus accelerated step-up treatment strategies for patients with newly diagnosed Crohn's disease (PROFILE): a multicentre, open-label randomised controlled trial.* Lancet Gastroenterol Hepatol *2024; **9:** 415–27*—In this Article, the affiliation information for James O Lindsay should also have included the Blizard Institute, Barts and the London School of Medicine, Queen Mary University of London, London, UK. This correction has been made as of July 9, 2025.

