# Supplementary material for: A biomarker-stratified comparison of top-down versus accelerated step-up treatment strategies for patients with newly diagnosed Crohn's disease (PROFILE): a multicentre, open-label randomised controlled trial
Source: Lancet Gastroenterol Hepatol. 2024 Feb 22;9(5):415–27. doi: 10.1016/S2468-1253(24)00034-7 (PMC11001594; doi:10.1016/S2468-1253(24)00034-7)
Supplement: Supplementary appendix 1 [file mmc1.pdf]

THE LANCET
Gastroenterology & Hepatology

Supplementary appendix 1

This appendix formed part of the original submission and has been peer reviewed. We post it as supplied by the authors.

Supplement to: Noor NM, Lee JC, Bond S, et al. A biomarker-stratified comparison of top-down versus accelerated step-up treatment strategies for patients with newly diagnosed Crohn's disease (PROFILE): a multicentre, open-label randomised controlled trial. *Lancet Gastroenterol Hepatol* 2024; published online Feb 22. https://doi.org/10.1016/S2468-1253(24)00034-7.

Appendix

Table of Contents

List of PROFILE Study Group. 2

Supplementary methods. 3

Supplementary tables and figures 5

- **Table S1. Inclusion and exclusion criteria.** 5
- **Table S2. Outcome for all combinations of HBI score, CRP and calprotectin.** 7
- **Table S3. The list of tertiary endpoints in PROFILE.** 8
- **Table S4. Participating sites and principal investigators ordered by number of participants recruited.** 10
- **Table S5. Baseline demographics table including biomarker subgroups.** 11
- **Table S6. Patients with surgical operations during PROFILE.** 14
- **Table S7. Hospital admissions and surgeries during PROFILE.** 15
- **Table S8. Adverse events in PROFILE including biomarker subgroups.** 16
- **Figure S1. Incidence and relative risk of adverse events.** 17
- **Figure S2. Endoscopic response (>50% improvement in SES-CD score) at week 48.** 18
- **Figure S3. Deep endoscopic remission (total SES-CD score=0) at week 48.** 19
- **Figure S4. Composite of clinical remission (HBI score <5) and endoscopic remission (absence of ulcers) at week 48.** 20
- **Figure S5. Clinical remission alone (HBI score <5) at week 48.** 21
- **Figure S6. Composite of clinical remission (HBI score <5) and biochemical remission (CRP <ULN and calprotectin <200ug/g) at week 48.** 22
- **Figure S7. Median CRP.** 23
- **Figure S8. Median faecal calprotectin.** 24
- **Figure S9. Biochemical remission (CRP <ULN and calprotectin <200ug/g) at week 48.** 25
- **Figure S10. Forest plot of the association between clinical variables and the primary endpoint independent of treatment or biomarker subgroup.** 26
- **Figure S11. Disease flares requiring treatment escalation.** 27
- **Figure S12. Additional course(s) of steroid after initial induction course.** 28
- **Figure S13. Mean quality-of-life numerical score (using IBD-Q).** 29
- **Figure S14. Quality-of-life remission (IBD-Q >170).** 30
- **Figure S15. CRP and calprotectin for those who had end of trial ileo-colonoscopy versus those who did not have end of trial ileo-colonoscopy.** 31
- **Figure S16. Endoscopic remission (absence of ulcers) at week 48 using centrally-read videos only.** 32

List of PROFILE Study Group.

**Core writing group**
Nurulamin M. Noor, James C. Lee, Simon Bond, Kamal V. Patel, Klaartje Bel Kok, Shahida Din, James O. Lindsay, Nicholas A. Kennedy, Kenneth G.C. Smith, Miles Parkes.
**Wider writing and review group (alphabetical order)**
Tariq Ahmad, Paul J. Banim, James W. Berrill, Biljana Brezina, Rachel Cooney, Geert R. D'Haens, Juan De La Revilla Negro, Shanika de Silva, Francis Dowling, Dharmaraj Durai, John N. Gordon, Peter M. Irving, Vipul Jairath, Matthew Johnson, Alexandra J. Kent, Paul A. Lyons, Eoin F. McKinney, Gordon W. Moran, Craig Mowat, Pritash Patel, Christopher S. Probert, Tim Raine, Rebecca Saich, Abigail Seward, Dan Sharpstone, Melissa A. Smith, Sreedhar Subramanian, Sara Upponi, Gijs R. van den Brink, Severine Vermeire, Alan Wiles, Horace R.T. Williams.
**Banner authorship (alphabetical order)**
Clare Allcock, Suhaylah Bhatti, Jonathan Blackwell, Robert Boulton-Jones, Matthew J. Brookes, Rhys Butcher, Jeffrey Butterworth, Karlena Champion, Rakesh Chaudhary, Andrew T. Cole, Lauranne A.A.P. Derikx, Anjan Dhar, Mary Flowerdew, Rishi Goel, Ailsa L. Hart, Rory Hughes, Babur Javaid, Paul Knight, Jacinta Lee, Charlie W. Lees, Emma Levell, Andy Li, Charles D. Murray, Leisha O'Brien, Gareth Parkes, Richard C. Pollok, Sam Powles, Arvind Ramadas, Philip J. Smith, R. Alexander Speight, Simon P.L. Travis, Sean Weaver, Emma Wesley.

Supplementary methods.

Procedures

The biomarker is a weighted logistic regression model comprising 15 informative genes and 2 control genes, identified using an elastic net method to optimise combined LASSO and RIDGE regularisation parameters during iterative feature selection and weight optimisation steps. Differential weighting of the 15 genes means they make distinct contributions to the model result. After 30 patients had been randomised the algorithm weightings were adjusted following an algorithm update. Two of the existing participants' biomarker value were changed. The PROFILE trial management group, trial steering committee and all site principal investigators remained blinded to the biomarker results throughout and a decision was taken by the steering committee to accept the modified weightings on 10th September 2018. The biomarker weightings were then formally modified on 1st October 2018. Prior to 28th March 2019 sample processing was carried out in the Department of Medicine, University of Cambridge School of Clinical Medicine, Cambridge University Hospitals NHS Foundation Trust. After this date sample processing was performed at the East Genomic Laboratory Hub, Cambridge University Hospitals NHS Foundation Trust until the end of the trial.

The medical dictionary for regulatory activities (meDRA) was used to code adverse events. Each AE and SAE was reviewed prior to allocation to an appropriate category.

Statistical analysis

For missing baseline covariates, Multivariate Imputations using Chain Equations were used to provide five imputed complete data sets. This was repeated separately for each endpoint/analysis as the endpoint was used as predictor of the missing baseline values. For any longitudinal analysis of repeated observations, the per-patient average of the repeated observations was used, along with an average of the visit numbers with observations. Rubin's rules were used to combine the analysis from the multiple imputed data sets. Where bootstrapping was used to provide confidence intervals around standardised effects on the additive scale, then bootstrapping was performed as an outer loop around repeated multiple imputations.

Based on reviewer suggestion, additional post-hoc analysis was undertaken for selected additional exploratory endpoints - for clinical remission alone at week 48; and composite endpoints of clinical plus biochemical remission, and clinical plus endoscopic remission at week 48. These analyses were performed and considered in line with the unranked tertiary endpoints already listed in the SAP, without statistical significance testing being performed.

During the COVID pandemic many patient ‘visits’ were conducted remotely. Participants were then invited to submit a stool sample or attend for phlebotomy separately but at a time as close to the visit as possible. Biochemistry (CRP/calprotectin) data to corroborate symptoms (HBI) was almost complete at all trial visits. For example, only one patient (1%) in “top-down” and seven (4%) in “accelerated step-up” had an HBI score without either CRP or faecal calprotectin measured at week 48. Scheduled sample return rates and processing with regards to faecal calprotectin were broadly satisfactory: week 16 (80% in both “accelerated step-up” and “top-down” arms), week 32 (82% in “accelerated step-up” and 81% in “top-down”), and week 48 (69% “accelerated step-up” and 79% in “top-down”).

Supplementary tables and figures

Table S1. Inclusion and exclusion criteria.

**Inclusion criteria**
Patients to be included in the trial needed to meet the following criteria:<br>• Crohn's disease diagnosed within 6 months* using standard endoscopic, histologic or radiological criteria**<br>• Clinical evidence of active Crohn's disease (corresponding to an HBI $\geq$ 7)<br>• Endoscopic evidence of active Crohn's disease***<br>• CRP > upper limit of normal on local assay OR Calprotectin $\geq$ 200 µg/g****<br>• Immunomodulator and anti-TNFα naïve<br>• Aged 16-80 years old<br><br>* Patients with newly-diagnosed patchy colonic inflammation, initially diagnosed as indeterminate colitis, would meet inclusion criteria for the trial if felt to be consistent with Crohn's disease as judged by local investigators.<br><br>** Patients needed to have discontinued systemic steroids for one week or more prior to screening assessments and still have ongoing, active disease.<br><br>*** Grading of severity was based on clinical impression of endoscopist or clinical team managing Crohn's disease and as a guide was expected to correspond to an approximate SES-CD of 4 or more for ileal-only disease and score of approximately 6 or more for ileocolonic or colonic disease distributions.<br><br>**** Results of blood tests including CRP were determined at local laboratories. Based on differences between assays used for CRP, a figure more than the upper limit of normal was selected for inclusion rather than any specific CRP value. The trial protocol required a stool sample to be sent for central processing but in cases where a sample was not received or where there was no central laboratory value, results from local faecal calprotectin samples could be used.
**Exclusion criteria**
The presence of any of the following will preclude patient inclusion:<br>• Patients with ulcerative colitis.<br>• Patients with active perianal sepsis or fistulating peri-anal Crohn's disease sufficient to mandate anti-TNF therapy (as judged by local investigators).<br>• Patients with obstructive symptoms AND evidence of a fixed stricture on radiology or colonoscopy, which suggest that the patient is at high risk of requiring surgery over the following year. N.B. patients with modest degrees of stricturing on imaging but no obstructive symptoms may be included according to clinician judgement.<br>• Patients with contra-indications to trial medications.

- Patients with blood results that contra-indicate the medications used in the trial including a history of hepatitis B or C, tuberculosis.
- Patients with active malignancy or recent malignancy with clinically estimated high risk of recurrence.
- Patients who are pregnant or breastfeeding at baseline.
- Other serious medical or psychiatric illness currently ongoing, or experienced in the last 3 months.
- Patients unable to comply with protocol requirements (for reasons including alcohol and/or recreational drug abuse).

Table S2. Outcome for all combinations of HBI score, CRP and calprotectin.

HBI score	CRP	Calprotectin (ug/g)	Outcome
<5	<=ULN	<=200	Remission
<5	<=ULN	Missing	Remission
<5	Missing	<=200	Remission
<5	Missing	Missing	Missing
<5	<=ULN	>200	Remission
<5	>ULN	<=200	Remission
<5	>ULN	>200	Remission
<5	>ULN	Missing	Remission
<5	Missing	>200	Remission
>=5	<=ULN	<=200	Remission
>=5	<=ULN	Missing	Remission
>=5	Missing	<=200	Remission
>=5	Missing	Missing	Missing
>=5	<=ULN	>200	Flare
>=5	>ULN	<=200	Flare
>=5	>ULN	>200	Flare
>=5	>ULN	Missing	Flare
>=5	Missing	>200	Flare

ULN=upper limit of normal. CRP=C-reactive protein. HBI=Harvey Bradshaw Index.

Remission at each visit is a composite of two conditions:

- HBI score <5
- Absence of objective evidence of inflammation: both CRP<=ULN and calprotectin<200ug/g.
    - If both values are missing then the condition is deemed missing. If just one value is missing then it is assumed to be below the threshold.

If either or both conditions hold then the participant is in remission at the visit.

Table S3. The list of tertiary endpoints in PROFILE.

- Incidence of sustained surgery and steroid free remission from completion of a standard (maximum 8-week regimen) steroid induction treatment through to week 48 (when remission defined using clinical parameters alone, HBI < 5).
- Clinical remission (defined as HBI < 5) at weeks 4,16, 32 and 48.
- Biochemical remission (defined as CRP $\leq$ ULN and calprotectin <200) at weeks 4,16,32 and 48.
- CRP response (comparison of mean CRP scores in each group) at weeks 4,16,32 and 48.
- Calprotectin response (comparison of mean calprotectin scores in each group) at weeks 4,16,32 and 48.
- Incidence of 2 or more treatment escalations for flares of Crohn's disease.
- Time to event, time from baseline to first flare or need for surgery for Crohn's disease, which may occur during the protocolised induction course of steroid medication.
- Time to event, time from baseline to second flare or need for surgery for Crohn's disease.
- Time to event, time from baseline to starting on anti-TNF therapy for Crohn's disease.
- Patient reported clinical remission (using score generated from abdominal pain and stool frequency components of HBI score – abdominal pain $\leq$1 (none or mild) and stool frequency $\leq$3) at weeks 4,16,32,48.
- Steroid free clinical remission (defined as HBI < 5 and no current use of or plan to prescribe steroids) at weeks 4,16,32,48.
- Steroid-free biochemical remission (defined as CRP $\leq$ULN and calprotectin <200 and no current use of or plan to prescribe steroids) at weeks 4,16,32,48.
- Steroid-free endoscopic remission (defined as absence of ulceration i.e. ulcer subscore=0 and no current use of or plan to prescribe steroids) at week 48.
- Endoscopic remission at week 48 using video from end of trial. Defined by ulcer subscore=0 using central-reads from videos only.
- Endoscopic remission at week 48 using video and images from end of trial. Defined by ulcer subscore=0 using central-reads from videos and images.
- Endoscopic remission at week 48 using video and images from end of trial. Defined by ulcer subscore=0 using central-reads from videos and images where available, in combination with local-reads (whenever video or imaging central reads not available).
- Endoscopic remission at week 48 incorporating total SES-CD score. Defined by ulcer subscore=0 + SES-CD score <4, using central-reads from videos where available, in combination with local-reads (whenever video central reads not available).
- Endoscopic remission at week 48 defined by ulcer subscore=0 + SES-CD score <4, using only locally-read endoscopic scores.
- Endoscopic response (defined by SES-CD drop of $\geq$50% from baseline SES-CD score) at week 48 using only locally-read scores from all participants.
- Deep endoscopic remission (defined by total SES-CD score of 0) at week 48, using centrally-read videos where available, in combination with local-reads when video central reads not available.

- Deep endoscopic remission (defined by total SES-CD score of 0) at week 48, using only locally-read endoscopic scores.
- Endoscopic remission at week 48 in only those who had ulcers at the index colonoscopy (i.e. ulcer subscore of ≥1 on the index colonoscopy). Endoscopic remission defined as absence of ulceration i.e. ulcer subscore= 0). Centrally-read endoscopic scores will be used where available, and locally-read scores will be used only if central scores are not available.
- IBD-specific quality of life remission (defined by IBD-Q score of ≥170) at weeks 16,32 and 48.
- IBD-specific quality of life improvement/response (defined as IBD-Q increase of ≥16 from screening visit IBD-Q score) at weeks 16,32 and 48.
- Generic quality of life response (comparison of mean EQ-5D scores in each group) at each of weeks 16,32,48.
- Generic quality of life improvement/response (defined as EQ-5D increase of ≥10% from the screening visit EQ-5D score) at weeks 16,32,48.
- IBD-Q bowel symptom improvement/response (≥8 increase in IBDQ bowel symptom domain from the screening visit) at weeks 16,32,48. Bowel symptoms are questions 1,5,9,13,17,20,22,24,26,29
- IBD-Q fatigue (question 2) improvement/response (≥1 increase in IBDQ fatigue symptom domain from the screening visit) at weeks 16,32,48.
- Weight.
- Blood Cell Counts (Haemoglobin, White cell count, Neutrophil count) at weeks 4,16,32,48.
- Biochemical levels (albumin) at weeks 4,16,32,48.
- Metabolite levels (6TGN & 6MMP) at weeks 16,32,48.
- Perianal disease (4 non-exclusive classifications of: anal tag, anal fissure, anal fistula, perianal abscess) at weeks 4,16,32,48.
- Development of peri-anal abscess or fistula (development of peri-anal abscess / fistula vs no development of peri-anal abscess / fistula) at weeks 4,16,32,48.
- Development of endoscopic stricture by week 48 (development of stricture vs no development of stricture).
- Anti-TNF therapy at last observation: no anti-TNF therapy; monotherapy anti-TNF; combination therapy anti-TNF. Determined from adjudication of CRF data.
- Thiopurine at last observation within each participant: no thiopurine; optimised metabolite levels (6-TGN ≥235); non-optimised levels (6-TGN <235).

Table S4. Participating sites and principal investigators ordered by number of participants recruited.

Site number	Site name	Site Principal Investigator
1	Cambridge University Hospitals, Cambridge	Nurulamin Noor
2	St George's Hospital, London	Kamal Patel
3	Barts Health, London	Klaartje Bel Kok
4	Western General Hospital, Edinburgh	Shahida Din
5	Liverpool University Hospital, Liverpool	Christopher Probert
6	Royal Hampshire County Hospital, Winchester	John Gordon
7	Russells Hall Hospital, Dudley	Shanika de Silva
8	Royal Sussex County Hospital, Brighton	Melissa Smith
9	King's College Hospital, London	Alexandra Kent
10	Luton & Dunstable University Hospital, Luton	Matthew Johnson
11	North Hampshire Hospital, Basingstoke	Rebecca Saich
12	Queen's Medical Centre, Nottingham	Gordon Moran
13	Royal Devon and Exeter Hospital, Exeter	Tariq Ahmad
14	University Hospital of Wales, Cardiff	Dharmaraj Durai
15	Queen Elizabeth, Kings Lynn	Alan Wiles
16	Epsom General Hospital, Epsom	Pritash Patel
17	James Paget Hospital, Great Yarmouth	Paul Banim
18	Queen Elizabeth Hospital Birmingham	Rachel Cooney
19	West Suffolk Hospital, Bury St Edmunds	Dan Sharpstone
20	Guy's and St Thomas' Hospital, London	Peter Irving
21	Royal Glamorgan Hospital, Glamorgan	James Berrill
22	Ninewells Hospital, Dundee	Craig Mowat
23	St Mary's Hospital, London	Horace Williams
24	Royal Bournemouth Hospital, Bournemouth	Sean Weaver
25	New Cross Hospital, Wolverhampton	Matthew Brookes
26	Blackpool Victoria Hospital, Blackpool	Rhys Butcher
27	Musgrove Park Hospital, Taunton	Emma Wesley
28	Watford General Hospital, Watford	Rakesh Chaudhary
29	Darlington Memorial Hospital, Darlington	Anjan Dhar
30	James Cook University Hospital, Middlesborough	Arvind Ramadas
31	Bedford Hospital, Bedford	Babur Javaid
32	Royal Shrewsbury Hospital, Shrewsbury	Jeffrey Butterworth
33	New Victoria Hospital, Glasgow	Robert Boulton-Jones
34	Wythenshawe Hospital, Manchester	Paul Knight
35	Kingston Hospital, Kingston	Rishi Goel
36	Torbay Hospital, Torquay	Sam Powles
37	St Mark's Hospital, London	Ailsa Hart
38	Royal Victoria Infirmary, Newcastle	Alexander Speight
39	Royal Derby Hospital, Derby	Andrew Cole
40	Worthing Hospital, Worthing	Andy Li

Table S5. Baseline demographics table including biomarker subgroups.

Variable	IBDlo Step up (n=97)	IBDhi Step up (n=96)	IBDlo Top down (n=94)	IBDhi Top down (n=99)	Total (n=386)
Mean age (years)	34.0 (13.3)	34.0 (13.3)	33.3 (13.2)	33.3 (13.2)	33.6 (13.2)
Sex					
Female	48/97 (49%)	40/96 (42%)	43/94 (46%)	48/99 (48%)	179/386 (46%)
Ethnicity					
White	83/97 (86%)	83/93 (89%)	85/96 (89%)	88/99 (89%)	339/385 (88%)
Other	14/97 (14%)	11/96 (11%)	10/93 (11%)	11/99 (11%)	46/385 (12%)
Current smoker	20/97 (21%)	22/96 (23%)	29/94 (31%)	20/99 (20%)	91/386 (24%)
Mean weight (SD; kg)	74.3 (16.5)	75.4 (18.6)	74.0 (17.0)	75.3 (21.3)	74.8 (18.4)
Disease location					
Ileal	34/97 (35%)	26/96 (30%)	36/93 (39%)	29/99 (29%)	128/385 (33%)
Colonic	24/97 (25%)	26/96 (27%)	24/93 (26%)	29/99 (29%)	103/385 (27%)
Ileocolonic	39/97 (40%)	41/96 (43%)	33/93 (35%)	41/99 (41%)	154/385 (40%)
Disease behaviour					
Inflammatory (B1)	84/96 (88%)	77/94 (82%)	79/94 (84%)	90/98 (92%)	330/382 (86%)
Stricturing (B2)	11/96 (11%)	16/94 (17%)	14/94 (15%)	8/98 (8%)	49/382 (13%)
Penetrating (B3)	1/96 (1%)	1/94 (1%)	1/94 (1%)	0/98 (0%)	3/382 (1%)
Mean HBI score (SD)	9.6 (3.1)	10.0 (2.8)	9.8 (2.8)	10.2 (3.0)	9.9 (2.9)
Mean CRP (mg/L; SD)	21 (28)	21 (23)	18 (28)	21 (25)	20 (26.2)
Median CRP (mg/L; IQR)	10 (4-27)	13 (4-19)	9 (6-23)	13 (7-25)	12 (5-23)

Mean Calprotectin (ug/g; SD)	907 (715)	1080 (868)	954 (816)	1110 (990)	1014 (855)
Median Calprotectin (ug/g; IQR)	600 (249 - >1800)	905 (396 - >1800)	714 (383 - 1671)	886 (386 - >1800)	768 (351 - >1800)
Mean SES-CD (SD)	10.2 (5.5)	10.6 (6.4)	10.7 (5.8)	11.2 (7.3)	10.7 (6.27)
Median SES-CD (IQR)	9 (7 - 13)	9 (7 - 14)	10 (6 - 13)	10 (7 - 15)	9 (7 - 13)
Steroid course prior to enrolment	18/96 (19%)	22/96 (23%)	15/94 (16%)	15/99 (15%)	70/385 (18%)
Mean time from diagnosis to enrolment (days; SD)	26.7 (33.3)	35.9 (45.5)	19.3 (26.4)	28.2 (40.0)	27.6 (37.4)
Median time from diagnosis to enrolment (days; min-max)	13.0 (0 - 138)	17.5 (0 - 191)	10.0 (0 - 168)	8.0 (0 - 165)	11.5 (0-191)
Randomisation strata					
Biomarker status					
IBDhi	*0/97 (0%)*	*96/96 (100%)*	*0/94 (0%)*	*99/99 (100%)*	*195/386 (51%)*
IBDlo	*97/97 (100%)*	*0/96 (0%)*	*94/94 (100%)*	*0/99 (0%)*	*191/386 (49%)*
Disease location					
Colonic	*26/97 (27%)*	*25/96 (26%)*	*23/94 (24%)*	*27/99 (27%)*	*101/386 (26%)*
Other	*71/97 (73%)*	*71/96 (74%)*	*71/94 (76%)*	*72/99 (73%)*	*285/386 (74%)*
Endoscopic inflammation					
Mild	*9/97 (9%)*	*5/96 (5%)*	*7/94 (7%)*	*6/99 (6%)*	*27/386 (7%)*
Moderate	*66/97 (68%)*	*70/96 (73%)*	*66/94 (70%)*	*70/99 (71%)*	*272/386 (70%)*
Severe	*22/97 (23%)*	*21/96 (22%)*	*21/94 (22%)*	*23/99 (23%)*	*87/386 (23%)*

Data are n (%) unless otherwise stated. HBI=Harvey Bradshaw Index. CRP=C-reactive protein. SES-CD=Simplex Endoscopic Score for Crohn's Disease. There were three randomisation strata and these data are listed as they were entered on Sealed Envelope by local investigators at the time of randomisation. The variables otherwise listed are as per entries from PROFILE trial case report forms. We note minor discrepancies in data entered for disease location.

Table S6. Patients with surgical operations during PROFILE.

Treatment arm	Indication for surgery	Biomarker subgroup	Luminal disease behaviour at baseline	Time from randomisation to surgery (days)
**Abdominal surgeries**				
Accelerated step-up	Penetrating disease	IBDlo	Stricturing disease (B2)	27
Accelerated step-up	Penetrating disease	IBDlo	Inflammatory disease (B1)	388
Accelerated step-up	Penetrating disease	IBDlo	Inflammatory disease (B1)	240
Accelerated step-up	Penetrating disease	IBDhi	Penetrating disease (B3)	173
Accelerated step-up	Penetrating disease	IBDhi	Penetrating disease (B3)	177
Accelerated step-up	Stricturing disease	IBDlo	Stricturing disease (B2)	118
Accelerated step-up	Stricturing disease	IBDhi	Inflammatory disease (B1)	209
Accelerated step-up	Stricturing disease	IBDlo	Inflammatory disease (B1)	137
Accelerated step-up	Penetrating disease	IBDlo	Inflammatory disease (B1)	236
Accelerated step-up	Penetrating disease	IBDlo	Inflammatory disease (B1)	89
Top-down	Gallstone ileus	IBDhi	Inflammatory disease (B1)	161
**Perianal surgeries**				
Accelerated step-up	Perianal disease	IBDlo	Inflammatory disease (B1)	43
Top-down	Perianal disease	IBDhi	Inflammatory disease (B1)	329

Table S7. Hospital admissions and surgeries during PROFILE.

	Accelerated step-up (n=193)	Top-down (n=193)
Absolute number of hospitalisations and surgeries (%)	25 (13%)	15 (8%)
Mean (SD) of hospitalisations and surgeries	0.22 (0.64)	0.10 (0.40)

The total number of hospital admissions for a participant in PROFILE could be 0, 1, 2, or 3 rather than being a binary endpoint of requiring hospital admission or not. Therefore, the mean number of hospitalisations and surgeries is likely to be more meaningful for determining a difference between the two treatment strategies used in PROFILE. However, for completeness, the absolute number of hospital admissions and surgeries have been provided.

Table S8. Adverse events in PROFILE including biomarker subgroups.

A

**Group of AEs**	**IBDlo Step up (n=97)**	**IBDhi Step up (n=96)**	**IBDlo Top down (n=94)**	**IBDhi Top down (n=99)**
Adverse event due to Crohn's disease	122, 69 (71%)	103, 63 (67%)	10, 10 (11%)	20, 16 (16%)
Infection	6,4 (4%)	14, 8 (8%)	9, 8 (9%)	14, 8 (8%)
Thiopurine intolerance	35, 26 (27%)	24, 22 (23%)	40, 29 (31%)	47, 33 (33%)
Methotrexate intolerance	2, 2 (2%)	7, 2 (2%)	5, 4 (4%)	3, 2 (2%)
Infliximab intolerance	0	0	6, 6 (6%)	2, 2 (2%)
Malignancy	0	0	0	0
Other	2, 2 (2%)	0	5, 4 (4%)	7, 4 (4%)

B

**Group of SAEs**	**IBDlo Step up (n=97)**	**IBDhi Step up (n=96)**	**IBDlo Top down (n=94)**	**IBDhi Top down (n=99)**
Hospitalisation for flare of Crohn's disease	10, 8 (8%)	5, 4 (4%)	3, 3 (3%)	0
Surgery for disease complication	8, 8 (8%)	3,2 (2%)	0	2, 2 (2%)
Abdominal surgery	7, 7 (7%)	3, 2 (2%)	0	1, 1 (1%)
Perianal surgery	1, 1 (1%)	0	0	1, 1 (1%)
Medication related	0	1, 1 (1%)	1, 1 (1%)	0
Serious infection	1, 1 (1%)	7, 3 (3%)	3, 3 (3%)	0
Malignancy	0	0	0	0
Death	0	0	0	0
Other	4,3 (3%)	3, 3 (3%)	2, 1 (1%)	4,3 (3%)

Adverse events (AEs) and serious adverse events (SAEs) presented as number of events, number of patients (percentage).

Figure S1. Incidence and relative risk of adverse events.

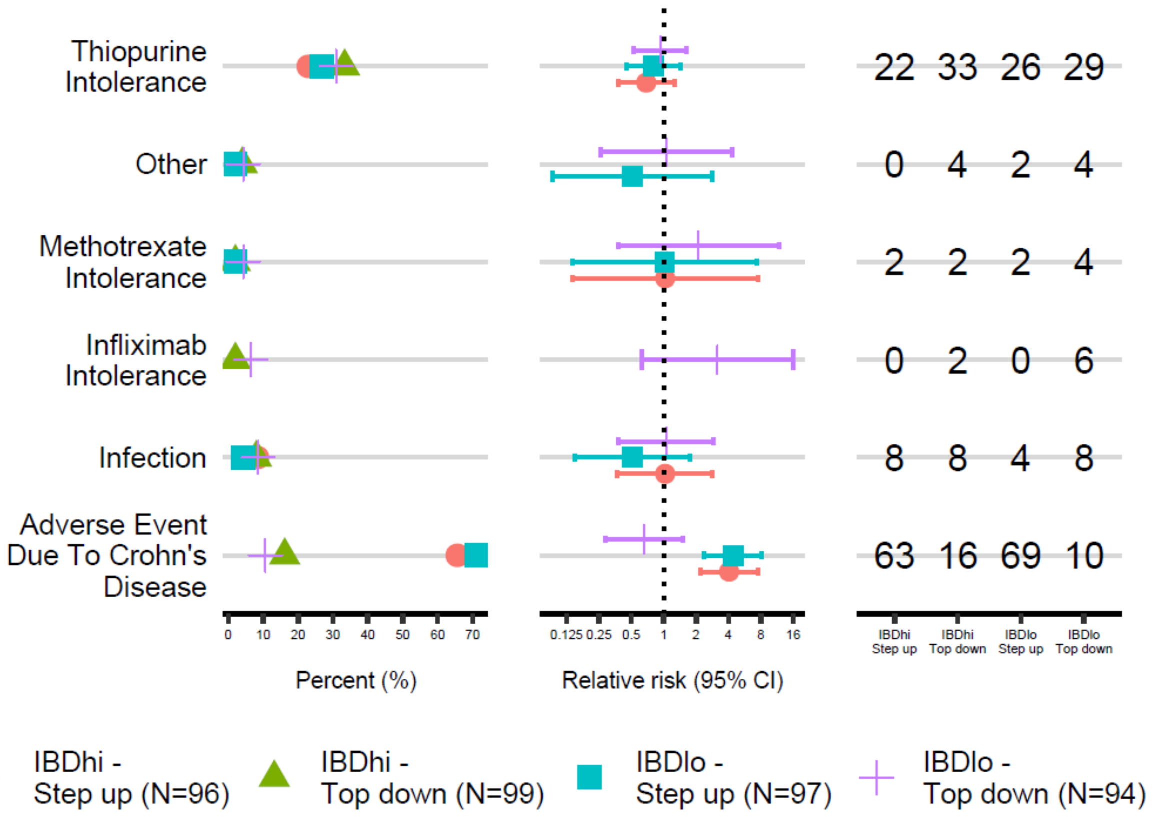

(A) Showing incidence (%) of adverse events in each treatment subgroup. (B) Showing relative risk of adverse events given for each treatment subgroup compared to the "IBDhi top-down" group. Adverse event due to Crohn's disease indicates a flare of Crohn's disease.

Figure S2. Endoscopic response (≥50% improvement in SES-CD score) at week 48.

(A) For treatment groups.

(B) For biomarker-treatment subgroups.

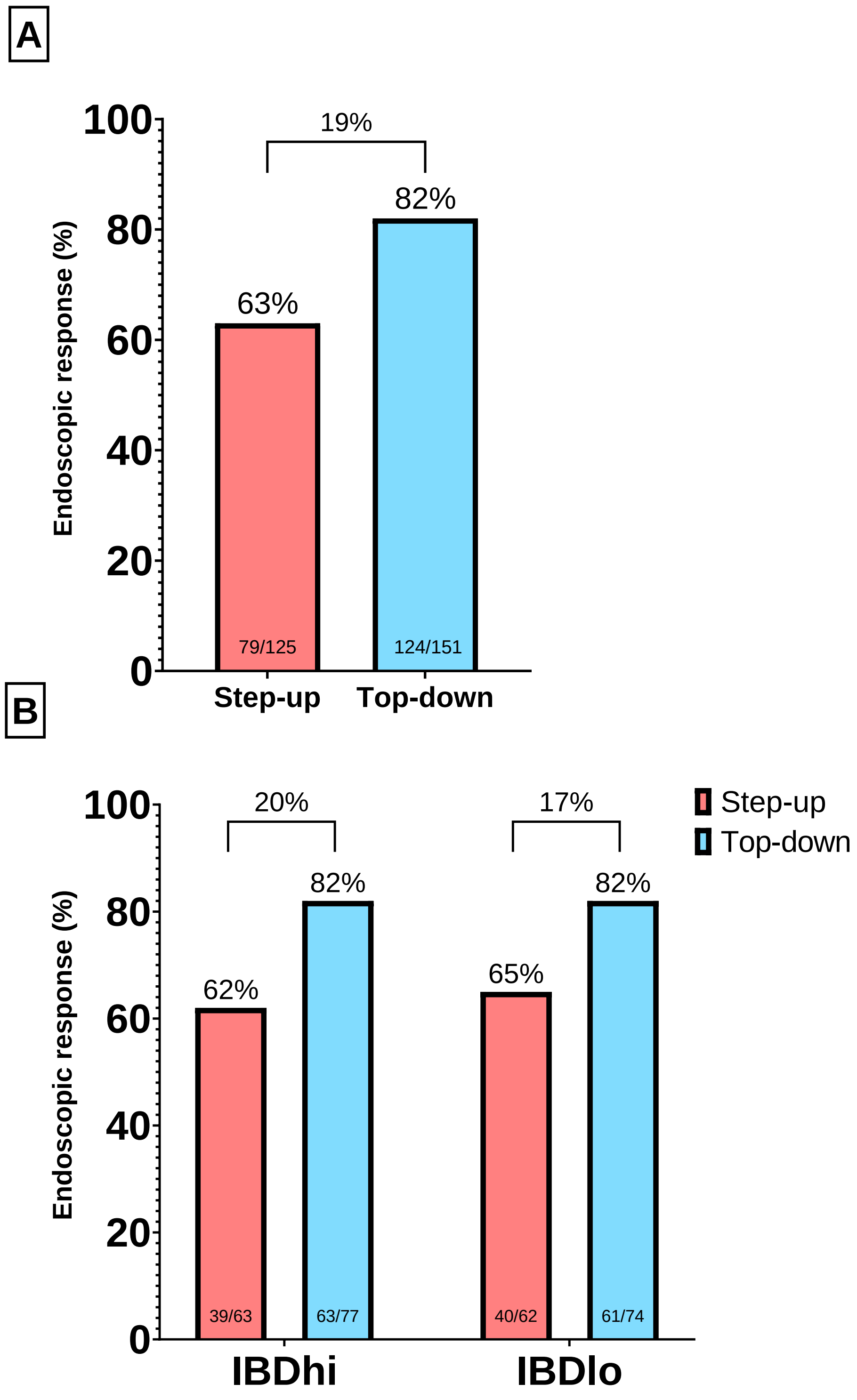

Endoscopic response was based on local investigator scores for both end-of-trial ileo-colonoscopy and baseline ileo-colonoscopy.

Figure S3. Deep endoscopic remission (total SES-CD score=0) at week 48.

(A) For treatment groups.

(B) For biomarker-treatment subgroups.

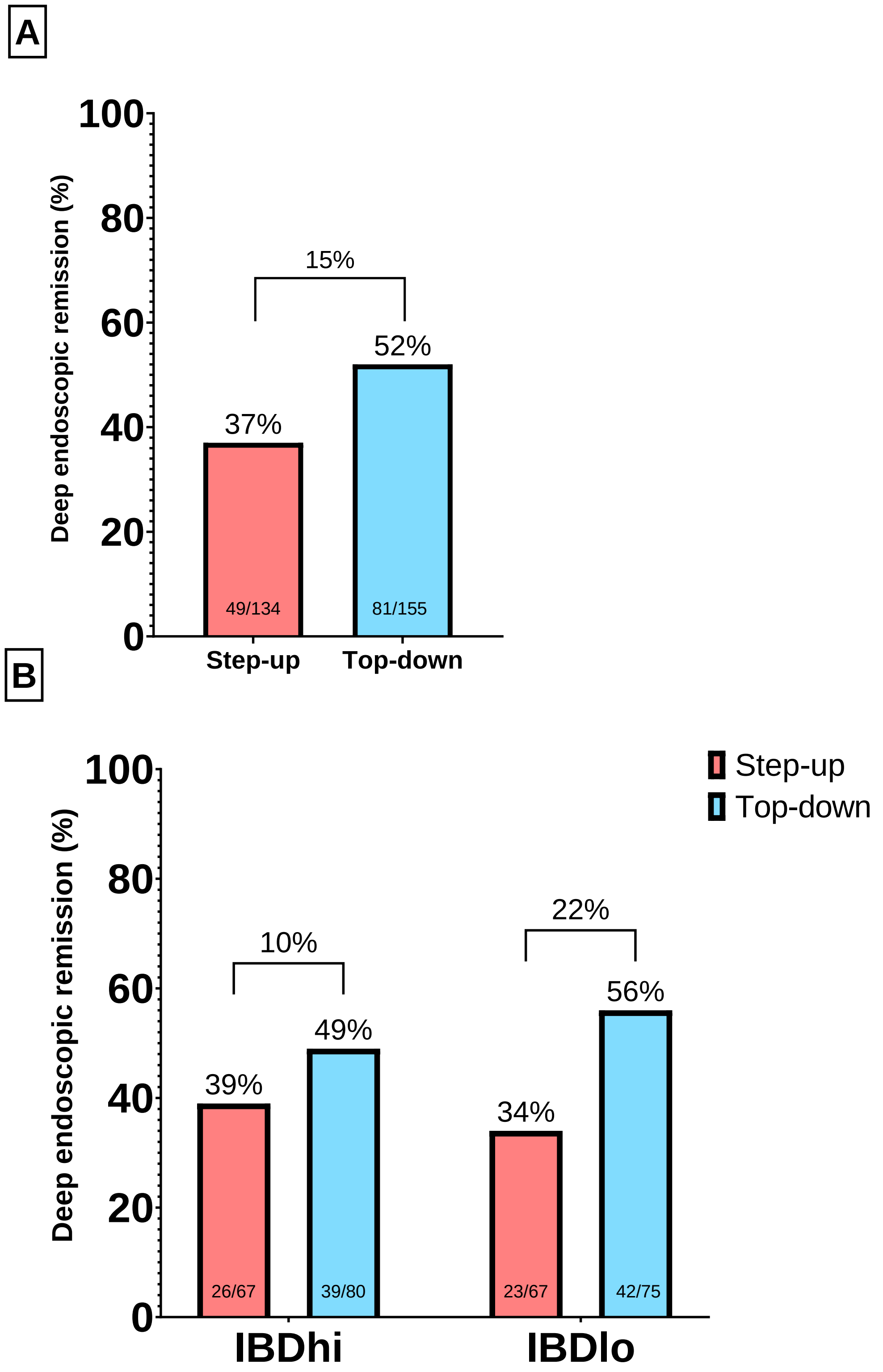

Combination of centrally-read scores when end-of-trial ileo-colonoscopy was video-recorded and local investigator scores for the remainder.

Figure S4. Composite of clinical remission (HBI score <5) and endoscopic remission (absence of ulcers) at week 48.

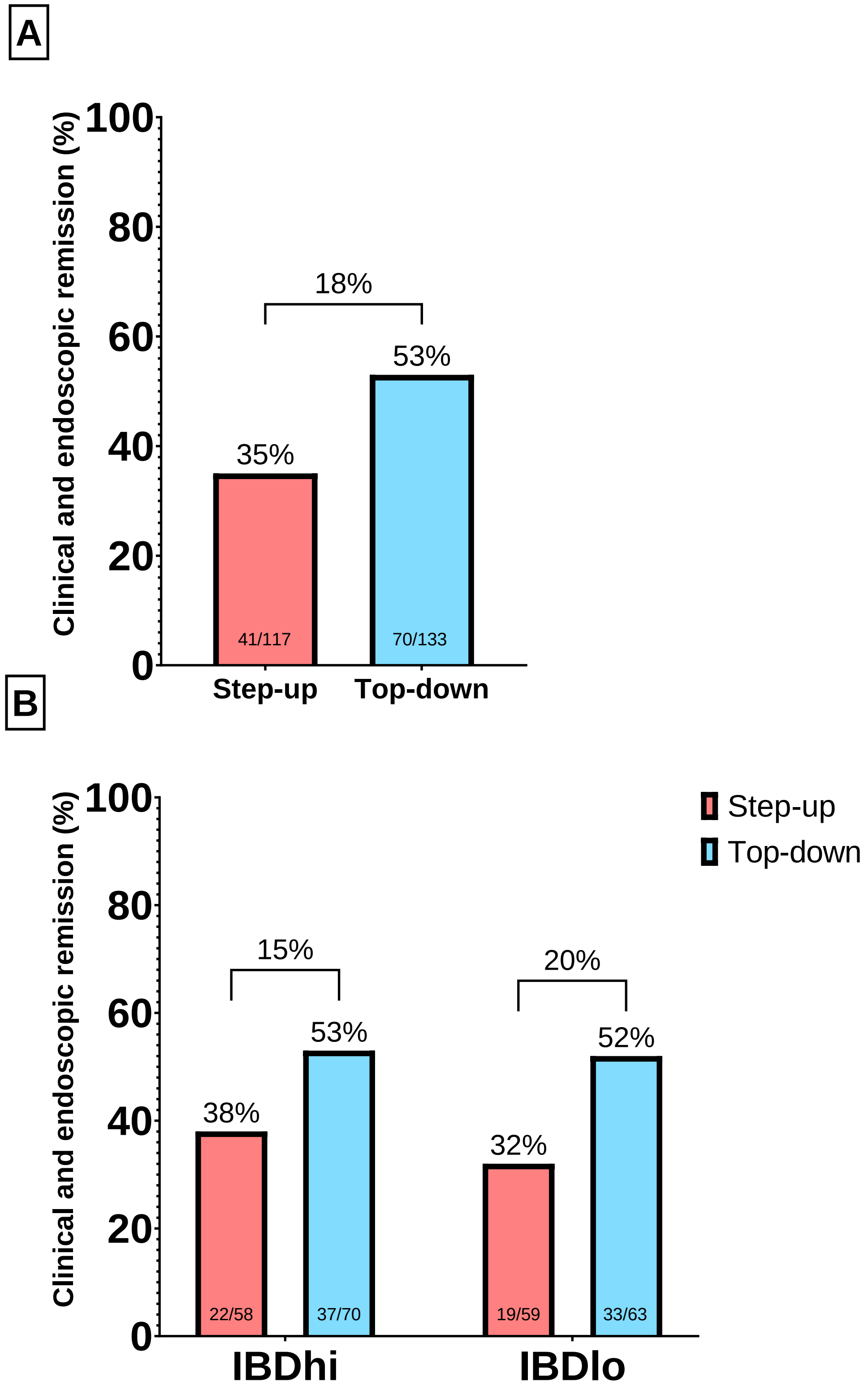

Combination of centrally-read scores when end-of-trial ileo-colonoscopy was video-recorded and local investigator scores for the remainder.

Figure S5. Clinical remission alone (HBI score <5) at week 48.

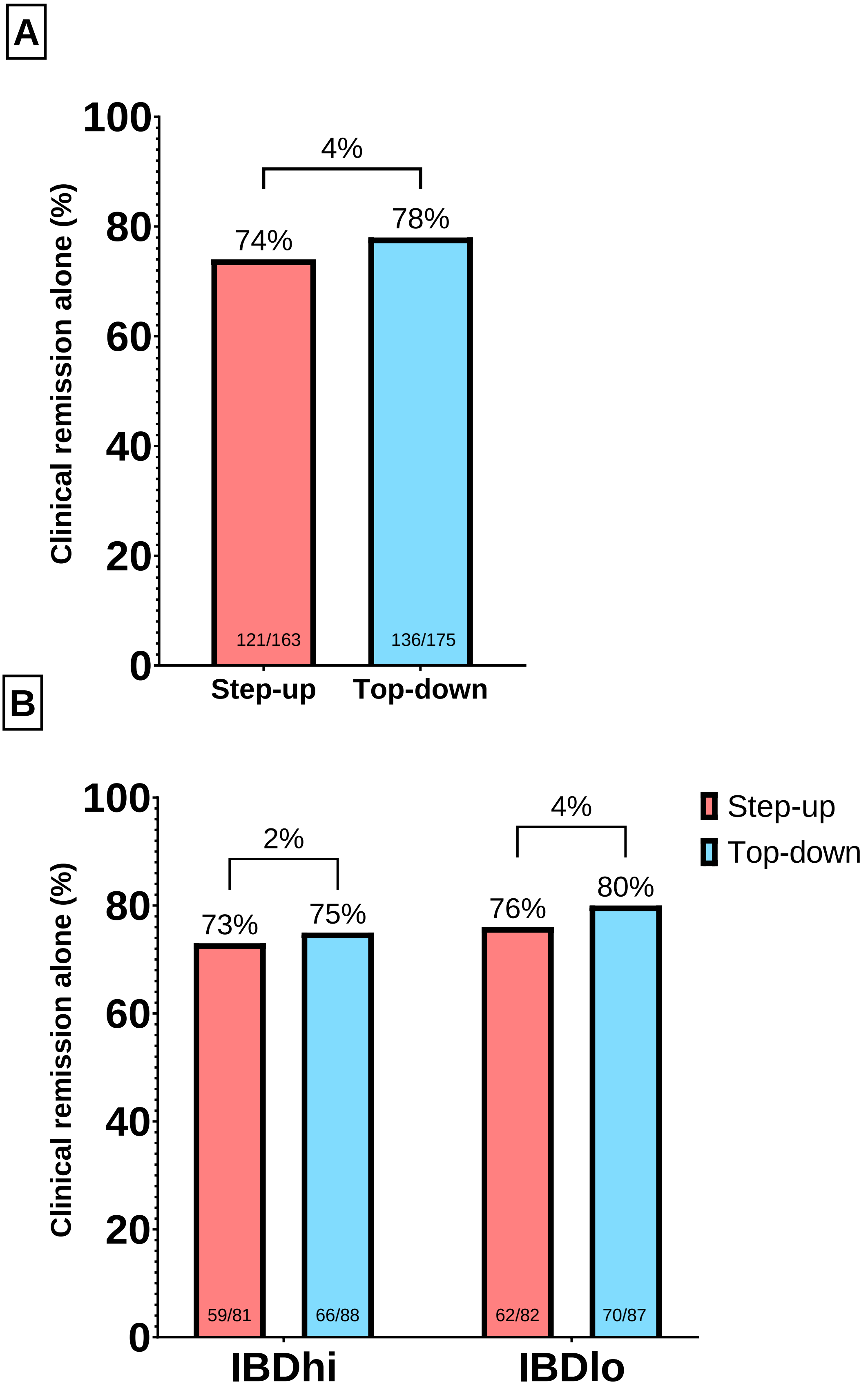

Figure S6. Composite of clinical remission (HBI score <5) and biochemical remission (CRP ≤ULN and calprotectin <200ug/g) at week 48.

A

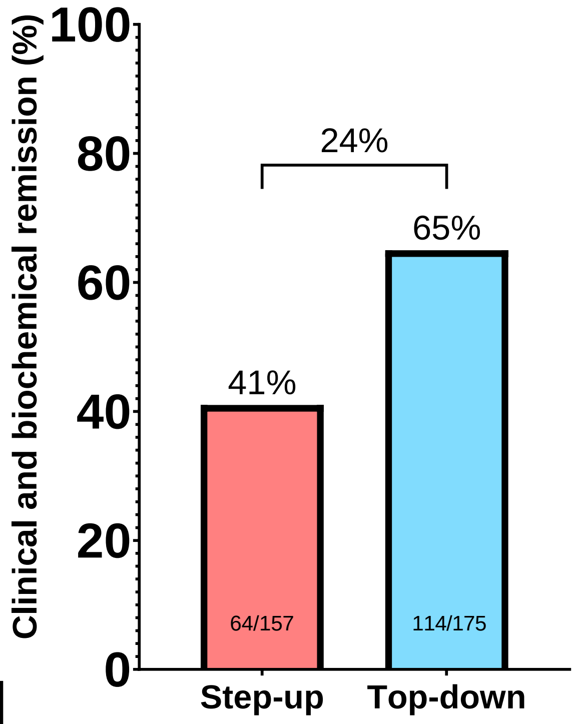

B

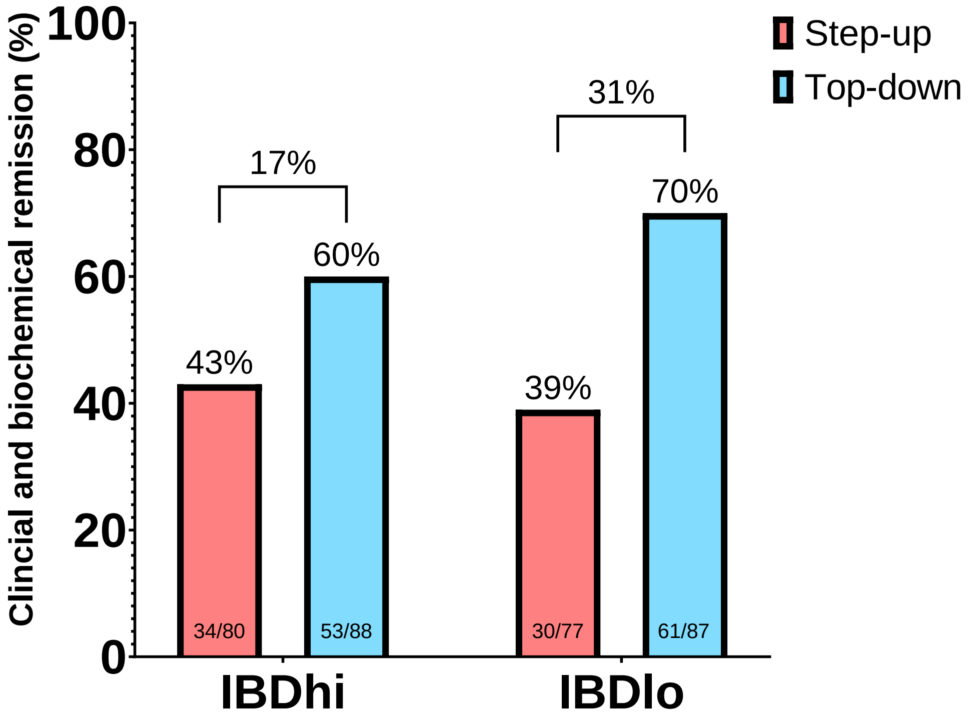

Figure S7. Median CRP.

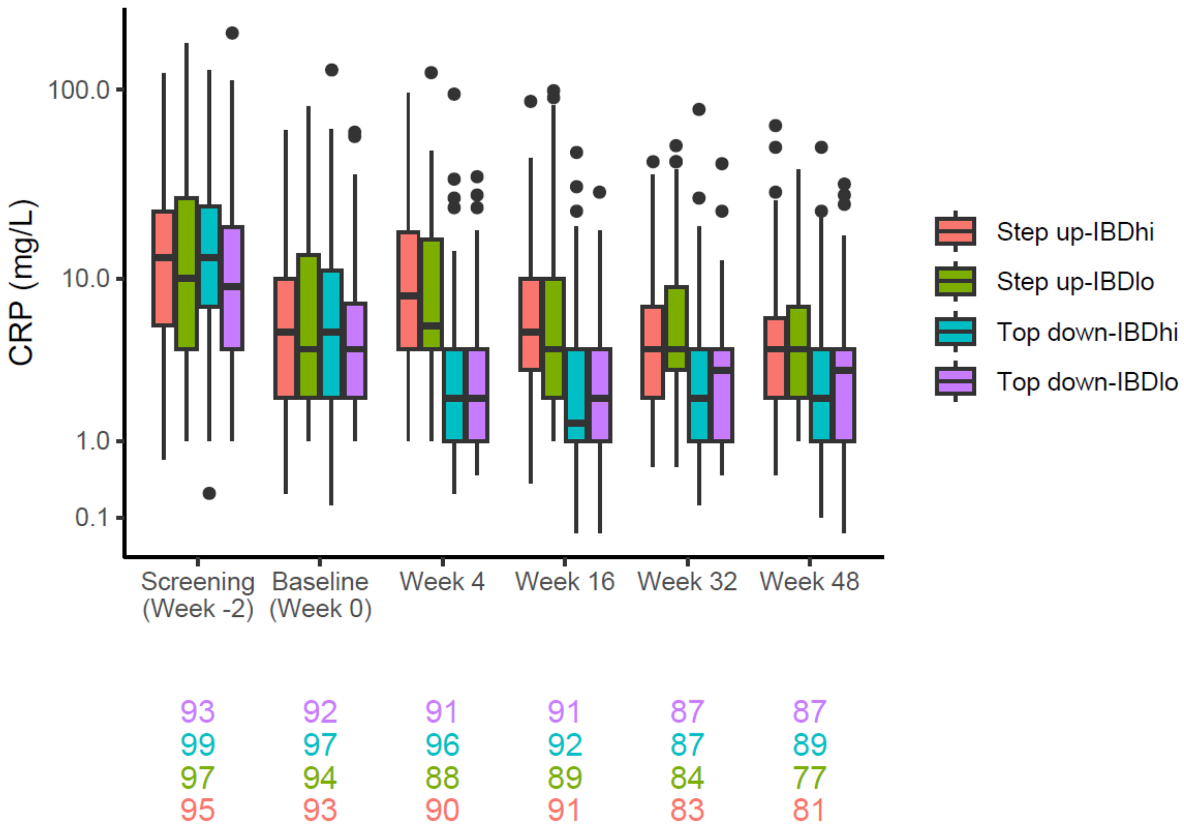

Figure S8. Median faecal calprotectin.

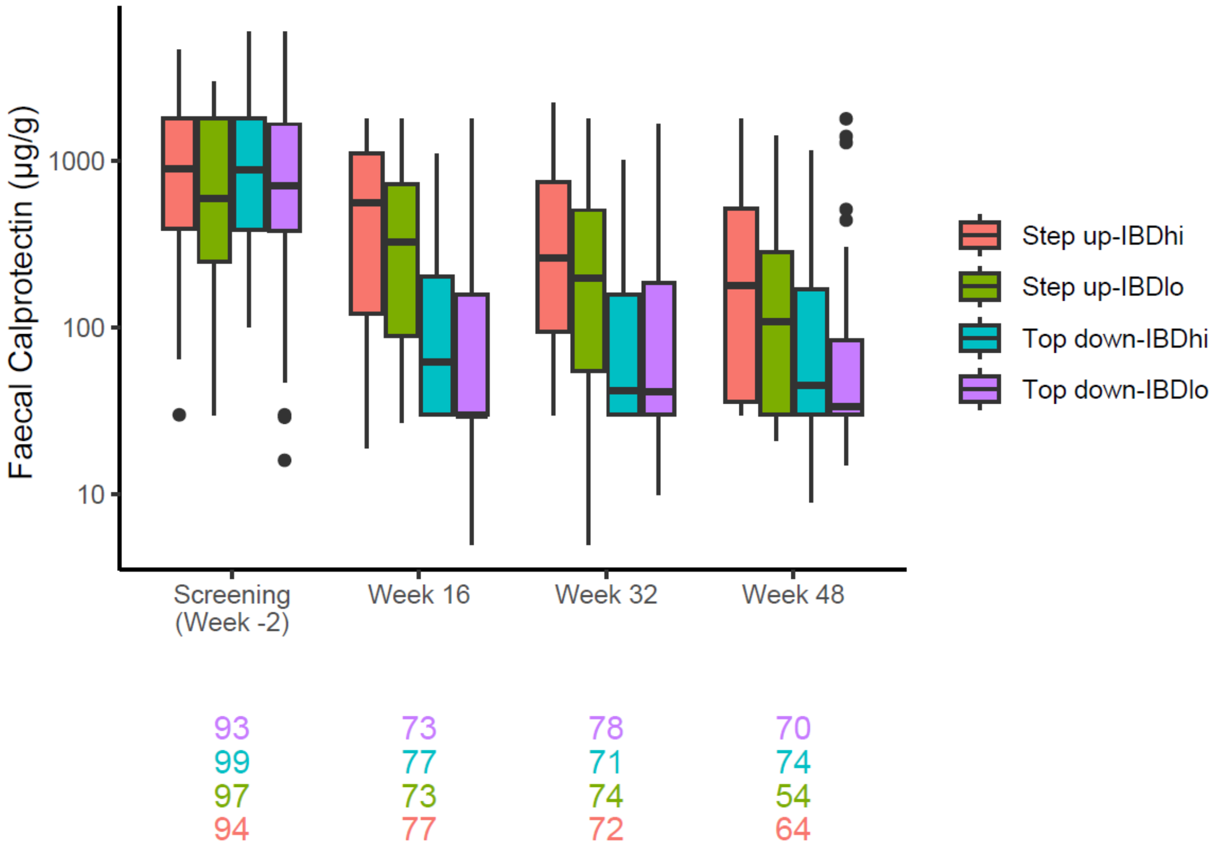

Figure S9. Biochemical remission (CRP ≤ULN and calprotectin <200ug/g) at week 48.

(A) For treatment groups.

(B) For biomarker-treatment subgroups.

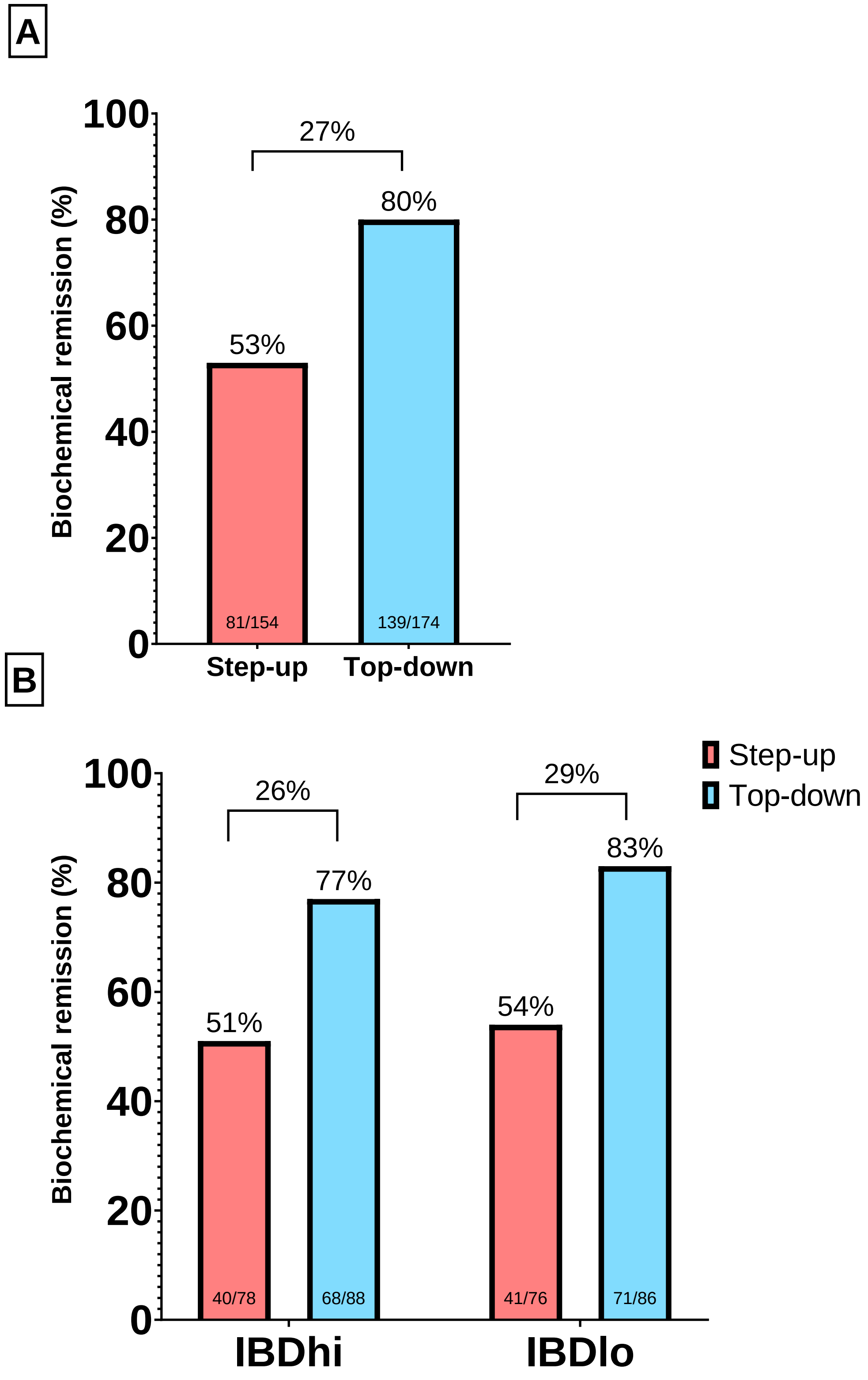

Figure S10. Forest plot of the association between clinical variables and the primary endpoint independent of treatment or biomarker subgroup.

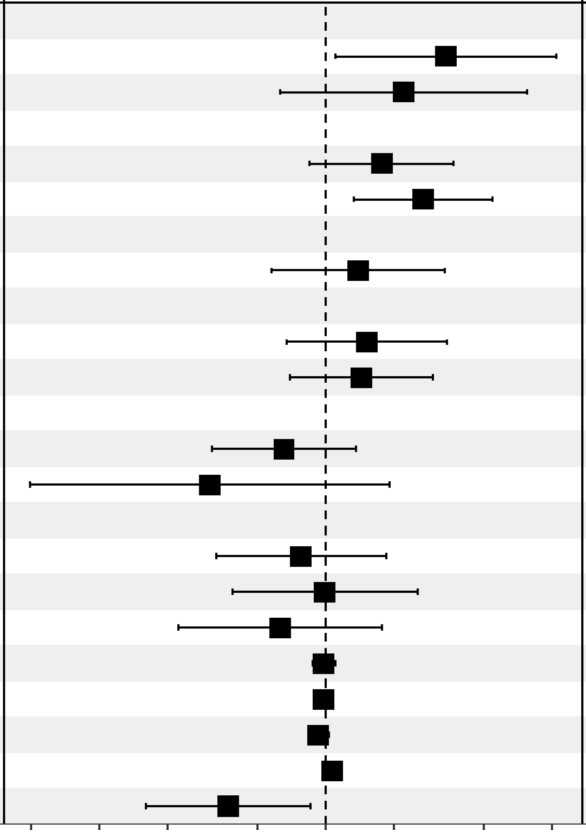

Variable		N	Odds ratio	p
**Endoscopic inflammation**	Mild	27/386		
	Moderate	272/386	3.39 (1.11, 10.38)	0.033
	Severe	87/386	2.21 (0.63, 7.75)	0.215
**Disease location**	Ileal	128/385		
	Colonic	103/385	1.76 (0.85, 3.68)	0.131
	Ileocolonic	154/385	2.69 (1.33, 5.42)	0.006
**Disease behaviour**	B1: Inflammatory	330/382		
	B2 or B3: Stricturing or penetrating	52/382	1.39 (0.57, 3.35)	0.469
**Smoking**	Current	91/384		
	Former	101/384	1.52 (0.67, 3.43)	0.318
	Never	192/384	1.43 (0.69, 2.96)	0.334
**Age**	<40	280/386		
	40−64	94/386	0.65 (0.32, 1.35)	0.253
	65+	12/386	0.31 (0.05, 1.91)	0.207
**BMI**	<20	65/385		
	20−24	143/385	0.78 (0.33, 1.84)	0.568
	25−29	105/385	0.99 (0.39, 2.53)	0.985
	30+	72/385	0.63 (0.22, 1.78)	0.384
**CRP (per 10 mg/L change)**			0.98 (0.87, 1.10)	0.728
**Calprotectin (per 100 µg/g change)**			0.97 (0.94, 1.01)	0.122
**HBI (per unit change)**			0.93 (0.84, 1.03)	0.154
**Time from endoscopy to screening (per week)**			1.07 (1.00, 1.13)	0.035
**Prior corticosteroids**		70/386	0.37 (0.16, 0.86)	0.021

Data presented as conditional odds ratios when adjusting for baseline clinical covariates in the primary analysis.

Figure S11. Disease flares requiring treatment escalation.

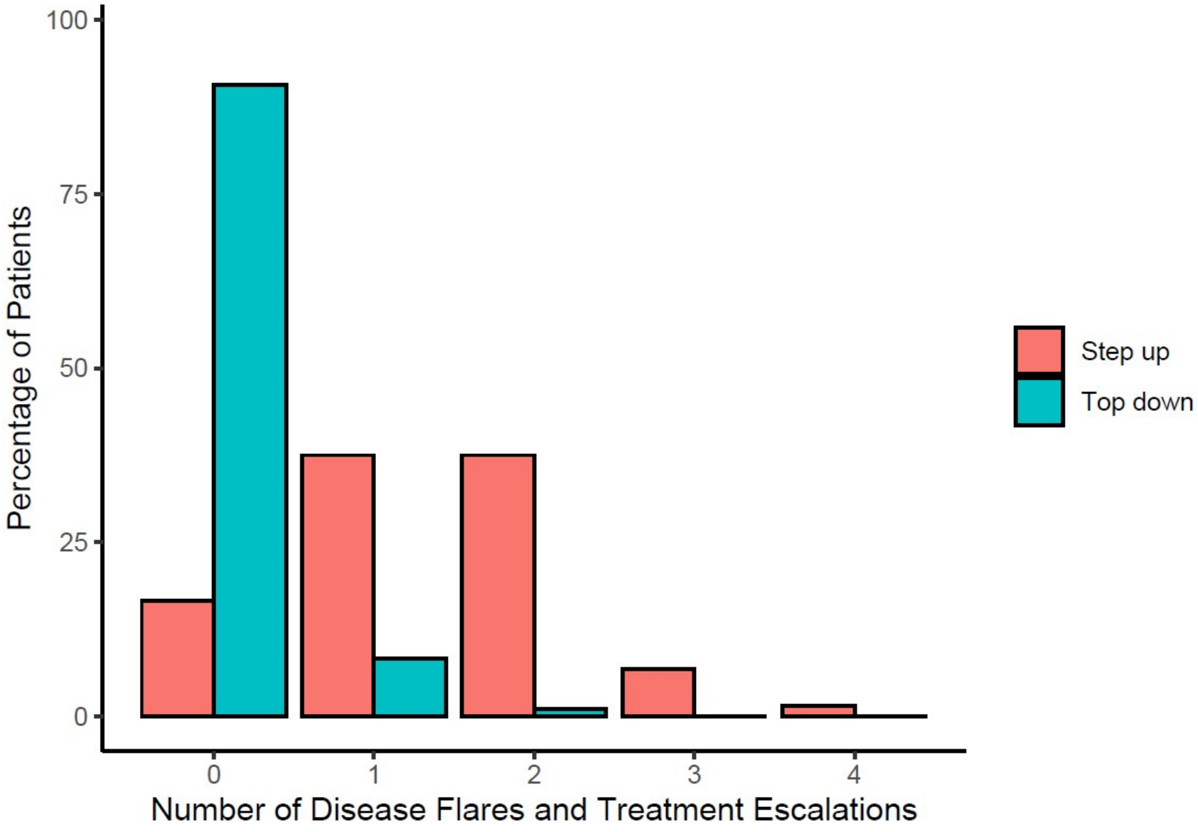

**Number of flares requiring escalation**	**0**	**1**	**2**	**3**	**4**
Accelerated step-up	32/192	72/192	72/192	13/192	3/192
Top-down	175/193	16/193	2/193	0/193	0/193

Figure S12. Additional course(s) of steroid after initial induction course.

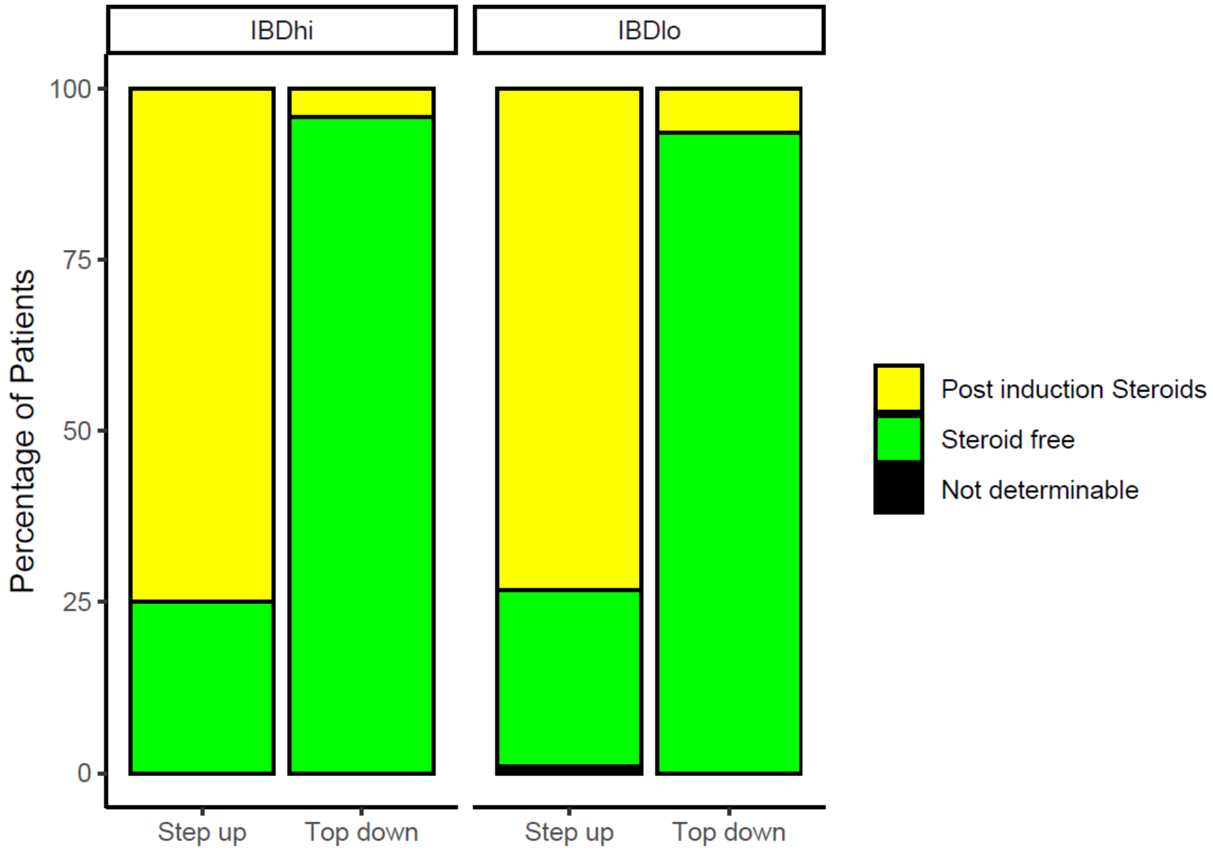

	Accelerated step-up - IBDhi	Top-down - IBDhi	Accelerated step-up – IBDlo	Top-down - IBDlo
Required post-induction steroids	72/96 (75%)	4/99 (4%)	71/97 (73%)	6/94 (6%)
Steroid-free after induction	24/96 (25%)	95/99 (96%)	25/97 (26%)	88/94 (94%)
Not determinable (data not available)	0	0	1/97 (1%)	0

Figure S13. Mean quality-of-life numerical score (using IBD-Q).

(A) For treatment groups.

(B) For biomarker-treatment subgroups.

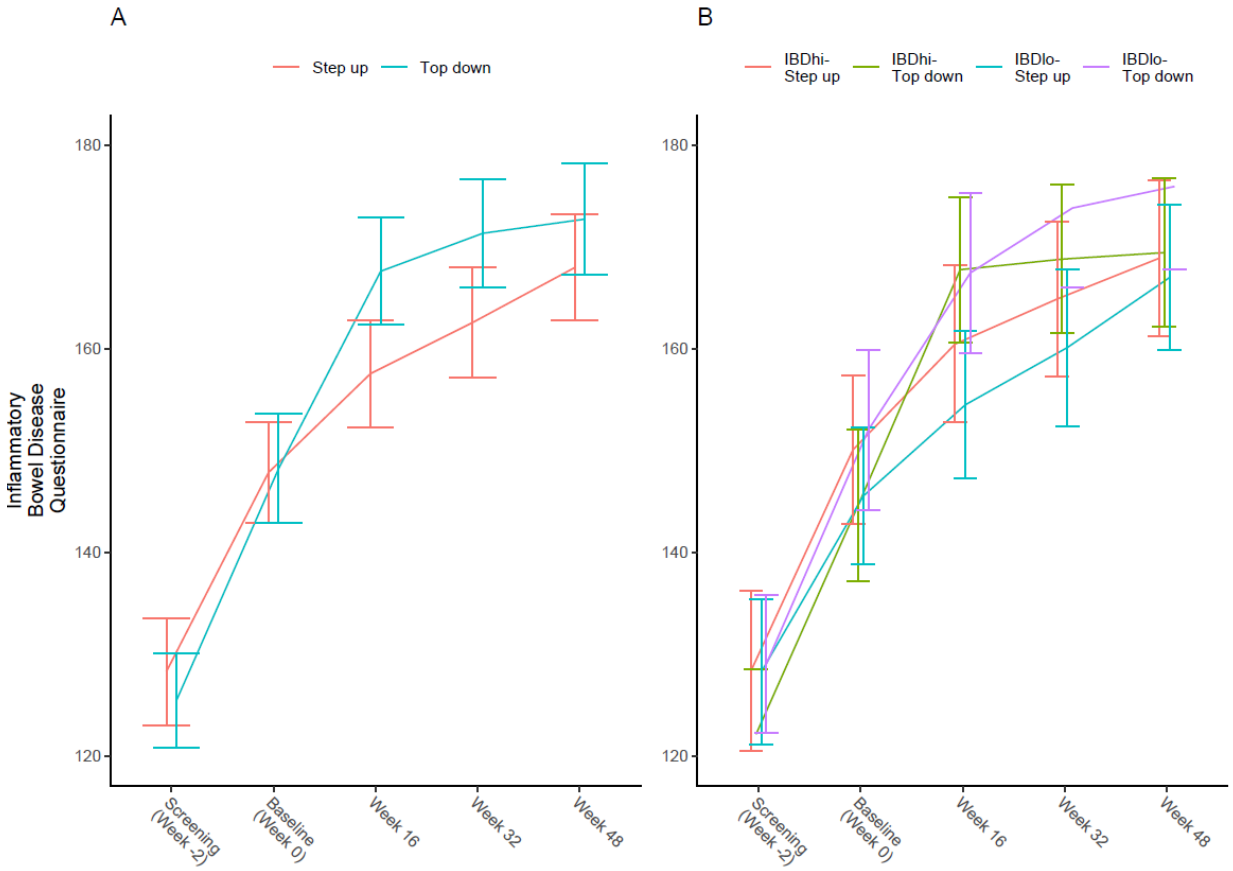

Total IBD-Q numerical score was calculated at each of the above visits, with minimum possible score on this questionnaire of 32 and maximum possible score of 224. Higher scores are associated with better quality-of-life for patients. (A) Higher quality of life numerical scores in "top-down" compared to "accelerated step-up" absolute difference=8.54 (95% CI=3.5 to 13.6, p<0.001). (B) No significant effect on quality of life numerical score based on biomarker-treatment subgroup (absolute difference=1.42, 95% CI=-8.76 to +11.60, p<0.784).

Figure S14. Quality-of-life remission (IBD-Q >170).

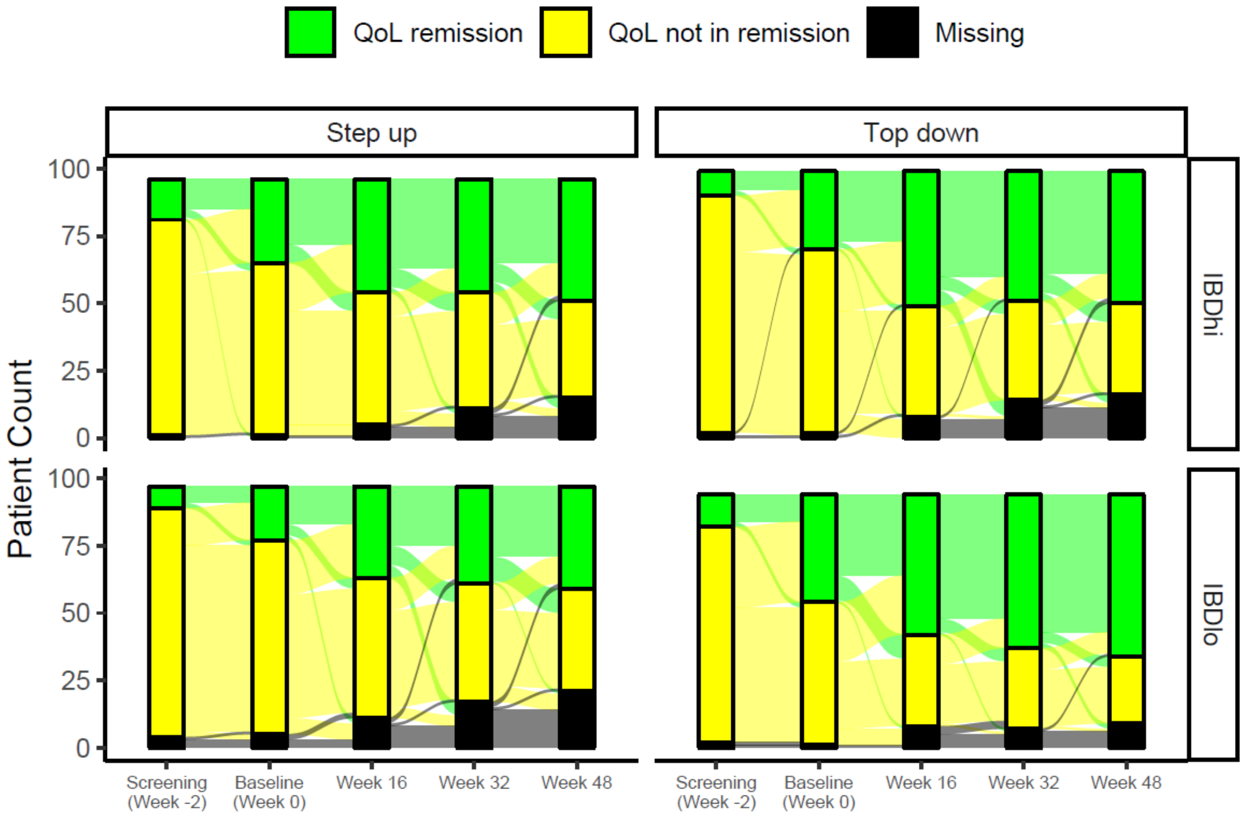

Figure S15. CRP and calprotectin for those who had end of trial ileo-colonoscopy versus those who did not have end of trial ileo-colonoscopy.

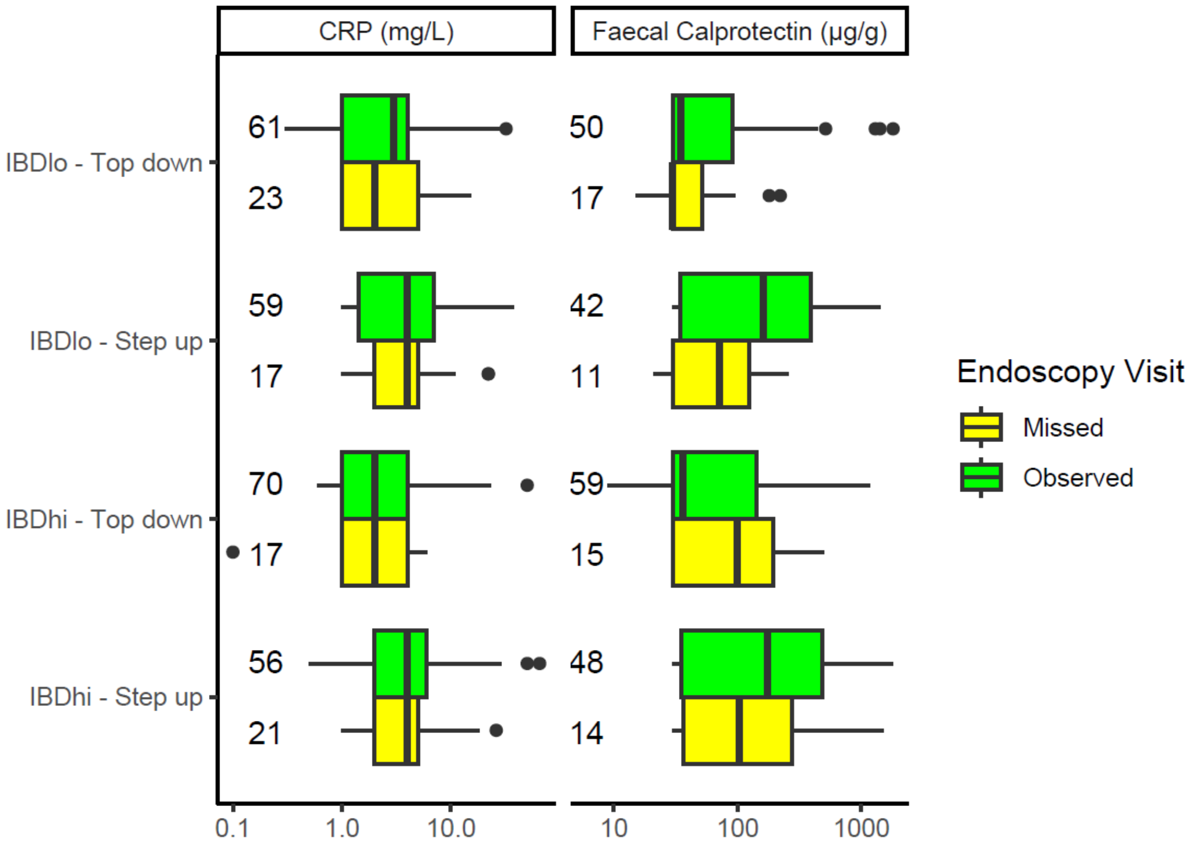

Figure S16. Endoscopic remission (absence of ulcers) at week 48 using centrally-read videos only.

(A) For treatment groups.

(B) For biomarker-treatment subgroups.

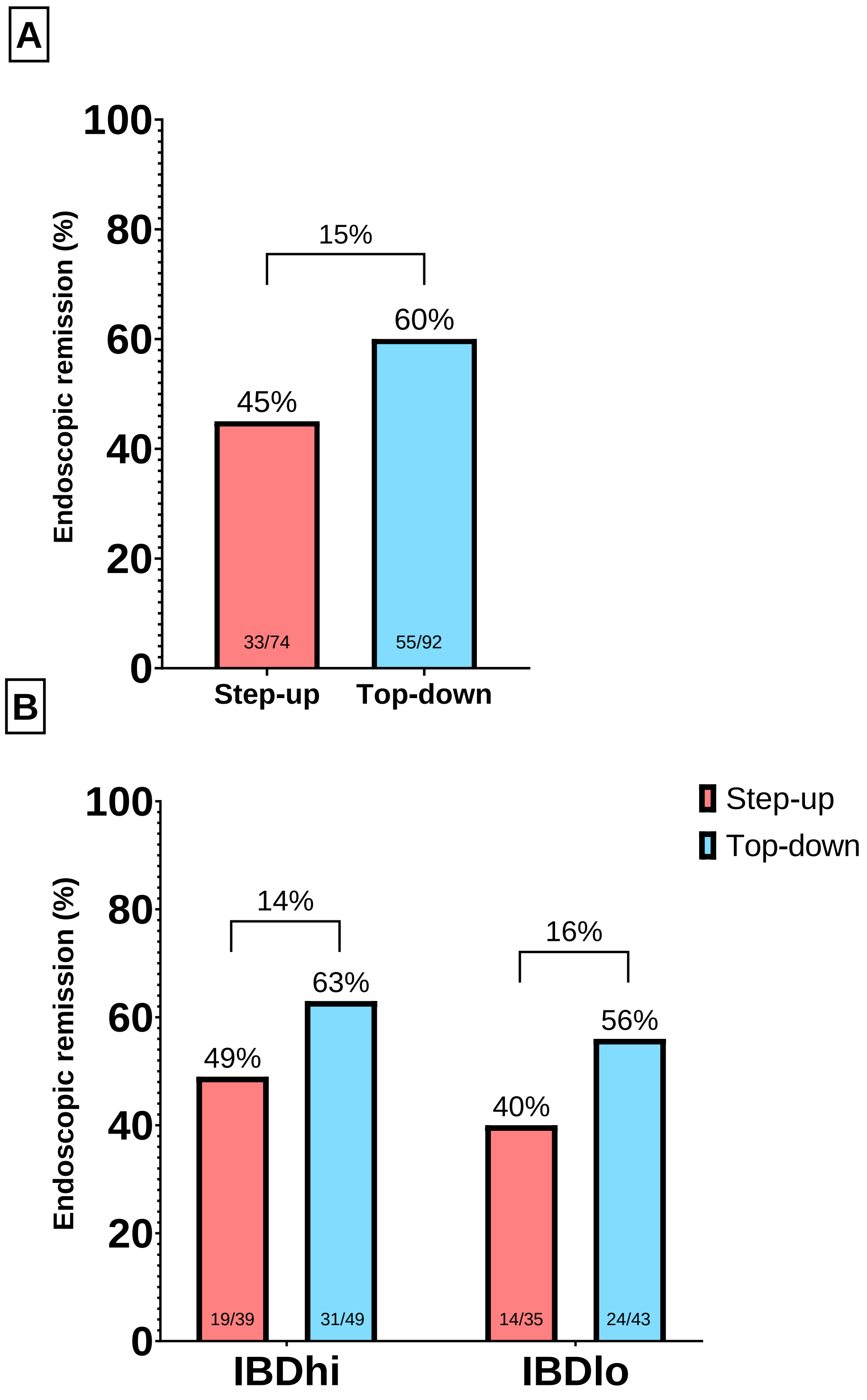

Only centrally-read scores from end-of-trial ileo-colonoscopies were included in this analysis.
