# Supplementary material for: A biomarker-stratified comparison of top-down versus accelerated step-up treatment strategies for patients with newly diagnosed Crohn's disease (PROFILE): a multicentre, open-label randomised controlled trial
Source: Lancet Gastroenterol Hepatol. 2024 Feb 22;9(5):415–27. doi: 10.1016/S2468-1253(24)00034-7 (PMC11001594; doi:10.1016/S2468-1253(24)00034-7)
Supplement: Supplementary appendix 2 [file mmc2.pdf]

THE LANCET
Gastroenterology & Hepatology

Supplementary appendix 2

This appendix formed part of the original submission and has been peer reviewed. We post it as supplied by the authors.

Supplement to: Noor NM, Lee JC, Bond S, et al. A biomarker-stratified comparison of top-down versus accelerated step-up treatment strategies for patients with newly diagnosed Crohn's disease (PROFILE): a multicentre, open-label randomised controlled trial. *Lancet Gastroenterol Hepatol* 2024; published online Feb 22. https://doi.org/10.1016/S2468-1253(24)00034-7.

Contents

PROFILE Trial Protocol. 2

PROFILE Trial Statistical Analysis Plan. 63

PROFILE Trial Protocol.

Trial Title: **PR**edicting **O**utcomes **F**or Crohn's d**I**sease using a mo**L**ecular biomark**E**r (PROFILE) trial

Protocol Version: 6.0 – 10.05.2023

Chief Investigator: Professor Miles Parkes

CI Address: Department of Gastroenterology, Box 201A, Cambridge University Hospital, Cambridge. CB2 0QQ

Telephone: + 44 (0) 1233 245 151

Co-ordinating Centre: Cambridge Clinical Trials Unit (CCTU)

Coton House, Level 6, Box 401, Addenbrooke's Hospital, Hills Road, Cambridge. CB2 0QQ.

Telephone: +44 (0) 1223 254 666

Trial Sponsor: Cambridge University Hospitals NHS Foundation Trust and University of Cambridge

SAE Reporting: Cambridge Clinical Trials Unit add-tr.profile@nhs.net

Funder: Wellcome Trust (200448/Z/16/Z)

Protocol Signatures:

I give my approval for the attached protocol entitled Predicting Outcomes for Crohn's disease using a molecule biomarker (PROFILE) trial V6.0.

Chief Investigator

Name: Professor Miles Parkes

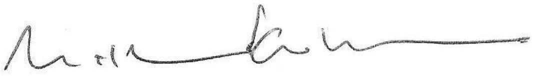

Signature:

Date: 10.05.2023

Site Signatures

I have read the attached protocol entitled "Predicting Outcomes for Crohn's disease using a molecule biomarker (PROFILE) trial" V6.0 and agree to abide by all provisions set forth therein.

I agree to comply with the conditions and principles of Good Clinical Practice as outlined in the European Clinical Trials Directives 2001/20/EC and the GCP Directive 2005/28/EC.

I agree to ensure that the confidential information contained in this document will not be used for any other purpose other than the evaluation or conduct of the clinical investigation without the prior written consent of the Sponsor.

Principal Investigator

Name:

Signature: ____

Date: ______________________________

Once signed please, please return a copy of this page only to the Trial coordinating office.

1 Trial Management Committee (TMG) and Protocol Contributors

Chief Investigator

Professor Miles Parkes

Department of Medicine

University of Cambridge School of Clinical Medicine Cambridge. CB2 0QQ.

Telephone: + 44 (0) 1233 245 151

Email: miles.parkes1@nhs.net

Co-Investigators

Professor Kenneth Smith (Addenbrooke's Hospital, Cambridge, United Kingdom).

Dr James Lee (Addenbrooke's Hospital, Cambridge, United Kingdom).

Professor Eoin McKinney (Addenbrooke's Hospital, Cambridge, United Kingdom).

Dr Nurulamin Noor (Addenbrooke's Hospital, Cambridge, United Kingdom).

Chief Technical Officers

Dr Paul Lyons (University of Cambridge, Cambridge, United Kingdom).

Dr Daniele Biasci (University of Cambridge, Cambridge, United Kingdom).

Trial Co-ordinator

Mr Francis Dowling (Cambridge Clinical Trials Unit, Addenbrooke's Hospital, Cambridge, United Kingdom).

Suhaylah Bhatti (Cambridge Clinical Trials Unit, Addenbrooke's Hospital, Cambridge, United Kingdom).

Trial Statistician

Dr Simon Bond (Cambridge Clinical Trials Unit, Addenbrooke's Hospital, Cambridge, United Kingdom).

Trial Pharmacist

Mrs Robyn Staples (Addenbrooke's Hospital, Cambridge, United Kingdom).

Data Monitoring Committee

Independent chair – Professor Vipul Jairath (University Hospital, Ontario, Canada).

Independent expert statistician – Professor Robert Gray (Harvard T.H. Chan School of Public Health, Boston, Massachusetts, United States of America).

Independent expert member – Professor Severine Vermeire (Catholic University of Leuven, Leuven, Belgium).

Trial Steering Committee

Independent Chair - Professor Geert D'Haens (Amsterdam Medical Centre, Amsterdam, Netherlands).

Independent statistician – Graham Wetherill.

Independent Expert Member - Professor Gijs Van Den Brink (Roche, Basel,

Switzerland).

Table of Contents

Protocol Signatures: 3

1 Trial Management Committee (TMG) and Protocol Contributors 5

Table of Contents 7

2 Abbreviations 10

3 Trial Synopsis 12

4 Trial Flow Chart 20

5 Introduction 21

5.1 Background21

5.2 Biomarker - Preliminary work 22

5.3 Biomarker – Data development of whole blood classifier 22

6 Rationale for Trial 24

7 Trial Design 24

7.1 Statement of design 24

7.2 Number of Centres 24

7.3 Number of Participants 24

7.4 Trial objectives 25

7.5 Trial Outcome Measures 25

8 Selection and withdrawal of participants 25

8.1 Inclusion Criteria 25

8.2 Exclusion Criteria 26

8.3 Treatment Assignment and Randomisation Number 27

8.4 Method of Blinding 27

8.5 Participant withdrawal criteria 27

8.6 Early treatment termination28

9 Trial Treatments 28

9.1 Dosage schedules 29

9.2 Presentation of the drug 32

9.3 Known drug reactions & interaction with other therapies 33

9.4 Dosage modifications 33

9.5 Drug storage and supply 34

9.6 Concomitant therapy 35

10 Procedures and assessments 35

10.1 Screening evaluation 35

10.2 Baseline assessments (Week 0) [2 weeks +10 days] 37

10.3 Trial assessments 38

10.4 End of Trial Participation 42

10.5 Schedule of Events 42

10.6 Trial restrictions 45

11 Assessment of Safety 45

11.1 Definitions 45

11.2 Evaluation of adverse events 47

11.3 Reporting serious adverse events 47

11.4 Pregnancy Reporting 48

12 Toxicity – Emergency Procedures 48

13 Evaluation of Results (Definitions and response/evaluation of outcome 48

measures) 48

13.1 Response criteria 48

14 Storage and Analysis of Samples 49

15 Statistics 49

15.1 Statistical methods 49

15.2 Health Economics 52

15.3 Interim analyses 52

15.4 Number of participants to be enrolled 52

15.5 Criteria for the premature termination of the trial 52

15.6 Procedure to account for missing or spurious data 53

15.7 Definition of the end of the trial 53

16 Data handling and record keeping 53

16.1 CRF 53

16.2 Source Data 53

16.3 Endoscopic Video and MRE Images 54

16.4 Data Protection & Patient Confidentiality 54

17 Data Monitoring Committee/Trial Steering Committee 54

18 Ethical & Regulatory considerations 54

18.1 Consent 54

18.2 Ethical committee review 55

18.3 Regulatory Compliance 55

18.4 Protocol Amendments 55

18.5 Peer Review 55

18.6 Declaration of Helsinki and Good Clinical Practice 55

19 Sponsorship, Financial and Insurance 55

20 Monitoring, Audit & Inspection 56

21 Protocol Compliance and Breaches of GCP 56

22 Publications policy 56

23 References 57

24 Appendices 59

24.1 Appendix 1 - Trial Management / Responsibilities 59

24.2 Appendix 2 – Authorisation of Participating Sites 59

24.3 Safety Reporting Flow Chart 62

2 Abbreviations

6-MP	6-Mercaptopurine
6-TGN	6-Thioguanine
AE	Adverse Event
Anti-TNFα	Anti-Tumor Necrosis Factor Alpha
APR	Annual Progress Report
ASR	Annual Safety Report
CA	Competent Authority
CD	Crohn's Disease
CRF	Case Report Form
CRP	C-Reactive protein
DMC	Data Monitoring Committee
DSUR	Development Safety Update Report
FBC	Full Blood Count
GCP	Good Clinical Practice
GP	General Practitioner
HBI	Harvey Bradshaw Index
IBDQ	Inflammatory Bowel Disease Questionnaire
ISF	Investigator Site File
kg	Kilogram
LFTs	Liver Function Tests
mg	Milligram
ml	Millilitre
MRE	Magnetic Resonance Enterography (or Enteroclysis)

NIMP	Non Investigational Medicinal Product
PPC	Prescription prepayment certificate
QoL	Quality of life
R&D	Research and Development
RA	Regulatory Agency
REC	Research Ethics Committee
SAE	Serious Adverse Event
SmPC	Summary of Product Characteristics
TB	Tuberculosis
TMG	Trial Management Group
TPMT	Thiopurine Methyltransferase
TSC	Trial Steering Committee
U&Es	Urea and Electrolytes
VZV	Varicella Zoster Virus

3 Trial Synopsis

Title of clinical trial
**PR**edicting **O**utcomes **F**or Crohn's d**I**sease using a mo**L**ecular biomark**E**r (PROFILE) trial.
**Sponsor name**
Cambridge University Hospitals NHS Foundation Trust and the University of Cambridge.
**Medical condition or disease under investigation**
Crohn's disease.
**Purpose of clinical trial**
To demonstrate that a whole blood prognostic biomarker can be used at diagnosis to facilitate the delivery of appropriately personalised therapy in Crohn's disease, and that this improves clinical outcomes.
**Primary objective**
To demonstrate that a whole blood prognostic biomarker can improve outcomes by facilitating the delivery of personalised therapy from diagnosis in Crohn's disease.
**Secondary objective (s)**
To demonstrate that a whole blood prognostic biomarker can improve quality of life and health resource allocation by enabling appropriately personalised therapy to be initiated at diagnosis in Crohn's disease.

Trial Design
A randomised, multi-centre, biomarker-stratified open-label trial in patients newly diagnosed with Crohn's disease.
**Trial Outcome Measures**<br><br>**Primary endpoint**<br><br>• Incidence of sustained surgery and steroid free remission from completion of a protocolised (maximum 8-week regimen) steroid induction treatment through to week 48.

Secondary endpoints (3 assessed in parallel)
1. Endoscopic remission at week 48.<br>2. Quality of life assessment averaged across weeks 16, 32, 48 (using disease specific IBD-Q score).<br>3.i Number of flares requiring treatment escalation by week 48 primary follow-up period<br>3.ii Cumulative steroid exposure by week 48 primary follow-up period (defined by number of courses of steroids for active Crohn's disease).<br>3.iii Number of hospital admissions and surgeries related to Crohn's disease by week 48 primary follow-up period.
**Sample Size**<br><br>400 enrolled and stratified using biomarker.

Summary of eligibility criteria

Inclusion Criteria:

- Crohn's disease diagnosed within 6 months* using standard endoscopic, histologic or radiological criteria**
- Clinical evidence of active Crohn's disease

(corresponding to an HBI $\geq$ 7)***

- Endoscopic evidence of at least moderately active Crohn's disease.****
- CRP $\geq$ upper limit of normal on local assay **OR** Calprotectin $\geq$ 200 µg/g.
- Immunomodulator and anti-TNFα naïve.
- Aged 16-80 years old.

* Patients with newly-diagnosed patchy colonic inflammation, initially diagnosed as indeterminate colitis, would meet inclusion criteria for the trial if felt to be consistent with Crohn's disease.

** Patients need to have discontinued systemic steroids for one week or more prior to screening assessments and still have ongoing, active disease.

*** Patients with a HBI score of $\geq$7 within 2 weeks of screening visit, would be eligible for inclusion.

**** Screening endoscopy results can be taken from the participants' index colonoscopy, capsule endoscopy or flexible sigmoidoscopy performed as part of their standard care within 6 months of the screening visit

Exclusion Criteria:

- Patients with ulcerative colitis.
- Patients with active perianal sepsis or fistulating peri-anal Crohn's disease sufficient to mandate anti-TNF therapy.
- Patients with obstructive symptoms AND evidence of a fixed stricture on radiology or colonoscopy, which suggest that the patient is at high risk of requiring surgery over the following year. N.B. patients with modest degrees of stricturing on imaging but no obstructive symptoms may be included according to clinician judgement.
- Patients with contra-indications to trial medications including a history of

hepatitis B or C, tuberculosis.

- Patients with blood results that contra-indicate the medications used in the trial.
- Patients with active malignancy or recent malignancy with clinically estimated high risk of recurrence.
- Patients who are pregnant or breastfeeding at screening.
- Other serious medical or psychiatric illness currently on going, or experienced in the last 3 months, that could compromise the trial.
- Patients unable to comply with protocol requirements (for reasons including alcohol and/or recreational drug abuse).

Treatment arms

"Top-Down" therapy

- 8 week course of steroids started at screening. The rate of weaning should be accelerated once Infliximab is commenced, see section 9.1.1.

- Anti-TNFα Infliximab started 2 weeks after randomisation (±7 days) and any one of the following immunomodulatory medications (as tolerated) to be commenced within 6 weeks of starting infliximab as per local practice; Azathioprine OR 6-Mercaptopurine and Allopurinol OR Methotrexate, see section 9.1.1. Immunomodulatory medications will continue until end of trial. Combination therapy should be used unless otherwise clinically indicated. In such instances, monotherapy with anti-TNF agent can be given following approval from the Chief Investigator. If initial disease flare has not adequately responded by third dose of Infliximab (HBI $\geq$ 7) then an additional one-off dose of Infliximab should be given 4 weeks after their 3rd dose if pre-dose Infliximab serum levels from 3rd dose are below 20µg/ml (or upper limit of local assay where upper limit is less than 20µg/ml).

- Disease flares: 8 week course of steroids, see section 9.1.1.

"Accelerated Step-Up" therapy

- 8 week course of steroids started at screening, see section 9.1.1. If at baseline visit, patient remains significantly symptomatic from active Crohn's disease (fall of HBI <3 AND HBI $\geq$ 7) then an *ad hoc* visit should be arranged for week 2, with a view to moving onto Flare 1 step, as described below.

- Flare 1: 12 week course of steroids, see section 9.1.1., and one of the following medication options; Azathioprine OR 6-Mercaptopurine and Allopurinol OR Methotrexate (as tolerated), see section 9.1.1. If after approximately 2 weeks, symptoms remain refractory to the 12 week course of steroids and immunomodulator (fall of HBI <3 AND/OR HBI $\geq$ 7) or if they subsequently meet the protocol definition of a flare, the participant should be escalated as per Flare 2 step.

- Flare 2: Add in Infliximab within 5 weeks of confirming flare, see section

9.1.1. Combination therapy should be used unless otherwise clinically

indicated. In such instances, monotherapy with anti-TNF agent can be given following approval from the Chief Investigator. If the disease flare has not adequately responded by third dose of Infliximab (HBI $\geq$ 7) then an additional one-off dose of Infliximab should be scheduled to take place four weeks after this third dose. This additional visit should then take place if pre-dose Infliximab serum levels from third infusion visit are confirmed to be below 20µg/ml.<br><br>• Flare 3+ (i.e. disease flare after Infliximab dose optimisation as above):<br><br>8 week course of steroids, see section 9.1.1.
**Routes of administration**<br><br>Infliximab - Intravenous infusion.<br><br>Azathioprine – Oral tablet.<br><br>6-Mercaptopurine – Oral tablet.<br><br>Allopurinol – Oral tablet.<br><br>Methotrexate – Subcutaneous injection or oral tablet.<br><br>Steroids – Oral tablet
**Maximum duration of treatment of a participant**<br><br>Participants will follow the treatment regimens for the duration of the trial (approximately 50 weeks).

Procedures

<u>Screening Visit</u> (Week -2)

Consent, physical examination, HBI, pregnancy test (for women of childbearing age), blood sample for PAXgene tube x2 (biomarker assessment & research sample), serum tube, EDTA tube, blood samples (FBC, CRP, VZV, U&E, Creatinine, LFT, TPMT, Hepatitis B, Hepatitis C), assessment of latent and active TB as per local preference, sample pot for faecal Calprotectin provided, buffered stool sample, and start course of steroids (8 week course). Endoscopy result. MRE should be arranged if not already performed.

Results for VZV, TPMT, Hepatitis B, Hepatitis C within 1 year of the baseline assessment are acceptable. If any testing for TB subsequently returns as positive, then the participant should be withdrawn from the trial.

Baseline (Week 0) - i.e. 2 weeks after Screening visit (+10 days)

Eligibility confirmation, blood sample for PAXgene tube, medication review, physical examination, medical history and adverse event review, HBI, weight, patient rated and resource usage questionnaires. Blood tests. Randomisation of treatment allocation to either “Accelerated Step-Up” or “Top-Down” group.[†]

Week 4 (-10 days / +4 weeks)

Physical examination, HBI, weight, adverse event, medication review and blood tests.

**Weeks 16 & 32 (±4 weeks)**<br><br>As per week 4 visit, plus blood sample for PAXgene tube, serum tube, sample pot for faecal Calprotectin, and patient rated and resource usage questionnaires.<br><br>**Weeks 48 (±4 weeks)**<br><br>As per week 16 and 32 visit, plus buffered stool sample. End of trial procedures to be arranged (colonoscopy AND MRE).<br><br>**Ad hoc visits**<br><br>Ad hoc visits for either disease flares (HBI ≥ 7 AND [CRP ≥ upper limit of normal on local assay OR Calprotectin ≥ 200 µg/g] AND [Clinical opinion that symptoms are attributable to a flare of Crohn's disease]) [symptoms lasting for approximately 7 days*]; or issues with trial treatments in between scheduled trial visits.<br><br>*persisting symptoms present for (typically) 7 days or more (recognising that a true flare may be shorter in duration in some instances).<br><br>Similar to week 4 visit, plus stool sample for microscopy, culture and sensitivity, stool for faecal calprotectin and PAXgene tube (if treatment escalation for active disease, otherwise no PAXgene tube or faecal calprotectin samples required).<br><br>† Participants in the "Top-Down" group should be started on Infliximab within 2 weeks (± 7 days) after treatment allocation. This will align trial visits and dates of Infliximab infusion, to minimise extra visits.
**Treatment period**<br><br>The treatment period will be approximately 50 weeks. Following the trial treatment period, patients will resume standard care and be managed by their local clinicians.
**End of Trial**<br><br>Participants will complete their involvement in the trial approximately 50 weeks after the baseline visit.
**Procedures for safety monitoring during trial**<br><br>Monitoring for adverse events at each scheduled trial visit.
**Criteria for withdrawal of participants**<br><br>Each individual has the right to discontinue their participation in the trial at any time. If a participant wishes to discontinue, anonymised data collected up until that point will be included in the analysis.<br><br>For participants with non-response to Infliximab, please see section 9.4.

4 Trial Flow Chart

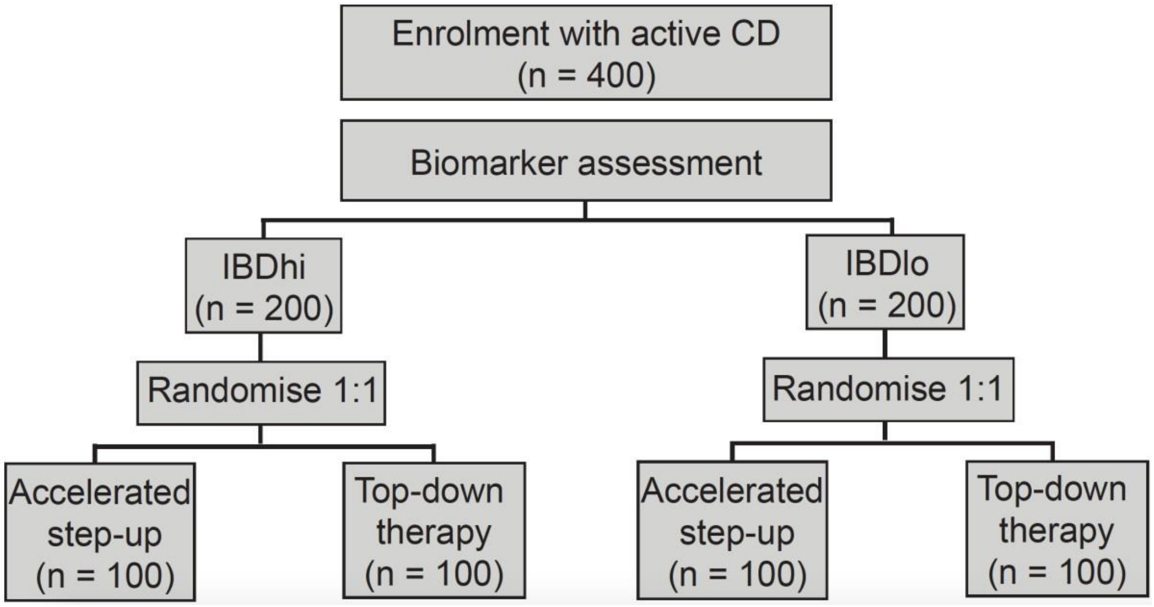

5 Introduction

5.1 Background

Crohn’s disease (CD) is a relapsing-remitting form of inflammatory bowel disease (IBD) that can affect any part of the intestinal tract, most commonly the ileum and the colon. It is a common condition, affecting at least 115,000 people in the United Kingdom, and typically presents in early adulthood. Crohn’s disease is a lifelong disease and is associated with symptoms including abdominal pain, diarrhoea, fatigue and weight loss. Up to 50% of patients may require surgery in the first 5-10 years after diagnosis.

Like many other immune-mediated diseases, the course of Crohn’s disease varies substantially between affected individuals, but no reliable prognostic markers exist. The most common treatment strategy in Crohn’s disease is based on a stepwise escalation in therapy, but only in response to recurrent flares or persistently active disease. This strategy (termed “Step-Up”) should not over-treat patients but could expose some to avoidable complications of persistently active disease (while weaker and potentially ineffective therapies are trialled). For this reason, other treatment strategies have been proposed.

In 2008, it was shown that early use of anti-TNFα therapy ("Top-Down") was superior to conventional "Step-Up" management (D'Haens et al. Lancet 2008). Further support came from the ‘study of biologic and immunomodulator naive patients in Crohn’s disease’ (SONIC) trial, which demonstrated that combining anti-TNFα (Infliximab) with Azathioprine achieved results superior to either alone (Colombel et al. NEJM 2010). However, it was widely recognised that the indiscriminate use of combination therapy in all patients would be (1) unaffordable and (2) risky – exposing patients with mild disease to the side effects of drugs that their disease did not require.

Since 2010, investigators have focused on whether accelerating more quickly up the treatment ladder ("Accelerated Step-Up"; REACT trial, Khanna et al. Lancet 2015) or using more affordable, but less potent, drugs (e.g. Azathioprine) at diagnosis in all patients would lead to better outcomes (AZTEC trial, Panes et al. Gastroenterology 2013; RAPID trial, Cosnes et al. Gastroenterology 2013). However, none of these trials demonstrated improved efficacy over standard care, leading many to conclude that a "precision" (or "personalised") approach is required. This conclusion stems from the fact that the course of Crohn’s disease varies substantially between patients, and thus is a major confounder in trials of treatment strategies. For all the work in recent years investigating clinical, genetic and serological markers, there are currently no prognostic tools for Crohn’s disease in clinical practice that can reliably predict the severity of the disease course from diagnosis.

To understand the importance of these issues to practicing gastroenterologists, a survey was conducted in the UK (n=50) and US (n=52), which showed that the majority of US and UK gastroenterologists recognised an unmet need for an assay that could predict the clinical outcome and probability of relapse in Crohn’s disease (UK 98%, US 94%). 54% of UK and 58% of US gastroenterologists would use such a test in their patients even if they could not change treatment in response to the results. If the results of the prognostic biomarker did enable gastroenterologists to tailor their treatment approach, 100% of the UK and US gastroenterologists sampled reported they would use the test.

5.2 Biomarker - Preliminary work

Previously, a transcriptional biomarker has been identified that is detectable within peripheral blood CD8 T cells from patients with active, untreated inflammatory bowel disease, as well as in other autoimmune diseases (Lee et al. JCI 2011, McKinney et al. Nature Med 2010). This gene expression signature was detectable at diagnosis and stratified all IBD patients into 2 clinically indistinguishable subgroups (termed "IBD1" and "IBD2"). Detailed, prospective follow-up demonstrated that patients within these subgroups experienced significantly different disease courses (Figure A). Those in the IBD1 subgroup had a high incidence of treatment-refractory, relapsing disease, while those in the IBD2 subgroup typically achieved stable remission on minimal immunosuppression. The prognostic stratification that was achieved using this biomarker was superior to that observed using previously described methods, including clinical parameters and ASCA serology.

To validate this observation, a further 51 patients were recruited with newly diagnosed IBD. Analysis of their CD8 T cell transcriptomes replicated the prognostic stratification observed in the initial dataset, providing independent validation of the signature and its ability to predict disease outcome (data on file). Overall performance: Hazard ratio 3.53 (95% confidence intervals: 2.09 - 5.93), P < 0.0001 (escalation-free survival); mean number of escalations 1.41 (IBD1), 0.69 (IBD2), P = 0.0001, Relative Risk = 2.03 (total number of escalations during follow up - median 3.5 years in both subgroups), n = 118.

5.3 Biomarker – Data development of whole blood classifier

A whole blood qPCR assay has now been developed and validated that re-capitulates the IBD1/IBD2 subgroups, which were originally termed PAX1 and PAX2 respectively (Figures A and B), and have now been re-named IBD^{hi} and IBD^{lo}. This classifier demonstrates an equivalent association with disease course, both in terms of escalation-free survival (P = 0.0074) and number of escalations over time (P = 0.0003, mean number of treatment escalations: 1.62 (IBD^{hi}), 0.70 (IBD^{lo})). The genes in this classifier were taken forward to real time PCR assay development, where the final content of the whole blood classifier (16 informative and 2 reference genes) was optimised and finalised.

Independent validation of the prognostic performance of this classifier was then sought using a second, independent cohort of 85 newly diagnosed IBD patients. Analysis of whole blood gene expression using the qPCR-based test replicated the prognostic stratification seen in the discovery cohort with a IBD^{hi}/IBD^{lo}. Hazard ratio of 3.52 (95 percent CI: 1.84 6.76, P = 0.0002, Figure C). In the training cohort the ratio of IBD^{hi}:IBD^{lo} was 35:34. In the validation cohort the ratio of IBD^{hi}:IBD^{lo} was 42:43.

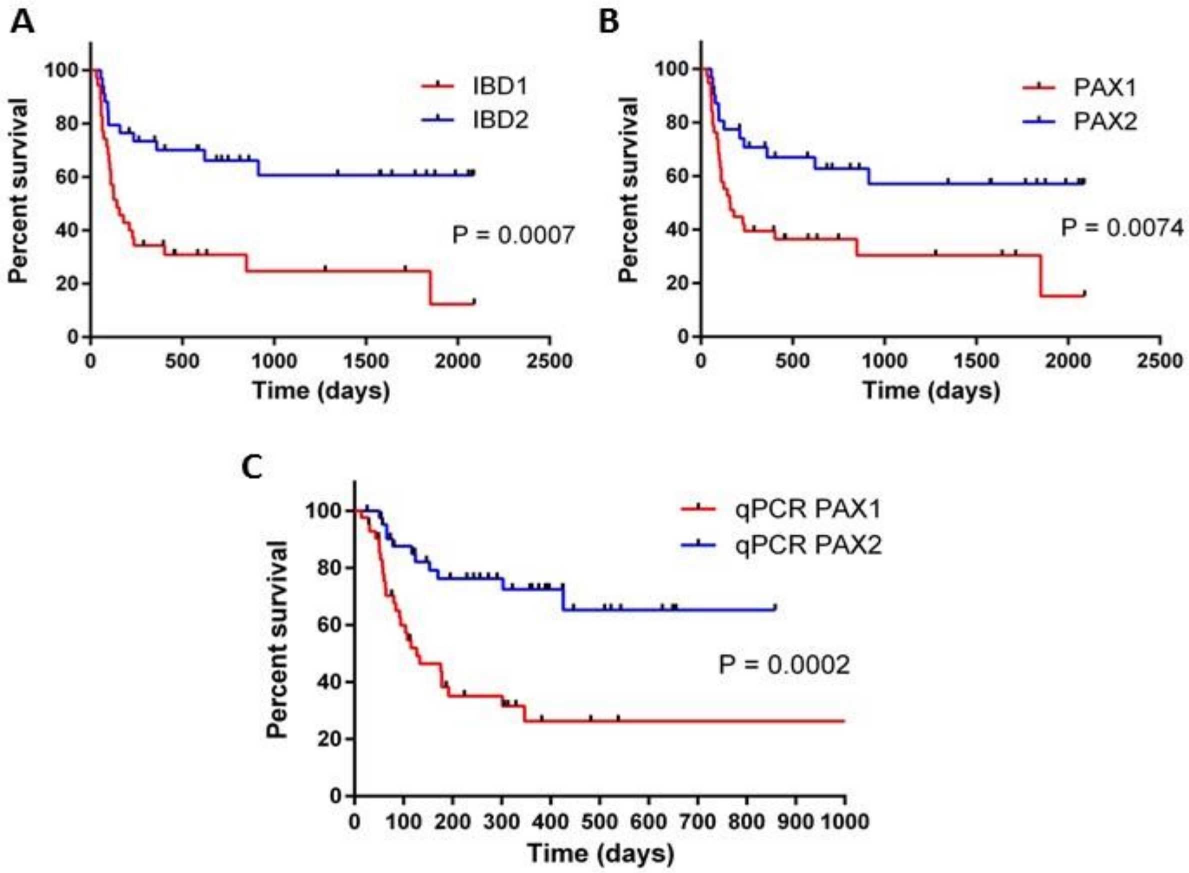

Hazard ratio = 3.52 (95% CI 1.84-6.76)

Development and validation of whole blood qPCR classifier

(**A and B**) Classifier development: Kaplan-Meier survival curves demonstrating the proportion of IBD patients in the training phase of classifier development (n = 69) who did not require a treatment escalation (immunomodulator or surgery) during 4 years' prospective follow up, as stratified by CD8 T cell IBD1/2 subgroup (A) and whole blood qPCR assay (B). (**C**) Kaplan-Meier curve demonstrating the performance of the qPCR classifier in an independent cohort of 85 newly diagnosed IBD patients recruited from 4 UK centres.

6 Rationale for Trial

The hypothesis is that biomarker-driven stratification will facilitate personalised therapy in Crohn's disease, and will improve clinical care. It will do this through identification of a group of patients destined to develop a more severe, relapsing course and who will benefit from "Top-Down" therapy while protecting those patients destined to experience indolent disease from the risks and side-effects of unnecessary immunosuppression.

7 Trial Design

7.1 Statement of design

This is a randomised, multi-centre, biomarker-stratified, open-label trial in patients newly diagnosed with Crohn's disease (enrolled within 6 months of diagnosis).

This is an established trial design for the validation of predictive biomarkers, (Friedlin et al. J Natl Cancer Inst 2010) which has been used extensively in oncology settings (Potti et al. NEJM 2006). Participants will be randomised to either "Top-Down" or "Accelerated Step-Up" therapy.

7.2 Number of Centres

The trial will be conducted in approximately 50 centres in the United Kingdom.

7.3 Number of Participants

400 participants will be randomised in this trial.

7.3.1 Participants Trial duration

Participants will be followed-up for approximately 50 weeks from the baseline visit. There will be a total of 6 mandatory trial visits, during which data will be collected for participants in both treatment arms.

In addition to planned trial visits, participants within both arms of the trial may be seen *ad hoc* as required to manage disease flares or issues relating to trial treatments.

Trial visits will take place at the same time points for both treatment arms of the trial. These have been timed to coincide with Infliximab infusion visits where possible (for those who are randomised to "Top-Down").

For participants randomised to "Accelerated Step-Up" treatment, if disease progression requires treatment escalation to Infliximab, this will be undertaken at standard intervals i.e. weeks 0, 2, 6 and 8 weekly thereafter.

Future data collection will also take place following completion of the randomised treatment to assess disease burden and the longer term impact of "Top-Down" vs. "Accelerated Step-Up" treatment approaches on subsequent disease course. This will be based on questionnaire data and clinical assessment.

7.4 Trial objectives

7.4.1 Primary objective

 To demonstrate that a whole blood prognostic biomarker can improve clinical outcomes by facilitating the delivery of personalised therapy from diagnosis in Crohn's disease.

7.4.2 Secondary objective

 To demonstrate that a whole blood prognostic biomarker can improve quality of life and health resource allocation by enabling appropriately personalised therapy to be initiated at diagnosis in Crohn's disease.

7.5 Trial Outcome Measures

7.5.1 Primary outcome measure

Incidence of sustained surgery and steroid free remission from completion of a protocolised (maximum 8 week regimen) steroid induction treatment through to week 48*.

*Remission at each visit is a composite of two conditions

- HBI score <=4
- Absence of objective evidence of inflammation: both CRP<=ULN and calprotectin<200 μg/g. If both values are missing then the condition is deemed missing. If just one value is missing then it is assumed to be below the threshold.

If either or both conditions hold then the participant is in remission at the visit. Requirement for a course of systemic steroids for active Crohn's disease would result in failure to meet primary outcome measure.

7.5.2 Secondary outcome measure

1. Endoscopic remission at week 48.
2. Quality of life assessment averaged across weeks 16, 32, 48 (using disease specific IBD-Q score).

3.i Number of flares requiring treatment escalation by week 48 primary follow-up period.

3.ii Cumulative steroid exposure by week 48 primary follow-up period (defined by number of courses of steroids for active Crohn's disease).

3.iii Number of hospital admissions and surgeries related to Crohn's disease by week 48 primary follow-up period.

8 Selection and withdrawal of participants

8.1 Inclusion Criteria

Patients to be included in the trial must meet the following criteria:

- Crohn’s disease diagnosed within 6 months* using standard endoscopic, histologic or radiological criteria**
- Clinical evidence of active Crohn’s disease (corresponding to an HBI ≥ 7)***
- Endoscopic evidence of at least moderately active Crohn’s disease****
- CRP ≥ upper limit of normal on local assay **OR** Calprotectin ≥ 200 µg/g*****
- Immunomodulator and anti-TNFα naïve
- Aged 16-80 years old

* Patients with newly-diagnosed patchy colonic inflammation, initially diagnosed as indeterminate colitis, would meet inclusion criteria for the trial if felt to be consistent with Crohn’s disease.

** Patients need to have discontinued systemic steroids for one week or more prior to screening assessments and still have ongoing, active disease.

*** Patients with an HBI score of ≥ 7 within 2 weeks of screening visit would be eligible for inclusion.

**** Grading of severity will be based on clinical impression of endoscopist or clinical team managing Crohn's disease and as a guide would correspond to an approximate SES-CD of 4 or more for ileal-only disease and score of approximately 6 or more for ileocolonic or colonic disease distributions.

Endoscopic results used for confirming inclusion can be from the participants index colonoscopy performed as part of their standard care. Ideally however, a video of the index colonoscopy should be recorded wherever possible. If this is not possible, photos of the colonoscopy or colonoscopy report can be used to confirm inclusion. Results from capsule endoscopies and flexible sigmoidoscopy are also permitted to confirm inclusion.

***** Results of blood tests and faecal calprotectin performed as part of standard care can be used for eligibility provided performed up to 3 months prior to the baseline visit – however a repeat stool sample should still be sent for central processing (as central laboratory results will be used in subsequent analyses). Sample pot for faecal calprotectin will be provided at screening visit and patients advised to provide sample promptly so that central calprotectin result is available at the baseline visit.

8.2 Exclusion Criteria

The presence of any of the following will preclude patient inclusion:

- Patients with ulcerative colitis.
- Patients with active perianal sepsis or fistulating peri-anal Crohn’s disease sufficient to mandate anti-TNF therapy.

- Patients with obstructive symptoms AND evidence of a fixed stricture on radiology or colonoscopy, which suggest that the patient is at high risk of requiring surgery over the following year. N.B. patients with modest degrees of stricturing on imaging but no obstructive symptoms may be included according to clinician judgement.
- Patients with contra-indications to trial medications.
- Patients with blood results that contra-indicate the medications used in the trial including a history of hepatitis B or C, tuberculosis.
- Patients with active malignancy or recent malignancy with clinically estimated high risk of recurrence.
- Patients who are pregnant or breastfeeding at baseline.
- Other serious medical or psychiatric illness currently on going, or experienced in the last 3 months, that could compromise the trial.
- Patients unable to comply with protocol requirements (for reasons including alcohol and/or recreational drug abuse).

Patients who still have a TPMT result awaited when they attend their baseline visit can still be randomised if otherwise eligible. The TPMT result will need to be confirmed before the patient can start on a thiopurine. For participants who are found to be TPMT null, then the immunomodulator of choice would be Methotrexate (as described in 9.1.1).

If any outstanding testing for TB subsequently returns as positive, then the participant should be withdrawn from the trial.

8.3 Treatment Assignment and Randomisation Number

Following biomarker assessment, participants in each biomarker subgroup will be randomly assigned (1:1) to either "Top-Down" or "Accelerated Step-Up" therapy, using a computer-generated algorithm (Sealed Envelope). This will occur 14 days after screening (plus 10 days).

Stratified block randomisation will be used, stratifying on biomarker subgroup, (IBD^{hi}/IBD^{lo}), mucosal inflammation (mild / moderate / severe) and disease location (colon-only/other) with a randomly generated block size.

8.4 Method of Blinding

All treatments will be open label. Clinicians and participants will, however, be blinded to biomarker subgroup allocation.

8.5 Participant withdrawal criteria

Each patient has the right to discontinue their participation in the trial at any time. If a patient asks to be withdrawn a partial withdrawal may be offered.

Partial withdrawals are allowed, e.g. patient no longer wishes to complete questionnaires but is happy to continue otherwise.

All withdrawals and partial withdrawals will be documented. If a patient wishes to discontinue, anonymised data collected up until that point will be included in the analysis.

8.6 Early treatment termination

All participants are expected to remain in the trial with medical management of their Crohn's disease as protocolised, until the final scheduled visit at week 48.

Participants will be reviewed at each visit and early treatment termination will be at the discretion of the participant and local treating team, if felt that early treatment termination is in the participant's best interests.

If early treatment termination occurs, this should be documented as a partial withdrawal and all remaining follow up visits should be completed as normal.

9 Trial Treatments

For the purposes of this trial, all the drugs to be used are classed as NonInvestigational Medicinal Products (NIMPs):

Steroids

- Prednisolone
- Budesonide

Infliximab

Adalimumab

Azathioprine

6-Mercaptopurine

Methotrexate

Allopurinol

Folic acid

Chlorphenamine

9.1 Dosage schedules

"Top-down" therapy

- 8 week course of steroids started at screening, see section 9.1.1. The rate of weaning should be accelerated once Infliximab is commenced.
- Infliximab started within 2 weeks after randomisation (± 7 days) **plus** any one of the following immunomodulatory medications (as tolerated) to be commenced within 6 weeks of starting infliximab as per local practice; Azathioprine OR 6-Mercaptopurine and Allopurinol OR Methotrexate and Folic acid, see section 9.1.1. Immunomodulatory medications will continue until end of trial. Combination therapy should be used unless otherwise clinically indicated. In such instances, monotherapy with anti-TNF agent can be given following approval from the Chief Investigator.
- If participants are either severely or persistently intolerant of Infliximab or develop anti-infliximab antibodies, they may be switched onto Adalimumab (see section 9.1.1) and should continue or adjust immunomodulatory at clinician's discretion.
- Disease flares: Add in 8 week course of steroids, see section 9.1.1. For clarity, non-response to Infliximab after the 3 induction doses should not be classed as a disease flare and does not mandate a course of steroids at this stage, but rather the management plan as described below.

* For participants in the "Top-Down" group with non-response to Infliximab (HBI $\geq$ 7 at the 3rd dose of Infliximab), a blood sample for Infliximab trough drug level should be taken and an additional dose of Infliximab will be provisionally scheduled for 4 weeks (±10 days) after their 3rd dose, see section 9.4.

This additional Infliximab infusion should only be given if pre-dose Infliximab levels, taken at the 3rd infliximab infusion date, are confirmed to be less than 20µg/ml (or upper limit of local assay where upper limit is less than 20µg/ml) and the disease remains active at 3 weeks after their 3rd dose (checked via telephone consultation). Participants with persistent non-response after their 4th dose should have early treatment termination; with end of trial procedures performed and revert back to standard care with their local clinical team (see section 8.6).

The need for a course of steroid treatment for active Crohn's disease, once the patient has completed Infliximab induction therapy and is on the maintenance regimen, means that they have failed to meet the primary outcome measure.

“Accelerated Step-Up” therapy

- **8 week course** of steroids started at screening, see section 9.1.1. If at baseline visit, patient remains significantly symptomatic from active Crohn’s disease (fall of HBI <3 AND HBI $\geq$ 7) then an *ad hoc* visit should be arranged for week 2, with a view to moving onto Flare 1 step, as described below.

- Flare 1: **12 week course** of steroids, see section 9.1.1., and one of the following medication options; Azathioprine OR 6-Mercaptopurine and Allopurinol OR Methotrexate (as tolerated), see section 9.1.1. If after approximately 2 weeks symptoms remain refractory to the 12 week course of steroids, and immunomodulator (fall of HBI <3 AND/OR HBI $\geq$ 7) or if they subsequently meet the protocol definition of a flare , the participant should be escalated as per Flare 2 step.

- Flare 2: Add in Infliximab within 5 weeks of confirming a flare, see section 9.1.1. Combination therapy should be used unless otherwise clinically indicated. In such instances, monotherapy with anti-TNF agent can be given following approval from the Chief Investigator. If the disease flare has not adequately responded by third dose of Infliximab (HBI $\geq$ 7) then an additional one-off dose of Infliximab should be scheduled to take place four weeks (±10 days) after this third dose. This additional visit should then take place if pre-dose Infliximab serum levels from third infusion visit are confirmed to be below 20µg/ml (or upper limit of local assay where upper limit is less than 20µg/ml).

- Flare 3+: 8 week course of steroids for disease flare after Infliximab induction doses as detailed for flares in Top Down (see previous page and section 9.1.1.

9.1.1 Route of Administration and Maximum dosage allowed

Steroids

All participants commence an 8-week course of steroids at screening

Dose modification and route of administration of steroids is permitted at clinical discretion. For participants in the ‘Top Down’ arm, this 8 week course can be shortened once they have received anti-TNF as per Section 9.4.

Oral vitamin D and calcium replacement should be prescribed as per local hospital guidelines, whilst on all regimen of steroids.

8 week course started at screening:

Examples of a typical 8 week regimen include either 40mg Prednisolone dose to be taken daily, reducing by 5mg every week to 0mg, or, 9mg Budesonide dose to be taken daily.

12 week course started at Flare 1 in Step-Up arm

If participants meet the protocol definition of a flare (HBI ≥ 7 AND [CRP ≥ upper limit of normal on local assay OR Calprotectin ≥ 200 µg/g] AND [Clinical opinion that symptoms are attributable to a flare of Crohn’s disease]) the participant will move to a 12 week course of steroids plus immunomodulator. This course can be shortened once they have received anti-TNF.

Examples of a typical 12 week regimen include either 40mg Prednisolone dose to be taken daily, reducing by 5mg every week to 20mg/day then 5mg reduction every 2 weeks to 0 plus starting an immunomodulator, or 9mg Budesonide dose to be taken daily for 8 weeks then reducing by 3mg every 2 weeks to 0 **plus** starting an immunomodulator.

<u>Azathioprine</u>

For participants with normal TPMT activity who are aged less than 65 years old, Azathioprine 2.5mg/kg oral tablet(s) to be taken daily. It is recommended for the PROFILE trial, that participants start on a 2.5mg/kg dose. However, if dose escalation is to be performed then participants can be escalated at clinician's discretion e.g. started on 50mg per day for 2 weeks to ensure tolerance, before directly escalating to this 2.5mg/kg dose.

For participants with intermediate TPMT activity this dose should be halved. Participants with complete TPMT deficiency or aged >65 years old, should not receive Azathioprine, and should ideally receive Methotrexate instead. Specific dosing may vary from the exact calculated dosing due to tablet configurations (dose rounding/tablet splitting/alternate day dosing to be guided as per local practice). Dosing may be adjusted based on symptoms, white blood count, liver enzymes and metabolite levels, as per clinician judgement see section 9.4.

<u>Azathioprine intolerance</u>

Participants who are intolerant of Azathioprine should be started on either Methotrexate or low dose 6-Mercaptopurine with Allopurinol (details below) (if tolerated) as per the preference of the local Investigator, unless Azathioprine-induced pancreatitis occurred in which case Methotrexate must be used.

<u>Low Dose 6-Mercaptopurine (6-MP) & Allopurinol combination</u>

For participants who are intolerant of Azathioprine (for reasons other than pancreatitis) or who preferentially metabolise Azathioprine to 6-MMP: start Allopurinol 100mg oral tablet(s) and 6-Mercaptopurine 0.3-0.4mg/kg oral tablet(s) to be taken daily (dose rounding/tablet splitting/alternate day dosing to be guided as per local practice). Specific dosing may vary from the exact calculated dosing due to tablet configurations. The combination of Allopurinol and low dose Mercaptopurine is safe provided appropriate monitoring, including therapeutic drug monitoring (Smith et al. J Crohns Colitis 2012). Dosing may be adjusted, please see section 9.4. Women of child-bearing age or men who are sexually active receiving Allopurinol must use contraception.

<u>Methotrexate & Folic acid combination</u>

For participants who are TPMT null, or older than 65 years, or who develop Azathioprine-induced pancreatitis, Methotrexate 15-25mg is to be taken weekly (no titration), either as oral tablet(s) or subcutaneous injection. Lower doses may be required if any evidence of impaired renal or liver function. Women of child-bearing age or men who are sexually active receiving Methotrexate must use contraception. Folic acid should to be taken as an oral tablet for participants on Methotrexate, the dosing of Folic acid should be as per standard practice at local hospital.

Anti-TNFα Infliximab

For participants in the "Top-Down" group or Step-Up Flare 2, Infliximab 5mg/kg to be infused intravenously, over a period of up to 2 hours. Following first infusion, subsequent infusions will be at 2 weeks after (± 7 days), then 6 weeks after the first infusion (± 7 days), and then infused 8 weekly thereafter (± 7 days), i.e. standard induction and treatment regimen for Infliximab. Exact dosing may vary slightly from the calculated dose e.g. dose may be rounded up or down in line with local practice. Women of child-bearing potential should consider the use of adequate contraception to prevent pregnancy and continue its use for at least 6 months after the last infliximab treatment.

Chlorphenamine and Hydrocortisone

Follow local standard practice for the use of pre-infusion treatment given as per local guidelines.

Anti-TNFα Adalimumab

If participants are either severely intolerant of Infliximab or experience persistent intolerance despite a slower infusion rate and pre-treatment as per local guidelines, or if the participant has developed anti-infliximab antibodies, then they can be switched onto Adalimumab only after discussion with the Chief Investigator. This is delivered subcutaneously every two weeks. First dose is 160mg, second dose is 80mg and all further doses are 40mg. Participants should continue or adjust immunomodulator at clinician's discretion. Women of child-bearing potential should consider the use of adequate contraception to prevent pregnancy and continue its use for at least 5 months after the last adalimumab treatment.

9.1.2 Maximum duration of treatment of a participant

Participants will follow the medication regimen they are randomised to for the duration of the trial (approximately 50 weeks from baseline). At the end of the trial, treatment for participants will be at the discretion of their treating clinician and funded locally, as per usual clinical practice.

9.1.3 Procedures for monitoring participant compliance

For all medications except Infliximab, participants would take home their treatments and self-administer at appropriate time intervals. Significant non-adherence will be noted in patient notes.

9.2 Presentation of the drug

UK and EU-licensed standard commercial stock will be used for all NIMPs.

9.3 Known drug reactions & interaction with other therapies

Please refer to the current SmPC for information about drug reactions and interactions for the following medications:

Infliximab (Remsima 100mg powder)

Adalimumab (Humira 40mg/0.4ml solution)

Azathioprine (50mg Tablets)

6-Mercaptopurine (50mg Tablets)

Methotrexate (Maxtrex Tablets 10 mg)

Allopurinol (100mg Tablets)

Folic acid (Folic Acid Tablets BP 5 mg)

Chlorphenamine (Boots 4mg Tablets)

9.4 Dosage modifications

Steroids For participants in the “Top-down” treatment arm, the rate of weaning of the 8 week course of steroids started at screening can be accelerated once the participant has started Infliximab in order to minimise the amount of time any “Top-Down” participant might be receiving triple immunosuppression. If using Prednisolone, recommended rate of reduction of 5mg/week to 10mg/week. Antibiotic prophylaxis whilst on triple immunosuppression can be used at clinician discretion, according to local practice.

Azathioprine and 6-Mercaptopurine

Dosing may be adjusted for weight, TPMT levels, white blood count, liver enzymes or following measurement of 6-TGN and 6-MMP levels with targets as per normal reference ranges of local laboratory (e.g. 6-TGN levels approximately 235-450pmol/8 x 10^8).

If participants taking Azathioprine develop intolerable side-effects, either Methotrexate or low dose 6-Mercaptopurine and Allopurinol should be prescribed instead (as tolerated), unless the side-effects specifically relate to thiopurines. If this occurs, Methotrexate should be prescribed instead (if no contra-indication). If intolerant of second or third-line immunomodulator (Methotrexate or low dose 6-Mercaptopurine and Allopurinol) then patient should escalate to Infliximab, stop immunomodulators and remain in the trial on infliximab as monotherapy. Any decision to move a participant onto infliximab monotherapy needs prior approval from the Chief Investigator.

Combination therapy should be used unless otherwise clinically indicated. In such instances, monotherapy with anti-TNF agent can be given following approval from the Chief Investigator.

Infliximab

Participants in the "Top-Down" group with sub-optimal response to Infliximab (HBI $\geq$ 7 at 3rd infliximab infusion date), should be provisionally booked for an additional Infliximab infusion (5mg/kg) to occur 4 weeks later (± 10 days). This infusion will be given if the trough Infliximab level, from date of 3rd Infliximab infusion, is less than 20µg/ml (or upper limit of local assay where upper limit is less than 20µg/ml) and the disease remains active at prior to this additional dose (confirmed via telephone consultation). If non-response to Infliximab persists after week 16 the participant should have early treatment termination (see section 8.6). Treatment costs and management decisions outside the trial protocols will revert back to the treating clinician and respective local funding.

If mild intolerance to Infliximab (including non-severe infusion reactions or other mild allergic symptoms such as flushing, rash, itch, urticaria, fatigue, and/or headaches) then local standard practice should be followed e.g. reduce infusion rate, use of chlorphenamine/hydrocortisone etc.

If symptoms of intolerance persist, or if the participant has developed anti-infliximab antibodies they, should be switched onto Adalimumab only after discussion with the Chief Investigator. For participants with a severe allergic reaction to Infliximab, such as bronchospasm, upper airway oedema, or hypotension, Infliximab should be stopped and patient should be considered to commence on Adalimumab, only after discussion with the Chief Investigator (see section 9.1.1).

Legal status of the drug

All medications included within the PROFILE trial are licensed medications and all are being widely used in the setting of Crohn's Disease within the UK and Europe.

9.5 Drug storage and supply

Infliximab and Adalimumab

Commercial supply of Infliximab will be available to order from the pharmaceutical company used by the trial team. This will be available free of charge to recruiting hospitals for participants taking part in the PROFILE trial. A trial order code will be supplied to pharmacies, which they will need to order against to receive the free rate.

Infliximab costs will only be covered for up to a maximum of 9 separate infusions per participant over the course of the trial. For the first order of Infliximab for the first participant recruited by each trial site, the trial co-ordinator will be required to approve the order. This stock will be stored in pharmacies as per the SmPC.

For participants who require Adalimumab due to Infliximab intolerance or develop antiinfliximab antibodies, this will be paid for by the lead site in Cambridge, over the course of the trial. Storage of this medication will be in line with the SmPC.

Azathioprine, 6-Mercaptopurine, Allopurinol, Methotrexate, Folic acid, steroids and Chlorphenamine

The following medications; Azathioprine, 6-Mercaptopurine, Allopurinol, Methotrexate, Folic acid, steroids and Chlorphenamine will prescribed as per standard practice from either local hospital stock or in the community. Local arrangements should be made to ensure the costs of these medications are NOT met by the trial participant. This can be either through issue of a prescription prepayment certificate (PPC) to participants at the randomisation visit or reimbursement of the cost of prescriptions, or any other suitable alternative.

These medications will be collected by participants, as per arrangements at their local hospital. They will be collected from a hospital pharmacy or community pharmacy, following prescription by the clinician managing their Crohn's Disease.

Storage of all medications will be in line with the SmPC.

9.6 Concomitant therapy

Apart from the trial treatments allocated at randomisation, all other aspects of patient management are at the discretion of the participants' clinicians.

10 Procedures and assessments

Assessments and data collection will be performed, according to the schedule of events, by appropriately trained and qualified research staff as delegated by the Principal Investigator. This could include members of the Clinical Research Network teams.

At each trial visit safety/AE/SAE review will take place and treatment compliance will be reviewed.

10.1 Screening evaluation

10.1.1 Identification of potential participants

Potential trial patients with newly diagnosed Crohn's Disease will be identified by a member of the clinical team. Potentially eligible participants will be given a Participant Information Sheet (PIS). Some potential patients may be due to have their endoscopic procedure to take place when seen by member of clinical team. Consent for videorecording of endoscopic procedure should be obtained as routine for any patient due to undergo an endoscopic procedure (for later evaluation as part of the trial).

For any hospitals that do not have facilities in place for video recording colonoscopy, the lead site in Cambridge will supply equipment if required to make this possible e.g. video capture USB sticks and external video capture devices.

10.1.2 Screening assessments

Trial specific assessments will only be conducted after patients have given written informed consent.

All participants will be screened for eligibility based on the inclusion/exclusion criteria outlined in Section 8.1 and 8.2. The screening assessments required are;

Patient data to collect

- Demographics
- HBI
- Perianal disease review
- Weight in kg
- Height in cm
- Physical examination
- Medical history (previous/current medications and smoking)
- Concomitant medications
- Blood Results Review
- Adverse events
- Assessment of latent and active TB infection
- Results of endoscopic procedure performed within 6 months of the screening assessment visit (including video-recording of procedure where possible).
- Results and images of MRE: either within 6 months prior to screening if performed as part of standard care OR if not already performed, then within 3 months of the screening visit.
- IBDQ & EQ-5D, patient rated quality of life measures

Samples to be collected and processed **locally**

- Results of hepatitis B & C and Varicella Zoster Virus (VZV) blood test, results within 1 year of the baseline assessment are acceptable.
- Results of TPMT testing, results within 1 year of the baseline assessment are acceptable.
- Pregnancy test for female participants (if applicable) and breast feeding status.
- Full blood count.
- Biochemical series (including urea, creatinine, electrolytes, liver function tests, CRP).

Samples to be collected and sent, to be processed **centrally**

- PAXgene RNA tube x2 (biomarker assessment & research sample), Serum tube, EDTA tube
- Stool sample for faecal Calprotectin.
- Buffered stool sample (advise patient to collect same day as screening visit and ideally before starting steroid treatment).

Participants will be registered on Sealed Envelope and a trial ID will be assigned. Patients must have active disease (HBI $\geq$ 7) and not be on any steroid or immunomodulator treatment at time of recruitment. Patients who have been diagnosed within the last 6 months and have already completed a regimen of steroids would be eligible for inclusion if their disease remains active. Patients need to have discontinued steroids for one week or more prior to screening assessments and still have ongoing, active disease.

An 8 week course of steroids will be started at screening. This is to ensure any potential participants are not left with active disease without any form of treatment prior to randomisation.

Participants should be routinely called by their local treating team approximately three working days after their screening visit to ensure the stool samples have been sent, in the provided delivery safeboxes.

10.1.3 Participant Randomisation

Clinical details will be documented on a CRF and baseline samples obtained.

After analysis of PAXgene biomarker assay sample, participants will be assigned to either IBD^{hi} or IBD^{lo} groups. Patients will then be randomised using a stratified block randomisation technique via a web based system (Sealed Envelope), to either "TopDown" or "Accelerated Step-Up" therapy and their trial site will be informed of treatment allocation.

If patients cannot be assigned an IBD^{hi} or IBD^{lo} subgroup, following analysis of their PAXgene sample, then they will be excluded from the trial. Trial sites will also be informed if patient has been excluded from the trial due to presence of exclusion criteria, highlighted in 9.2.

10.2 Baseline assessments (Week 0) [2 weeks +10 days]

Patient data to collect:

- HBI
- Perianal disease review
- Significant past medical history
- Confirmation of eligibility
- Concomitant medications
- Adverse events
- Blood results review
- Weight in kg
- Physical examination
- IBDQ & EQ-5D patient rated quality of life measures
- Resource usage patient questionnaire

Samples to be collected and processed **locally:**

- Full blood count

- Biochemical series (including urea, creatinine, electrolytes, liver function tests, and CRP)

Samples to be collected and sent, to be processed **centrally:**

- PAXgene tube

†Previous/current malignancy, previous/current tuberculosis, previous/current hepatitis B virus, previous/current hepatitis C virus, significant mental health history including alcohol or drug abuse, this list is not exhaustive.

*If any outstanding testing for TB subsequently returns as positive, then the participant should be withdrawn from the trial.

Definition of active disease at trial inclusion

HBI ≥ 7 **AND** [active Crohn's disease on endoscopy] **AND** [CRP ≥ upper limit of normal on local assay **OR** Calprotectin ≥ 200 µg/g].

10.3 Trial assessments

Colonoscopy

Video of withdrawal from ileum ideally to be recorded, for central reading. Severity should be objectively scored by an endoscopist using the Simple Endoscopic Score for Crohn's Disease (SES-CD), to allow comparison with central reading assessment and aid assessment for mucosal healing. Routine ileal and colonic biopsies should be taken as per local clinical practice.

Capsule endoscopy and flexible sigmoidoscopy images should ideally be captured. Severity should be assessed by an endoscopist.

MR enterography or enteroclysis

Entire procedure images will be used for central reading. This will be scored by central reading team; using the Magnetic Resonance Index of Activity (MaRIA) score.

10.3.1 Timing of assessments

Patients are to be seen in clinic at Screening, Baseline, Weeks 4, 16, 32 and 48. Baseline visit should be 2 weeks +10 days from the screening visit. Weeks 4 to 48 are calculated from the Baseline visit. Week 4 should be -10 days or + 4 weeks from calculated visit date. Weeks 16 to 48 should be ± 4 weeks of the calculated visit date.

Definition of disease flares for the duration of the trial

HBI ≥ 7 (symptoms for approximately 7 days*) **AND** [CRP ≥ upper limit of normal on local assay **OR** Calprotectin ≥ 200 µg/g] **AND** [Clinical opinion that symptoms are attributable to a flare of Crohn's disease].

*persisting symptoms present for (typically) 7 days or more (recognising that a true flare may be shorter in duration in some instances)

10.3.2 Assessments at specific time points:

Week 4 (-10 days / + 4 wks) Patient data to collect:

- HBI.
- Perianal disease review.
- Treatment compliance check.
- Concomitant medications.
- Adverse events.
- Weight in Kg.
- Physical examination.
- Blood results review.
- Primary outcomes review (steroids and surgery).

Samples to collect and to be processed **locally:**

- Full blood count.
- Biochemical series (including urea, creatinine, electrolytes, liver function tests, CRP), [thiopurine metabolites if taking Azathioprine or 6-Mercaptopurine].

Week 16 (±4wks)

Patient data to collect:

- HBI.
- Perianal disease review.
- Treatment compliance check.
- Concomitant medications.
- Adverse events.
- Weight in Kg.
- Physical examination.
- Blood results review.
- IBDQ & EQ-5D patient rated quality of life measures.
- Resource usage patient questionnaire.
- Index MRE data.
- Primary outcomes review (steroids and surgery).

Samples to be collected and processed **locally:**

- Full blood count.
- Biochemical series (including urea, creatinine, electrolytes, liver function tests, CRP), [thiopurine metabolites if taking Azathioprine or 6-Mercaptopurine].

Samples to be collected and sent, to be processed **centrally:**

- PAXgene tube.
- Serum tube.
- Stool sample for faecal Calprotectin.

<u>Week 32 (±4wks)</u>

Patient data to collect:

- HBI.
- Perianal disease review.
- Treatment compliance check.
- Concomitant medications.
- Adverse events.
- Weight in Kg.
- Physical examination.
- Blood results review.
- IBDQ & EQ-5D patient rated quality of life measure.
- Resource usage patient questionnaire.
- Primary outcomes review (steroids and surgery).

Samples to be collected and processed **locally:**

- Full blood count.
- Biochemical series (including urea, creatinine, electrolytes, liver function tests, CRP), [thiopurine metabolites if taking Azathioprine or 6-Mercaptopurine].

Samples to be collected and sent, to be processed **centrally:**

- PAXgene tube.
- Serum tube.
- Stool sample for faecal Calprotectin.

<u>Week 48 (end of trial visit) (±4wks)</u> Patient data to collect:

- HBI.

- Perianal disease review.
- Treatment compliance check.
- Concomitant medications.
- Adverse events.
- Weight in kg.
- Physical examination.
- Blood results review.
- IBDQ & EQ-5D patient rated quality of life measures.
- Resource usage patient questionnaire.
- Endoscopic results & video (can be performed up to 4 weeks after week 48).
- MRE (can be performed up to 6 weeks after week 48).
- Primary outcomes review (steroids and surgery).

Samples to be collected and processed **locally:**

- Full blood count.
- Biochemical series (including urea, creatinine, electrolytes, liver function tests), CRP), [thiopurine metabolites if taking Azathioprine or 6-Mercaptopurine].

Samples to be collected and sent, to be processed **centrally:**

- PAXgene tube.
- Serum tube.
- Buffered stool sample.
- Stool sample for faecal Calprotectin.

Ad hoc visits (for disease flares in between scheduled visits and issues with trial treatment)

Patient data to collect:

- HBI.
- Perianal disease review.
- Treatment compliance check.
- Concomitant medications.
- Adverse events.
- Weight in kg.
- Physical examination.

- Blood results review.
- Primary outcomes review (steroids and surgery).

Samples to be collected and processed **locally:**

- Full blood count.
- Biochemical series (including urea, creatinine, electrolytes, liver function tests, CRP), [thiopurine metabolites if taking Azathioprine or 6-Mercaptopurine]. Stool for microscopy, culture and sensitivity.

If the participant is meeting the definition of a flare [HBI ≥ 7 (symptoms for approximately 7 days*) AND [CRP ≥ upper limit of normal on local assay OR Calprotectin ≥ 200 µg/g] AND [Clinical opinion that symptoms are attributable to a flare of Crohn's disease] *persisting symptoms present for (typically) 7 days or more (recognising that a true flare may be shorter in duration in some instances), research samples need to be collected and sent to be processed **centrally:**

- PAXgene tube.
- Faecal Calprotectin.

<u>Concomitant medications</u>

Treatments used as part of the trial protocol, including medications co-prescribed with trial medications e.g. vitamin D and calcium combinations alongside steroids, or medications to manage infusion reactions e.g. chlorphenamine/hydrocortisone, do not need to be recorded on the concomitant medications form.

10.4 End of Trial Participation

Following the end of trial visit, participants will return to the normal standard of care as defined by their local physicians. The costs of this standard care will continue under local funding and commissioning arrangements, according to local practice.

10.5 Schedule of Events

Baseline visit should be 2 weeks +10 days from screening. Week 4 visit should be within -10 days or +4 weeks of the schedule. Week 16 to 48 visits should be within ± 4 weeks of the schedule.

Participants in the "Top-Down" group should be started on Infliximab 2 weeks after randomisation (± 7 days), Following first infusion, subsequent infusions will be at 2 weeks after (± 7 days), then 6 weeks after the first infusion (± 7 days), and then infused 8 weekly thereafter (± 7 days), i.e. standard induction and treatment regimen for Infliximab i.e. standard induction and treatment regimen for Infliximab. Dates of Infliximab infusions should be aligned to trial visits in order to minimise hospital attendance for participants.

Procedure	Screening Wk -2	Baseline Wk 0	Wk 4	Wk 16	Wk 32	Wk 48	Ad hoc
Written consent	✓						
Demographics (DOB, gender, initials, ethnicity)	✓						
Medical History	✓	✓					
Disease assessment - Harvey Bradshaw Index (HBI)	✓	✓	✓	✓	✓	✓	✓
Perianal disease review	✓	✓	✓	✓	✓	✓	✓
Concomitant medication	✓	✓	✓	✓	✓	✓	✓
AE reporting		✓	✓	✓	✓	✓	✓
Weight in Kg	✓	✓	✓	✓	✓	✓	✓
Height in cm	✓						
Physical examination	✓	✓	✓	✓	✓	✓	✓
Eligibility confirmed		✓					
Randomisation		✓					
PAXgene RNA tube for biomarker assessment	✓						
PAXgene RNA tube for research sample	✓	✓		✓	✓	✓	✓
Serum tube	✓			✓	✓	✓	
EDTA tube	✓						
Bloods (FBC, CRP, U&E, Creatinine, LFT)	✓†	✓	✓	✓	✓	✓	✓
Blood results review	✓	✓	✓	✓	✓	✓	✓
Screening (Hepatitis B & C, VZV, TPMT)	✓						
Assessment of TB	✓						

Pregnancy test	✓						
Bloods (thiopurine metabolites)			✓[α]	✓[α]	✓[α]	✓[α]	✓[α]
Faecal Calprotectin	✓[†]	†		✓	✓	✓	✓[β]
Buffered stool sample	✓					✓	
Stool sample for mc&s							✓
Primary outcomes review (steroids and surgery)			✓	✓	✓	✓	✓
IBDQ & EQ-5D Qol	✓	✓		✓	✓	✓	
Resource usage questionnaire		✓		✓	✓	✓	
Endoscopy	✓*					✓	
MR enterography MRE	✓¥					✓	

† Results of blood tests e.g. CRP and faecal Calprotectin performed as part of standard care can be used for eligibility provided performed up to 3 months prior to the baseline visit – however a repeat stool sample should still be sent for central processing (as Central laboratory results will be used in subsequent analyses). Sample pot for faecal Calprotectin will be provided at screening visit and patients advised to provide sample promptly so that central Calprotectin result is available at the baseline visit.

α 6-TGN and 6-MMP metabolites to be measured at every scheduled trial visit only if participants are taking either Azathioprine or 6-Mercaptopurine.

B Sample for faecal Calprotectin and paxgene blood should be collected at *ad-hoc* visit for delivery to central trial team, for any instances where treatment is escalated for active Crohn's disease.

* Screening endoscopy results can be taken from the participants' index colonoscopy or capsule endoscopy or flexible sigmoidoscopy performed as part of their standard care within 6 months of the screening visit, although preferably a video of the colonoscopy should be recorded for central reading to allow comparison with the colonoscopy at week 48.

¥ It is anticipated that all patients newly diagnosed with Crohn's disease will undergo MRE. An MRE performed as part of standard of care within 6 months of screening can be used; and if not already performed should be undertaken within 3 months of the screening visit. The results of this index MRE will be collected at the week 16 visit. It will be used for central reading and allow comparison with MRE at week 48.

10.6 Trial restrictions

There are no dietary restrictions in the trial.

If Methotrexate or Allopurinol are part of treatment, women of childbearing potential are required to use adequate contraception for the duration of the trial and for 6 months after the completion of the trial visit/last treatment. These can include:

- Intrauterine Device (IUD).
- Hormonal based contraception (pill, contraceptive injection or implant etc.).
- Barrier contraception (condom or occlusive cap e.g. diaphragm or cervical cap with spermicide).
- True abstinence (where this is in accordance with the patients' preferred and usual lifestyle).

Males on Methotrexate are required to use barrier contraception for the duration of the trial and for 6 months after the completion of the trial/last treatment. Males on Methotrexate treatment should also refrain from donating sperm for the duration of the trial and for 6 months after completion of the trial/last treatment.

The SmPC indicates that women of child bearing potential receiving anti-TNF should consider the use of adequate contraception to prevent pregnancy and continue its use for at least 6 months after the last infliximab treatment and for at least 5 months after the last adalimumab treatment.

11 Assessment of Safety

11.1 Definitions

11.1.1 Adverse event (AE)

Any untoward medical occurrence in a patient or clinical trial participant administered a treatment and which does not necessarily have a causal relationship with this treatment.

An adverse event can therefore be any unfavourable and unintended sign (including an abnormal laboratory finding), symptom, or disease temporally associated with the use of a treatment, whether or not considered related to the treatment.

For the purpose of the trial, only AEs that are related to Crohn's disease (disease flares or surgery) or drug therapy for Crohn's disease (sufficiently severe to require a switch to an alternative treatment), or the associated biomarker sample collection will be recorded and assessed.

Serious infections (requiring hospital admission) should be recorded as an AE, regardless of which treatment arm the participant has been randomised to. Infections which do not require hospital admission do not need to be recorded as AEs for purposes of the PROFILE trial. In addition, AEs related to drug therapy in particular should include the following, if taking the relevant medication:

Thiopurines (Azathiopurine or 6-Mercaptopurine)

- Pancreatitis (confirmed with either abdominal scan or amylase/lipase >2x upper limit of normal on local laboratory assay).
- Myelosuppression (WCC <2.0 10^9/L or neutrophils <1.0 10^9/L).
- Liver toxicity (transaminases >4x upper limit of normal on local laboratory assay).
- Lymphoma.
- Skin cancer.

Methotrexate

- Pneumonitis.
- Myelosuppression (WCC <2.0 10^9/L or neutrophils <1.0 10^9/L).
- Liver toxicity (transaminases >4x upper limit of normal). [illegible] Pregnancy.

Anti-TNF (Infliximab or Adalimumab)

- Demyelination.
- Active tuberculosis.
- Sepsis (requiring hospitalisation).
- Malignancy.
- Anaphylaxis.

Please note: Recording of all adverse events must start from the point of Informed Consent regardless of whether a participant has yet received treatment.

11.1.2 Serious adverse event (SAE)

Any of the above adverse events that reach the criteria of serious should be recorded as serious adverse events.

The serious criteria are defined as:

- Results in death.
- Is life-threatening.
- Requires hospitalisation or prolongation of existing inpatients´ hospitalisation.
- Results in persistent or significant disability or incapacity.
- Results in a congenital anomaly or birth defect.
- Is an important medical event - Some medical events may jeopardise the participant or may require an intervention to prevent one of the above characteristics/ consequences. Such events should also be considered as 'serious'.

Any deaths during the trial regardless of cause should be recorded using a serious adverse event form.

Life-threatening in the definition of a serious adverse event or serious adverse reaction refers to an event in which the participant was at risk of death at the time of event; it does not refer to an event which hypothetically might have caused death if it were more severe.

11.2 Evaluation of adverse events

The Sponsor expects that adverse events are recorded from the point of Informed Consent regardless of whether a patient has yet received a medicinal product. Individual adverse events should be evaluated by the Investigator. This includes the evaluation of its seriousness, causality and any relationship between drug therapy and/or concomitant therapy and the adverse event.

11.2.1 Assessment of seriousness

Seriousness is assessed against the criteria in section 11.1.2. This defines whether the event is an adverse event or a serious adverse event.

11.2.2 Clinical assessment of severity

Mild: The participant is aware of the event or symptom, but the event or symptom is easily tolerated.

Moderate: The participant experiences sufficient discomfort to interfere with or reduce his or her usual level of activity.

Severe: Significant impairment of functioning; the participant is unable to carry out usual activities and / or the participant's life is at risk from the event.

11.2.3 Recording of adverse events

Adverse events and adverse reactions should be recorded in the medical notes and the appropriate section of the CRF and/or AE log. Serious Adverse Events should be reported to the sponsor as detailed in section 11.3.

11.3 Reporting serious adverse events

Each Principal Investigator needs to record all adverse events and report serious adverse events to the Chief Investigator using the trial specific SAE form within 24 hours of their awareness of the event. The Chief Investigator is responsible for ensuring the assessment of all SAEs for expectedness and relatedness is completed and the onward notification of all SAEs to the Sponsor immediately but not more than 24 hours from first notification. The sponsor has to keep detailed records of all SAEs reported to them by the trial team.

The Chief Investigator is also responsible for prompt reporting of all serious adverse event findings to the competent authority if they could:

- Adversely affect the health of participants.
- Impact on the conduct of the trial.
- Alter the risk to benefit ratio of the trial.

The completed SAE form should be emailed to add-tr.profile@nhs.net

11.4 Pregnancy Reporting

All pregnancies within the trial (for trial participants) should be reported to the Chief Investigator and the Sponsor using the relevant Pregnancy Reporting Form within 24 hours of notification. Pregnancy reporting should stop 6 months after the patient's last dose of trial medications.

IBD patients receiving anti-TNF therapy should be counselled about the risks and benefits of continuing treatment throughout pregnancy.

For patients with active disease or a high risk of relapse, it may be advisable to continue anti-TNF agents throughout pregnancy.

If there is no significant increase in the risk of disease flare, anti-TNF agents can be discontinued during the later stages of pregnancy to minimize exposure to the foetus.

If anti-TNF agents are to be discontinued, then this should only take place after discussion with the Chief Investigator.

Pregnancy is not considered an AE unless a negative or consequential outcome is recorded for the mother or child/foetus. If the outcome meets the serious criteria, this would be considered an SAE.

12 Toxicity – Emergency Procedures

In the event of toxicity reactions, standard medical practices will be employed, including emergency care and hospitalisation if needed. All medications in this trial are licensed for use in Crohn's disease and their uses are familiar to the physicians and staff at all participating centres.

13 Evaluation of Results (Definitions and response/evaluation of outcome

measures)

The unblinded data will be presented to the Data Monitoring Committee, who will meet on a regular basis throughout the trial. The DMC will then prepare a report for the Trial Steering Committee who will provide overall supervision of the trial.

13.1 Response criteria

13.1.1 Steroid free remission

Steroid use will be measured following the initial induction course of steroids until week 48 from randomisation and will then be recorded at all trial time points. This specifically relates to a course of systemic steroid medication for treatment of active Crohn's disease.

13.1.2 Surgery free remission

All Crohn's disease related surgeries will be recorded from the date of randomisation and will be recorded at all trial time points.

13.1.3 Quality of life

Patient rated Quality of Life (QoL) measure (IDBQ & EQ-5D) will be recorded from the date of randomisation and at a variety of trial related time points.

14 Storage and Analysis of Samples

Stool samples are to be shipped to Addenbrooke's Hospital (Department of Medicine, University of Cambridge, Cambridge Biomedical Campus) for analysis of faecal Calprotectin and storage for future research into the microbiome. Samples can be stored at room temperature for transport and are stable for 7 days. On receipt samples will be stored at approximately -70C (or -80°C depending on the freezer) prior to processing and analysis.

Screening and weeks' 16, 32 and 48 serum samples (Pearl white-top plasma preparation tube) and screening blood sample (red-top EDTA preparation tube) are to be shipped to Addenbrooke's Hospital (Department of Medicine, University of Cambridge, Cambridge Biomedical Campus) for storage and subsequent analysis.

Samples are stable for 24-48 hours at room temperature. On receipt samples will be stored in a freezer with temperature controlled at approximately -70C (or -80°C depending on the freezer). Samples will be analysed for gene expression, proteins and metabolites including drug and antibody levels.

PAXgene blood RNA tubes are to be shipped to Addenbrooke's Hospital (Department of Medicine, University of Cambridge, Cambridge Biomedical Campus) for analysis. Samples are stable at room temperature for up to 3 days. On receipt samples will be logged and stored in a fridge (approximately 2-8°C) prior to processing. Samples stored in the fridge are stable for 5 days. Whole blood RNA will be extracted.

An aliquot of total RNA will be converted to cDNA for analysis, the remaining RNA and any residual cDNA will be stored in an approximately -70C (or -80°C depending on the freezer).

All blood and stool samples and materials derived from them will be stored anonymously, labelled with the unique trial number assigned to each participant. Trial samples and clinical outcomes data gathered as part of the trial will be linked via the unique trial number, to enable their use in future exploratory translational and scientific studies. These will include correlating clinical outcomes with microbial, metabolomic, proteomic, genetic and gene expression signatures and potentially the development of new biomarkers.

15 Statistics

15.1 Statistical methods

The primary outcome, relapse-free incidence, will be analysed with logistic regression using an additive link function to estimate the absolute relapse-free rate differences with main and interaction effects for treatment and biomarker, and adjusting for baseline variables:

- Smoking status.
- Disease location (Ileal, Colonic, Ileocolonic).
- Age.
- CRP.
- Calprotectin.
- Azathioprine / 6-Mercaptopurine vs Methotrexate.
- Course of steroids prior to trial enrolment (yes/no).

The primary comparison of interest is the additive interaction between treatment and biomarker: IBD^{hi}:("Accelerated Step-Up" - "Top-Down") – IBD^{lo}:("Accelerated Step-Up" - "Top-Down"). This effect will be estimated, with 95% confidence intervals and tested at a 2-sided 5% significance level. In general the key comparison of interest for all endpoints is the treatment-biomarker interaction, and not the main effects.

All comparisons (main effects, interactions, covariate adjustments) will be reported with estimates, 95% confidence intervals and p-values. However to formally control for multiple testing, we will perform a closed testing procedure over the primary and five secondary endpoints (see section 7.4.2) testing the biomarker-treatment interaction and restricting the family-wise type 1 error rate to an overall 5% significance level.

The methodology to combine together gate-keeping and Holm-Bonferroni methods in formal hypothesis testing will be used (Bretz et al. Stat Med 2009), with the diagram below defining how the significance levels will be transitioned assuming an initial configuration of 5% at the primary endpoint (relapse-free remission) and 0% on all other tests.

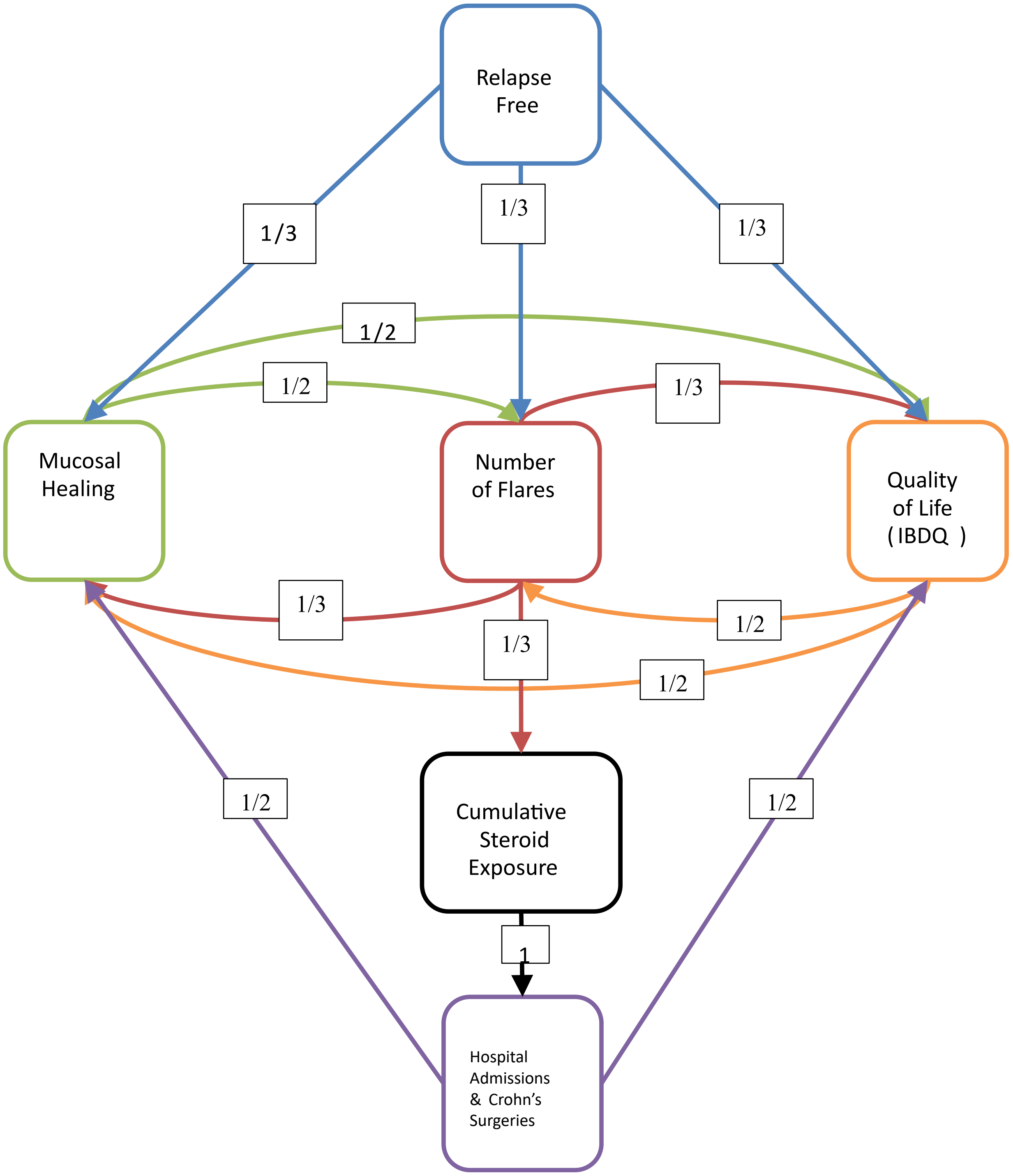

The diagram shows the flow of significance level spending in the sequential multiple testing procedure.

Mucosal healing is a binary endpoint and will be analysed in a similar fashion to the primary endpoint. All the remaining secondary endpoints are continuous variables and will be analysed using a linear regression framework adjusting for baseline covariates.

Quality of life is observed over repeated visits and will be analysed using a mixed effect repeat measure analysis with a clustered patient-level residual error with unstructured covariance over visits, fixed effects for visit, and all other covariates assumed to have a constant fixed effect over time.

Summary statistics will be provided broken down by treatment arm, pooled and stratified across biomarker subgroup. Continuous variables will summarised by n, mean, median, SD, min, max; categorical or binary will be summarised by frequency (x/n) and percentage (%). Graphical counterparts will be provided: box and whisker plots for continuous variables, and stacked bar charts for categorical and binary variables.

15.2 Health Economics

A full health economic analysis will be performed; the detailed analysis will be described in the health economic analysis plan.

15.3 Interim analyses

No interim analyses will be performed. The DMC (unblinded) will review efficacy and safety data and will advise on the need for any additional analyses or alterations to the conduct or even continuation of the trial.

15.4 Number of participants to be enrolled

Remission rates were estimated using data pertaining to the clinical phenotype of IBD^{hi} and IBD^{lo} patients (see Appendix) and data from the literature regarding response to early anti-TNFα (including the original "Step Up-Top Down" study, D'Haens et al. 2008 Lancet 371:660-667, the SONIC trial Colombel et al. 2010 NEJM 362:1383-1395, and subgroup analyses of large anti-TNFα trials D'Haens. 2010 Nat Rev Gastroent Hepatol 7:86-92).

Estimated remission rates were: IBD^{hi}: "Accelerated Step-Up" 0.3, "Top-Down" 0.7; IBD^{lo}: "Accelerated Step-Up" 0.8, "Top-Down" 0.9. The prevalence of IBD^{hi}/ IBD^{lo} is 0.5/0.5 based on all of the cohorts in whom the classifier has been assessed. The primary comparison is powered as an interaction analysis, where the interaction refers to the difference between the relative benefits of "Top-Down" over "Accelerated StepUp" in each subgroup. Based on the remission rates and subgroup prevalence rates above, an interaction of 0.3 can be detected with a power of 92% at a 2-sided 5% significance level with a total sample size of 333.

To allow for a ~17% drop out rate, 400 patients will be recruited across approximately 50 sites. This requires recruitment of 5 patients per site per year, which is a conservative estimate based on previous recruitment to Investigator-led UK studies. Recruitment will take place over 2 years.

15.5 Criteria for the premature termination of the trial

There are no defined criteria for the premature discontinuation of the trial. However the DMC and TSC will make recommendations on the discontinuation of the trial following review of the on-going patient safety and efficacy data presented at regular scheduled meetings.

15.6 Procedure to account for missing or spurious data

For the primary analysis missing data will be assumed to be missing at random.

15.7 Definition of the end of the trial

The end of trial will be the last patient's last trial visit.

16 Data handling and record keeping

16.1 CRF

All data will be transferred into a Case Report Form (CRF), which will be pseudoanonymised. All trial data in the CRF must be extracted from and be consistent with the relevant source documents. The CRFs must be completed, dated and signed by the Investigator or designee in a timely manner. It remains the responsibility of the Investigator for the timing, completeness, legibility and accuracy of the CRF pages. The CRF will be accessible to trial coordinators, data managers, the Investigators, Clinical Trial Monitors, Auditors and Inspectors as required.

Scanned copies of the CRFs should be emailed to add-tr.profile@nhs.net according to the agreed timelines detailed in the CRF Completion Guide.

The Investigator will retain the original of each completed CRF page at site. The Investigator will also supply the trial coordination centre with any required, anonymised background information from the medical records as required.

All CRF pages must be clear and legible. Any errors should be crossed through with a single stroke so that the original entry can still be seen. Corrections should be inserted and the change dated and initialled by the Investigator or designee. If it is not clear why the change has been made, an explanation should be written next to the change. Typing correction fluid must not be used.

16.2 Source Data

To enable peer review, monitoring, audit and/or inspection, the Investigator must agree to keep records of all participants (sufficient information to link records e.g. CRFs, hospital records and samples), all original signed informed consent forms and copies of the CRF pages.

CRF pages are not considered to be source data.

Source data is considered to be the first recording of trial related information. Source data/documents can include but not exclusive to; patient medical records, patient trial notes, patient electronic medical records (including blood test results), signed informed consent forms, screening logs, patient rated QoL measures and sample logs. This list is not exhaustive.

Any data collection tools used for the purpose of aiding data collection are not considered to be source data.

16.3 Endoscopic Video and MRE Images

Endoscopic video recordings / images and MRE images to be supplied anonymised and linked by trial number only.

16.4 Data Protection & Patient Confidentiality

All Investigators and trial site staff involved in this trial must comply with the requirements of the Data Protection Act 1998 and Trust Policy with regards to the collection, storage, processing and disclosure of personal information and will uphold the Act's core principles.

17 Data Monitoring Committee/Trial Steering Committee

The TSC will provide overall supervision with respect to the conduct of the trial. The independent chair of the TSC will be Geert D'Haens (Amsterdam Medical Centre, Amsterdam, Netherlands). Full details of the TSC membership and remit can be found in the TSC charter.

The ethical and safety aspects of the trial will be overseen by an independent DMC. The independent chair of the DMC will be Vipul Jairath (University Hospital, Ontario, Canada). DMC meetings will be timed so that reports can be fed into the TSC meetings. Full details of the DMC membership and remit can be found in the DMC Charter.

18 Ethical & Regulatory considerations

18.1 Consent

The Informed Consent form must be approved by the REC and must be in compliance with GCP, local regulatory requirements and legal requirements. The Investigator must ensure that each trial participant, or his/her legally acceptable representative, is fully informed about the nature and objectives of the trial and possible risks associated with their participation.

The Investigator will obtain written informed consent from each patient before any trialspecific activity is performed. The informed consent form used for this trial and any change made during the course of this trial, must be prospectively approved by the REC. The Investigator will retain the original of each patients signed informed consent form.

Should a participant require a verbal translation of the trial documentation by a locally approved interpreter/translator, it is the responsibility of the individual Investigator to use locally approved translators.

Any new information that becomes available, which might affect the participant's willingness to continue in the trial will be communicated to the participant as soon as possible.

18.2 Ethical committee review

Before the start of the trial or implementation of any amendment we will obtain approval of the trial protocol, protocol amendments, informed consent forms and other relevant documents e.g., advertisements and GP information letters if applicable from the REC. All correspondence with the REC will be retained in the Trial Master File/Investigator Site File.

Annual reports will be submitted to the REC in accordance with national requirements. It is the Chief Investigator's responsibility to produce the annual reports as required.

18.3 Regulatory Compliance

This is not an investigation of medicinal products.

18.4 Protocol Amendments

Protocol amendments must be reviewed and agreement received from the Sponsor for all proposed amendments prior to submission to the REC.

The only circumstance in which an amendment may be initiated prior to REC approval is where the change is necessary to eliminate apparent, immediate risks to the patients (Urgent Safety Measures). In this case, accrual of new patients will be halted until the REC approval has been obtained.

18.5 Peer Review

The trial proposal has been peer-reviewed and is supported by the Wellcome Trust as part of the translational funding award process.

The trial protocol was peer reviewed by the British Society of Gastroenterology IBD Clinical Research Group (BSG IBD CRG).

18.6 Declaration of Helsinki and Good Clinical Practice

The trial will be performed in accordance with the spirit and the letter of the declaration of Helsinki, the conditions and principles of Good Clinical Practice, the protocol and applicable local regulatory requirements and laws.

19 Sponsorship, Financial and Insurance

The trial is sponsored by Cambridge University Hospitals NHS Foundation Trust and the University of Cambridge. The trial is funded by the Wellcome trust.

Cambridge University Hospitals NHS Foundation Trust, as a member of the NHS Clinical Negligence Scheme for Trusts, will accept full financial liability for harm caused to participants in the clinical trial caused through the negligence of its employees and honorary contract holders. There are no specific arrangements for compensation should a participant be harmed through participation in the trial, but no-one has acted negligently.

The trial Sponsors will arrange insurance for negligent harm caused as a result of protocol design and for non-negligent harm arising through participation in the clinical trial.

Infliximab for trial participants will be supplied to hospitals via a trial specific ordering form. If participants move to Adalimumab, the cost of this can be reimbursed by the sponsor on receipt of quarterly invoices.

Travel, parking and any other reasonable costs to attend trial visits (up to £240 total/£40 per visit for the duration for the trial) can be reimbursed for participants. Participating trusts can request reimbursement on a quarterly basis from the trial sponsor.

20 Monitoring, Audit & Inspection

Should a monitoring visit or audit be requested, the Investigator must make the trial documentation and source data available to the Sponsor's representative. All patient data must be handled and treated confidentially.

The Sponsor's monitoring frequency will be determined by an initial risk assessment performed prior to the start of the trial. A monitoring plan will be generated detailing the frequency and scope of the monitoring for the trial. All participating sites will be subject to routine trial specific on-site monitoring/remote monitoring.

21 Protocol Compliance and Breaches of GCP

Prospective, planned deviations or waivers to the protocol are not allowed under the UK and European regulations on Clinical Trials and must not be used.

For example, it is not acceptable to enrol a participant if they do not meet one or more eligibility criteria or restrictions specified in the trial protocol. In the event that eligibility criteria need to be changed/amended then they MUST first be approved by REC via a substantial protocol amendment before they can be implemented.

Protocol deviations, non-compliances, or breaches will be documented on the relevant forms and reported to the Chief Investigator and Sponsor immediately.

Deviations from the protocol which are found to recur constantly will be addressed with immediate action.

Any potential/suspected serious breaches of GCP will be reported immediately to the Sponsor without any delay.

22 Publications policy

Ownership of the data arising from this trial resides with the trial team (comprising the Chief Investigator, Co-Investigators, Chief Technical Officers). On completion of the trial the data will be analysed and tabulated and a Final Trial Report prepared.

Participating Investigators do not have rights to publish any trial data.

This is a Wellcome Trust funded trial (translational funding grant) and the Wellcome Trust publishing guidelines will be followed.

Following completion of the Final Trial Report and publication of results, participants in the trial can request trial results from their Principal Investigator and they can be provided with a link of publicly available manuscripts.

23 References

1. Bretz, F., Maurer, W., Brannath, W., Posch, M. A graphical approach to sequentially rejective multiple test procedures. Stat Med. 28, 586–604 (2009).

2. Colombel, J.F., Sandborn, W.J., Reinisch, W., Mantzaris, G.J., Kornbluth, A., Rachmilewitz, D., Lichtiger, S., D'Haens, G., Diamond, R.H., Broussard, D.L., Tang, K.L., van der Woude, C.J. & Rutgeerts, P. Infliximab, azathioprine, or combination therapy for Crohn's disease. N Engl J Med 362, 1383-1395 (2010).

3. Cosnes, J., Bourrier, A., Laharie, D., Nahon, S., Bouhnik, Y., Carbonnel, F., Allez, M., Dupas, J.L., Reimund, J.M., Savoye, G., Moreau, J., Mary, J.Y., Colombel, F.F., Groupe d'Etude Therapetiques des Affections INflammatoires due Tube Digestif (GETAID). Early administration of azathioprine vs conventional management of Crohn's Disease: a randomized controlled trial. Gastroenterology 145, 758-765 (2013).

4. D'Haens, G., Baert, F., van Assche, G., Caenepeel, P., Vergauwe, P., Tuynman, H., De Vos, M., van Deventer, S., Stitt, L., Donner, A., Vermeire, S., Van de Mierop, F.J., Coche, J.C., van der Woude, J., Ochsenkuhn, T., van Bodegraven, A.A., Van Hootegem, P.P., Lambrecht, G.L., Mana, F., Rutgeerts, P., Feagan, B.G. & Hommes, D. Early combined immunosuppression or conventional management in patients with newly diagnosed Crohn's disease: an open randomized trial. Lancet 371, 660-667 (2008).

5. Freidlin, B., McShane, L.M. & Korn, E.L. Randomized clinical trials with biomarkers: design issues. J Natl Cancer Inst 102, 152-160 (2010).

6. Khanna, R., Bressler, B., Leveseque, B.G., Zou, G., Stitt, L.W., Greenberg, G.R., Panaccione, R., Bitton, A., Paré, P., Vermeire, S., D'Haens, G., MacIntosh, D., Sandborn, W.J., Donner, A., Vandervoort, M.K., Morris, J.C., Feagan, B.G., REACT Study Investigators. Early combined immunosuppression for the management of Crohn's disease (REACT): a cluster randomised controlled trial. Lancet 386, 1825-1834 (2015).

7. Lee, J.C., Lyons, P.A., McKinney, E.F., Sowerby, J.M., Carr, E.J., Bredin, F., Rickman, H.M., Ratlamwala, H., Hatton, A., Rayner, T.F., Parkes, M. & Smith, K.G.C. Gene expression profiling of CD8+ T cells predicts prognosis in patients with Crohn disease and ulcerative colitis. J Clin Invest 121, 4170-4179 (2011).

8. McKinney, E.F., Lyons, P.A., Carr, E.J., Hollis, J.L., Jayne, D.R., Willcocks, L.C., Koukoulaki, M., Brazma, A., Jovanovic, V., Kemeny, D.M., Pollard, A.J., Macary, P.A., Chaudhry, A.N. & Smith, K.G. A CD8+ T cell transcription signature predicts prognosis in autoimmune disease. Nat Med 16, 586-591, 581p following 591 (2010).

9. Panes, J., Lopez-Sanroman, A., Bermejo, F., Garcia-Sanchez, V., Esteve, M., Torres, Y., Domenech, E., Piqueras, M., Gomez-Garcia, M., Gutierrez, A., Taxonera, A., Sans, M., AZTEC

Study Group. Early azathioprine therapy is no more effective than placebo for newly diagnosed Crohn's disease. Gastroenterology 145, 766-774 (2013).

10. Potti, A., Mukherjee, S., Petersen, R., Dressman, H.K., Bild, A., Koontz, J., Kratzke, R., Watson, M.A., Kelley, M., Ginsburg, G.S., West, M., Harpole, D.H., Jr. & Nevins, J.R. A genomic strategy to refine prognosis in early-stage nonsmall-cell lung cancer. N Engl J Med 355, 570-580 (2006).

11. Smith, M.A., Blaker, P., Marinaki, A.M., Anderson, S.H., Irving, P.M., Sanderson, J.D. Optimising outcome on thiopurines in inflammatory bowel disease by coprescription of allopurinol. J Crohns Colitis. 6, 905-12 (2012).

24 Appendices

24.1 Appendix 1 - Trial Management / Responsibilities

24.1.1 Patient registration/ Randomisation procedure

Patients will be randomised using a stratified block randomisation technique via a web-based system.

24.1.2 CRF Completion & Data management

All CRFs should be completed in a timely manner. Data management at the Trial

Coordinating Centre will be undertaken by the trial coordinating team. The local Principal Investigators will be responsible for overseeing the collection of accurate data at their participating site. The completed CRFs will be signed by either the Principal Investigator or a suitably qualified and delegated member of their trial team. Data management will entail the checking of the CRFs and ensuring that data queries are completed in a timely manner in accordance with the data management plan.

24.1.3 Preparation & submission of amendments

Amendments to the trial will be prepared and submitted to the appropriate authorities, by a member of the Trial Coordinating team. Subsequent approvals will then be disseminated to all sites, prior to implementation.

24.1.4 Preparation and submission of Annual Safety Report/Annual Progress Reports

A member of the Trial Coordinating team will prepare the ASR and APR reports prior to submission.

24.1.5 Trial Monitoring

The frequency, scope and method of monitoring will be determined by the Sponsor's trial level risk assessment. All sites will be monitored in accordance with the monitoring plan.

24.1.6 Data protection/ confidentiality

All investigators and trial site staff involved in this trial must comply with the requirements of the Data Protection Act 1998 and Trust policy with regards to collection, storage, processing and disclosure of personal information and will uphold the Act's core principles.

24.1.7 Trial documentation & archiving

All trial documentation will be retained in a secure location during the conduct of the trial and following end of trial, archived in accordance with the Sponsors SOPs. Each site will be responsible for archiving their Investigator Site File and associated trial documentation.

24.2 Appendix 2 – Authorisation of Participating Sites

24.2.1 Required Documentation

Prior to initiating a participating site the following documentation is required;

- Principal Investigator and other key trial team staff CV (signed and dated) and GCP certificate
- Competent Authority approval (HRA)

- Local R & D compliance and capacity approval
- Participating Site Agreement executed, including pharmacy participating site agreement
- Patient Information sheets and consent forms on local headed paper
- Protocol signed and dated by Principal Investigator
- Delegation of Authority Log
- Confirmation of randomisation system training

24.2.2 Procedure for initiating/opening a new site

When all the regulatory paperwork is in place, prior to site opening, an initiation meeting will take place, either face to face or via a teleconference. This will be led by a member of the Trial Coordinating team with as many of the local team present as is practicable. This initiation meeting constitutes training for the trial and it is therefore imperative that all members of the trial team who will be involved in the trial are represented at the meeting. A log of attendees will be completed during the meeting. The presentation slides will be provided to the site in advance of the meeting. A trial initiation form will be completed for each site initiation meeting. Copies of all initiation documentation must be retained in the Investigator Site File.

The sponsor's regulatory green light procedure will be followed. Following the green light, the initial supply of IMP will be ordered for shipment to the site on the authorisation of the coordinating centre coordinator. Following confirmation of receipt of the IMP at site, the site will be opened for recruitment and the randomisation system opened to that site.

24.2.3 Principal Investigator Responsibilities

The Principal Investigator has overall responsibility for the conduct of the trial at the participating site.

In particular, the Principal Investigator has responsibilities, which include (but are not limited to):

- Ensuring the appropriate approvals are sought and obtained.
- Continuing oversight of the trial.
- Ensuring the trial is conducted according to the protocol.
- Ensuring consent is obtained in accordance with the protocol and national requirements.
- Ensuring that the ISF is accurately maintained.
- Delegation of activities to appropriately trained staff (this must be documented on the delegation of authority log).
- Providing protocol or specialised training to new members of the trial team and ensuring that if tasks are delegated, the member of staff is appropriately trained and qualified.
- Appropriate attendance at the initiation meeting.

- Ensuring appropriate attendance at the TSC/IDMC meetings if required and ensuring appropriate safety information is made available to the coordinating centre team in advance of the meetings.
- Dissemination of important safety or trial related information to all stakeholders at the participating site.
- Safety reporting within the timelines and assessment of causality and expectedness of all SAEs.

24.3 Safety Reporting Flow Chart

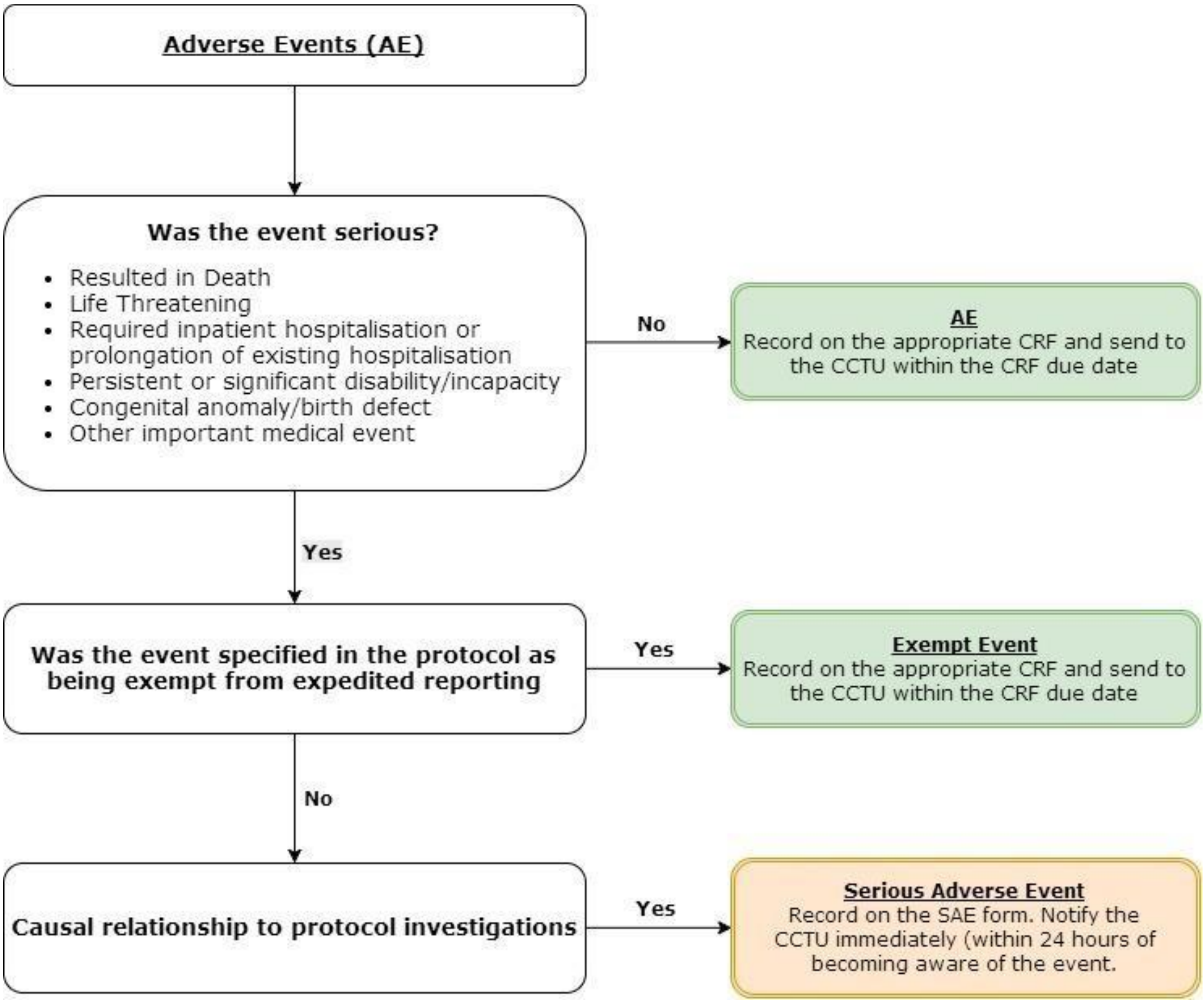

PROFILE Trial Statistical Analysis Plan.

Title: PROFILE Trial Statistical Analysis Plan

Statistical Analysis Plan

TRIAL FULL TITLE	**PR**edicting **O**utcomes **F**or Crohn's d**I**sease using a mo**L**ecular biomark**E**r (PROFILE) trial
EUDRACT NUMBER	
SAP VERSION	3.0
ISRCTN NUMBER	ISRCTN11808228
SAP VERSION DATE	17 AUG 2023
TRIAL STATISTICIAN	Simon Bond
TRIAL CHIEF INVESTIGATOR	Miles Parkes
SAP AUTHOR	Simon Bond

SAP Signatures

I give my approval for the attached SAP entitled Profile Final Analysis Version 3.0 dated 17 AUG 2023

Chief Investigator

Name: Miles Parkes

Signature: ________________________

Date: ________________________

Statistician

Name: Simon Bond

Signature: ________________________

Date: ________________________

Table of Contents

1 SAP Signatures 64
2 Table of Contents 65
3 Abbreviations and Definitions 68
4 Introduction 69
4.1 Preface 69
4.2 Purpose of the analyses 69
5 Trial Objectives and Endpoints 70
5.1 Trial Objectives 70
5.2 Endpoints 70
5.2.1 Primary Endpoint 70
5.2.2 Secondary Endpoints 71
5.2.3 Tertiary endpoints 71
5.2.4 Exploratory Endpoints 73
5.2.5 Other samples collected during PROFILE 74
6 Trial Methods/ 74
6.1 General Trial Design and Plan 74
6.2 Inclusion-Exclusion Criteria and General Trial Population 74
6.3 Randomisation and Blinding 75
6.4 Trial Variables 75
Procedure 75
7 Sample Size 78
8 General Considerations 79
8.1 Timing of Analyses 79
8.2 Analysis Populations 79
8.3 Covariates and Subgroups 80
8.3.1 Post-Baseline Subgroups 81
8.3.2 Potential Data 82
8.3.3 Coding details 82
8.4 Missing Data 83
8.5 Multiple Testing 84

9 Summary of Trial Data 86
9.1 Participant Disposition **Error! Bookmark not defined.**
9.2 Derived variables 86
9.3 Protocol Deviations 87
9.4 Demographic and Baseline Variables 87
9.5 Concurrent Illnesses and Medical Conditions 88
9.6 Prior and Concurrent Medications 88
9.7 Treatment Compliance 88
10 Efficacy Analyses 89
10.1 Primary Efficacy Analysis 90
10.2 Secondary Efficacy Analyses 90
10.2.1 Mucosal Healing 90
10.2.2 Number of Flares 91
10.2.3 Quality of Life IBDQ (Inflammatory Bowel Disease Questionnaire) 91
10.2.4 Cumulative Steroid Exposure 91
10.2.5 Number of Hospital Admissions and Surgeries for Crohn's disease 91
10.2.6 HBI 91
10.2.7 Incidence of Surgery 92
10.2.8 Incidence of Steroid Use 92
10.3 COVID sensitivity analyses 92
10.3.1 Seemingly Unrelated Regression92
10.4 Subgroup Analyses 93
10.5 Tertiary and Exploratory Efficacy Analyses 93
11 Safety Analyses 94
11.1 Adverse Events 94
11.2 Pregnancies 94
12 Reporting Conventions 94
13 Technical Details 94
14 Summary of Changes to the Protocol or SAP 95
15 Quality Control 96
16 References 97

17 Listing of Tables, Listings and Figures 98

Abbreviations and Definitions

AE	Adverse Event
CRF	Case Report Form
CRP	C-reactive Protein
DMC	Data Monitoring Committee
DoB	Date of Birth
EMA	European Medicine Agency
EQ-5D	Five dimensional Euro Quality of Life score
HBI	Harvey Bradshaw Index
IBDQ	Irritable Bowel Disease Questionnaire
ICH	International Council for Harmonisation of Technical Requirements for Pharmaceuticals for Human Use
IMP	Investigational Medical Product
MRE	Magnetic Resonance Elastography
PI	Principle Investigator
PT	Preferred Term
QC	Quality Control
SAE	Serious Adverse Event
SAP	Statistical Analysis Plan
SE	Standard Error
SES-CD	Simple Endoscopic Score in Crohn's Disease
SOC	System Organ Class
TSC	Trial Steering Committee

Introduction

Preface

The hypothesis is that biomarker-driven stratification will facilitate personalised therapy in Crohn's disease, and will improve clinical care. It will do this through identification of a group of participants destined to develop a more severe, relapsing course and who will benefit from "Top-Down" therapy while protecting those participants destined to experience more indolent disease from the risks and side-effects of unnecessary immunosuppression.

This is a randomised, multi-centre, biomarker-stratified, open-label trial in participants newly diagnosed with Crohn's disease (enrolled within 6 months of diagnosis). Participants are randomised to either "Top-Down" or "Accelerated Step-Up" therapy.

Purpose of the analyses

These will be the final analyses that will be performed, based on the full and complete data set of the trial. They will assess the efficacy endpoints and safety, and estimate the interaction effect between biomarker and treatment.

Of note, the primary analysis study-wide will be focusing on the biomarker-treatment interaction and not the main effects. However, a key and complementary analysis will be examining the relative treatment and safety effects between "Accelerated Step-Up" and "Top-Down" therapy arms.

Trial Objectives and Endpoints

Trial Objectives

(ICH E3; 8.)

To demonstrate that a whole-blood prognostic biomarker can improve clinical outcomes, quality of life, and health resource allocation by facilitating the delivery of personalised therapy from diagnosis in Crohn’s disease.

Endpoints

(ICH E9; 2.2.2)

Primary Endpoint

Incidence of sustained surgery and steroid free remission from completion of a protocolised (maximum 8-week regimen) steroid induction treatment through to week 48.

Remission at each visit is a composite of two conditions

- HBI score <=4
- Absence of objective evidence of inflammation: both CRP<=ULN and calprotectin<200 μg/g. If both values are missing then the condition is deemed missing. If just one value is missing then it is assumed to be below the threshold.

If either or both conditions hold then the participant is in remission at the visit.

This is equivalent to HBI<=4 at all assessments, or if symptoms are present (HBI>=5) there is no objective evidence of inflammation (CRP<=ULN and calprotectin<200). Requirement for an extended induction or additional course of systemic steroids or surgery for active Crohn's disease would result in failure to meet primary outcome measure.

The outcome of each possible permutation of HBI + CRP + calprotectin results is given below

**HBI**	**CRP**	**calpro**	**Outcome**
<5	<=ULN	<=200	remission
<5	<=ULN	missing	remission
<5	missing	<=200	remission
<5	missing	missing	missing
<5	<=ULN	>200	remission
<5	>ULN	<=200	remission
<5	>ULN	>200	remission
<5	>ULN	missing	remission
<5	missing	>200	remission

>=5	<=ULN	<=200	remission
>=5	<=ULN	missing	remission
>=5	missing	<=200	remission
>=5	missing	missing	missing
>=5	<=ULN	>200	flare
>=5	>ULN	<=200	flare
>=5	>ULN	>200	flare
>=5	>ULN	missing	flare
>=5	missing	>200	flare

Secondary Endpoints

1. Endoscopic remission at week 48 (defined by absence of ulceration i.e. SES-CD ulcer subscore = 0). Centrally-read endoscopic scores will be used where available. Where these are not available locally-read scores will be used.

2. Quality of life averaged across weeks 16, 32 and 48 (using disease specific IBD-Q score).

3.i Number of flares requiring treatment escalation by week 48 primary follow-up period.* Determined from adjudication of CRF data.

3.ii Cumulative steroid exposure by week 48 primary follow-up period (defined by number of courses of steroids for active Crohn's disease). Determined from adjudication of CRF data.

3.iii Number of hospital admissions and surgeries related to Crohn's disease by week 48 primary follow-up period. The number of hospital admissions will be derived from the AE information; the number of surgeries is directly captured in the CRF. The two counts will simply be added together without attempting to identify coincident hospitalisation-surgery events.

*flares will be considered as protocol definition of flare indicating need for treatment escalation

Tertiary endpoints

- Incidence of sustained surgery and steroid free remission from completion of a standard (maximum 8-week regimen) steroid induction treatment through to week 48 (when remission defined using clinical parameters alone, HBI < 5).
- Clinical remission (defined as HBI < 5) at weeks 4,16, 32 and 48.
- Biochemical remission (defined as CRP ≤ ULN and calprotectin <200) at weeks 4,16,32 and 48.
- CRP response (comparison of mean CRP scores in each group) at weeks 4,16,32 and 48.
- Calprotectin response (comparison of mean calprotectin scores in each group) at weeks 4,16,32 and 48.

- Incidence of 2 or more treatment escalations for flares of Crohn’s disease.
- Time to event, time from baseline to first flare or need for surgery for Crohn’s disease, which may occur during the protocolised induction course of steroid medication.
- Time to event, time from baseline to second flare or need for surgery for Crohn’s disease.
- Time to event, time from baseline to starting on anti-TNF therapy for Crohn’s disease.
- Patient reported clinical remission (using score generated from abdominal pain and stool frequency components of HBI score – abdominal pain ≤1 (none or mild) and stool frequency ≤3) at weeks 4,16,32,48.
- Steroid free clinical remission (defined as HBI < 5 and no current use of or plan to prescribe steroids) at weeks 4,16,32,48.
- Steroid-free biochemical remission (defined as CRP <ULN and calprotectin <200 and no current use of or plan to prescribe steroids) at weeks 4,16,32,48.
- Steroid-free endoscopic remission (defined as absence of ulceration i.e. ulcer subscore=0 and no current use of or plan to prescribe steroids) at week 48.
- Endoscopic remission at week 48 using video from end of trial. Defined by ulcer subscore=0 using central-reads from videos only.
- Endoscopic remission at week 48 using video and images from end of trial. Defined by ulcer subscore=0 using central-reads from videos and images.
- Endoscopic remission at week 48 using video and images from end of trial. Defined by ulcer subscore=0 using central-reads from videos and images where available, in combination with local-reads (whenever video or imaging central reads not available).
- Endoscopic remission at week 48 incorporating total SES-CD score. Defined by ulcer subscore=0 + SES-CD score <4, using central-reads from videos where available, in combination with local-reads (whenever video central reads not available).
- Endoscopic remission at week 48 defined by ulcer subscore=0 + SES-CD score <4, using only locally-read endoscopic scores.
- Endoscopic response (defined by SES-CD drop of ≥50% from baseline SES-CD score) at week 48 using only locally-read scores from all participants.
- Deep endoscopic remission (defined by total SES-CD score of 0) at week 48, using centrally-read videos where available, in combination with local-reads when video central reads not available.
- Deep endoscopic remission (defined by total SES-CD score of 0) at week 48, using only locally-read endoscopic scores.
- Endoscopic remission at week 48 in only those who had ulcers at the index colonoscopy (i.e. ulcer subscore of ≥1 on the index colonoscopy). Endoscopic remission defined as absence of ulceration i.e. ulcer subscore= 0). Centrally-read endoscopic scores will be used where available, and locally-read scores will be used only if central scores are not available.
- IBD-specific quality of life remission (defined by IBD-Q score of ≥170) at weeks 16,32 and 48.

- IBD-specific quality of life improvement/response (defined as IBD-Q increase of $\geq$16 from screening visit IBD-Q score) at weeks 16,32 and 48.
- Generic quality of life response (comparison of mean EQ-5D scores in each group) at each of weeks 16,32,48. The mapping used to calculate a utility value is taken from reference [Devlin et al. 2016.]
- Generic quality of life improvement/response (defined as EQ-5D increase of $\geq$10% from the screening visit EQ-5D score) at weeks 16,32,48.
- IBD-Q bowel symptom improvement/response ($\geq$8 increase in IBDQ bowel symptom domain from the screening visit) at weeks 16,32,48. Bowel symptoms are questions 1,5,9,13,17,20,22,24,26,29
- IBD-Q fatigue (question 2) improvement/response ($\geq$1 increase in IBDQ fatigue symptom domain from the screening visit) at weeks 16,32,48.
- Weight.
- Blood Cell Counts (Haemoglobin, White cell count, Neutrophil count) at weeks 4,16,32,48.
- Biochemical levels (albumin) at weeks 4,16,32,48.
- Metabolite levels (6TGN & 6MMP) at weeks 16,32,48.
- Perianal disease (4 non-exclusive classifications of: anal tag, anal fissure, anal fistula, perianal abscess) at weeks 4,16,32,48.
- Development of peri-anal abscess or fistula (development of peri-anal abscess / fistula vs no development of peri-anal abscess / fistula) at weeks 4,16,32,48.
- Development of endoscopic stricture by week 48 (development of stricture vs no development of stricture).
- Anti-TNF therapy at last observation: no anti-TNF therapy; monotherapy anti-TNF; combination therapy anti-TNF. Determined from adjudication of CRF data.
- Thiopurine at last observation within each participant: no thiopurine; optimised metabolite levels (6-TGN $\geq$235); non-optimised levels (6-TGN <235).

Exploratory Endpoints

Exploratory analyses (of data obtained alongside and to be linked to trial database) may be produced if the data are available in a timely fashion.

- *HLA-DQA1*05* variants at baseline.
- *NUDT15* variants at baseline.
- Infliximab drug levels at week 48 or at the timepoint closest prior to stopping infliximab.
- Imaging bowel inflammation (by comparing mean simplified MaRIA scores in each group) at week 48.

- Imaging remission (defined as <6 using simplified MaRIA score) at week 48.*
- Imaging response (defined as drop of $\geq$50% from baseline simplified MaRIA score) at week 48.*
- Imaging bowel damage (by comparing mean Lemann Index scores in each group) at week 48.*

*MRI simplified MaRIA and Lemann Index scores will be obtained only from centrally-read MRI scans.

Other samples collected during PROFILE

The following are captured as samples, but will not be processed during the primary trial follow-up period nor included in the trial database. Hence they will not be considered within the scope of this SAP but may be analysed later.

- RNA transcriptomic data.
- Serum proteomic data.
- Faecal microbiome data.
- Faecal metabolomic data.
- Histopathological data.

Trial Methods/

General Trial Design and Plan

(ICH E3;9)

The trial is a parallel randomised control trial with two arms. The treatments are two different treatment strategies (Accelerated Step-up; Top-down) of corticosteroids, immunomodulators and Infliximab, with additional protocolised criteria and regimens for rescue therapy; see the protocol for precise descriptions.

The randomisation is open-label, however the biomarker stratification factor measured at baseline is kept blinded to participants and clinicians.

Participants are screened and if recruited are then put on an initial standard course of treatment. Randomisation occurs approximately 2 weeks later, if eligibility is confirmed, to one of two dosing regimens. Observations are subsequently taken at weeks 4, 16, 32, and 48. Ad hoc visits may occur if the condition deteriorates and modifications to the dosing, within the guidelines given in the protocol, need to be made.

Inclusion-Exclusion Criteria and General Trial Population

(ICH E3;9.3. ICH E9;2.2.1)

The trial population is adult participants diagnosed with Crohn's disease within 6 months. See the protocol for a precise list of detailed criteria.

Randomisation and Blinding

(ICH E3; 9.4.3, 9.4.6. ICH E9; 2.3.1, 2.3.2)

Following biomarker assessment, participants in each biomarker subgroup were randomly assigned (1:1) to either "Top-Down" or "Accelerated Step-Up" therapy, using a computer-generated algorithm. At the outset of the trial this occurred within 14 days (plus/minus 5 days). However, given some delays for sites to get results of screening investigations returned in time for a baseline visit, an amendment was made so that this would occur 14 days after screening (plus 10 days). Stratified block randomisation was used, stratifying on biomarker subgroup, (IBDhi/IBDlo), mucosal inflammation (mild / moderate / severe) and disease location (colon-only/other) with a randomly generated block size.

Trial Variables

The following table is taken from the protocol

**Procedure**	**Screening Wk -2**	**Baseline Wk 0**	**Wk 4**	**Wk 16**	**Wk 32**	**Wk 48**	**Ad hoc**
Written consent	✓						
Demographics (DOB, gender, initials, ethnicity)	✓						
Medical History	✓	✓					
Disease assessment - Harvey Bradshaw Index (HBI)	✓	✓	✓	✓	✓	✓	✓
Perianal disease review	✓	✓	✓	✓	✓	✓	✓
Concomitant medication	✓	✓	✓	✓	✓	✓	✓
AE reporting		✓	✓	✓	✓	✓	✓
Weight in Kg	✓	✓	✓	✓	✓	✓	✓
Height in cm	✓						
Physical examination	✓	✓	✓	✓	✓	✓	✓
Eligibility confirmed		✓					
Randomisation		✓					
PAXgene RNA tube for biomarker assessment	✓						
PAXgene RNA tube for research sample	✓	✓		✓	✓	✓	✓
Serum tube	✓			✓	✓	✓	

**Procedure**	**Screening Wk -2**	**Baseline Wk 0**	**Wk 4**	**Wk 16**	**Wk 32**	**Wk 48**	**Ad hoc**
EDTA (Ethylenediaminetetraacetic acid) tube	✓						
Bloods (Full Blood Count, CRP, Urea & Electrolytes, Creatinine, Liver Function TEst)	✓†	✓	✓	✓	✓	✓	✓
Blood results review	✓	✓	✓	✓	✓	✓	✓
Screening (Hepatitis B & C, Varicella Zoster Virus, Thiopurine Methyltransferase)	✓						
Assessment of TB	✓						
Pregnancy test	✓						
Bloods (thiopurine metabolites)			✓$^{\alpha}$	✓$^{\alpha}$	✓$^{\alpha}$	✓$^{\alpha}$	✓$^{\alpha}$
Faecal Calprotectin	✓†	†		✓	✓	✓	✓$^{\beta}$
Buffered stool sample	✓					✓	
Stool sample for Microscopy, culture and sensitivity							✓
Primary outcomes review (steroids and surgery)			✓	✓	✓	✓	✓
IBDQ & EQ-5D Qol	✓	✓		✓	✓	✓	
Resource usage questionnaire		✓		✓	✓	✓	
Endoscopy	✓*					✓	
MR enterography (MRE)	✓¥					✓	

Baseline visit should be 2 weeks +10 days from screening. Week 4 visit should be within -10 days to +4 weeks of the schedule. Week 16 to 48 visits should be within ± 4 weeks of the schedule.

For much of the time during the COVID pandemic from March 2020 onwards, many clinic visits were carried out virtually. For most of the observations this had little impact, the exceptions being the colonoscopy, which could not take place, and two components of the HBI score, that would normally require a trained clinician to perform an assessment but was instead done by the participant themselves under virtual guidance. The final week 48 visit window for colonoscopy and MRE was extended up to three months following the end of trial visit. This timeline was selected based on expert clinical opinion, agreed with by the TSC, that results within this window would still be valid and accurate.

Variables collected outside of the time windows will not be used in efficacy analyses, but safety events will be included.

HBI values are a composite of several individual questions, with ordered categorical responses that are mapped to integers (0,1,2,..) and then added together. One of the components is a physical examination, which is normally undertaken by a trained clinician to assess for any abdominal masses. However, during the pandemic from March 2020, this in-person physical examination was not possible in many instances and a proxy assessment was made over the telephone by the participant guided by the local investigators. Collection of this data as reported by participants, was considered appropriate given the growing literature demonstrating reliability of participant-reported information and notable in this instance high association between HBI abdominal mass sub-score reported by participants compared to physician-assessed HBI abdominal sub-score (Echarri et al. Telemed J E Health, 2020).

A scaled version of the HBI will be considered that removes this abdominal mass component, as a complimentary endpoint for summary statistics and figures. The full HBI score, using either the clinician's or participant's assessment, will be used in the primary analysis, but sensitivity analyses will treat these as missing, and potentially impute the missing component. The primary endpoint is an aggregate over all visits, that assesses if HBI <5 and/or absence of objective evidence of inflammation, and if surgery occurred or an additional or extended induction course of steroids have been taken; so the value of this binary endpoint can still be determined, even if the HBI at one or more visits has the component missing.

Sample Size

(ICH E3; 9.7.2. ICH E9; 3.5)

Remission rates were estimated using data pertaining to the clinical phenotype of IBDhi and IBDlo participants and data from the literature regarding response to early anti-TNFα (including the original “Step Up-Top Down” trial, D’Haens et al. Lancet, 2008; the SONIC trial, Colombel et al. NEJM, 2010; and subgroup analyses of large anti-TNFα trials, D’Haens. Nat Rev Gastro Hepatol 2010).

Estimated remission rates were: IBDhi: “Accelerated Step-Up” 0.3, “Top-Down” 0.7; IBDlo: “Accelerated Step-Up” 0.8, “Top-Down” 0.9. The prevalence of IBDhi/ IBDlo is 0.5/0.5 based on all of the cohorts in whom the classifier has been assessed. The primary comparison is powered as an interaction analysis, where the interaction refers to the difference between the relative benefits of "Top-Down" over "Accelerated Step-Up" in each subgroup. Based on the remission rates and subgroup prevalence rates above, an interaction of 0.3 can be detected with a power of 92% at a 2-sided 5% significance level with a total sample size of 333.

To allow for a ~17% drop out rate, from the outset of the trial the goal was to recruit 400 participants across approximately 50 sites around the United Kingdom.

General Considerations

Timing of Analyses

The trial closed to new participant screening in December 2021, with the final randomisations for the trial in January 2022. The recruitment rate had initially slowed substantially during the COVID pandemic but subsequently increased, so that the trial almost recruited to target (n=390 participants randomised). In addition, the dropout rate was less than planned for, as monitored during ongoing DMC and TSC meetings, hence the final recruitment number of n=390 was felt sufficient to maintain adequate power according to the original trial design.

Analysis Populations

(ICH E3; 9.7.1, 11.4.2.5. ICH E9; 5.2)

- **Full population**
    - All participants who were randomised and met eligibility criteria (all inclusion and no exclusion criteria) for the PROFILE trial.
- **Safety population**
    - All participants who were randomised, met eligibility criteria (all inclusion and no exclusion criteria), and received any trial treatment (including the initial induction course of steroid medication).
- **Modified per-protocol treatment population** - All participants who did not substantially deviate from the protocol for treatment to be determined on a per-participant basis. All participant cases will be reviewed by an expert adjudication committee (Prof Miles Parkes, Dr James Lee, Prof James Lindsay).
    - Treatment escalation without meeting criteria for flare.
        - Treatment escalation in absence of HBI score ≥7, AND CRP >ULN OR calprotectin ≥200ug/ml.
    - Treatment not escalated despite meeting criteria for flare.
        - HBI score ≥7, AND raised CRP >ULN OR calprotectin ≥200ug/ml.
    - Treatment escalated but not in accordance with the trial protocol.
        - E.g. immunomodulator step skipped and started on infliximab; infliximab skipped and started on adalimumab; infliximab or immunomodulator not prescribed but given additional course of steroid medication. This list is not exhaustive.
- **Modified per-protocol treatment, schedule and procedures population** - All participants who did not substantially deviate from the protocol including all aspects of treatment, trial visits and performance of trial procedures, to be determined on a per-participant basis. All participant cases will be reviewed by an expert adjudication committee (Prof Miles Parkes, Dr James Lee, Prof James Lindsay).
    - Observations made outside the visit window, using the amended time window from Baseline for all participants.

- **Pre-COVID population**
  - All participants completing the study prior to March 2020.
- **Peri-COVID population**
  - All participants randomised or in follow-up after March 2020.

Covariates and Subgroups

(ICH E3; 9.7.1, 11.4.2.1. ICH E9; 5.7)

The analyses will estimate for the main effect of the biomarker and the interaction between the biomarker and treatment, adjusting for baseline variables:

- Mucosal Inflammation (mild / moderate / severe)
- Disease location (ileal / colonic / ileocolonic) as recorded locally at baseline on the Medical history form
- Disease behaviour (inflammatory / other) as recorded locally at baseline on the Medical history form
- Smoking status (never / former / current)
- Age (16-39,40-64,65+)
- BMI (0-19.9, 20-24.9,25-29.9, 30+)
- CRP (continuous)
- Calprotectin (continuous)
- Course of glucocorticoids prior to trial enrolment (yes/no)
- Time from date of endoscopy to date of screening
- HBI score

Subgroup analyses of potential interest. Subgroups with potential biological plausibility for differential effects on biomarker-treatment interaction effect were selected for assessment. The three-way interactions, and two-way interactions between treatment and subgroup, will be presented graphically using a forest plot.

- Age in years at baseline (<60 vs ≥60)
- Gender (male vs female)
- Ethnicity (European origin vs other)
- Smoking status at baseline (current smoker vs other)
- Obesity (BMI <30 vs ≥30)
- Time from diagnosis to inclusion in days (<30 vs ≥30)

- Disease location (ileal / colonic / ileocolonic) as recorded locally at baseline on the medical history form
- Stricturing disease at baseline (passable vs non-passable stricture based on SES-CD sub-score)
- Severe endoscopic severity at baseline using SES-CD score (≥6 vs <6 for ileocolonic and colonic disease, and ≥4 vs <4 for ileal disease)
- Presence of ulcers at baseline using SES-CD ulcer sub-score (0 vs ≥1 for ulcer sub-score in total SES-CD)
- Steroid use at baseline (prednisolone vs budesonide)
- Previous abdominal surgery at baseline (unrelated to Crohn's disease) vs no previous abdominal surgery
- Any other immune-mediated inflammatory disease at baseline (IMID) vs no other IMID

The following post hoc subgroups were added after SAP v1 and seeing the initial analysis results that followed.

- CRP (< upper quartile vs > upper quartile)
- Calprotectin (< upper quartile vs > upper quartile)
- SES-CD total score (< upper quartile vs > upper quartile)
- SES-CD "Presence and size of Ulceration" subscore maximum over the 5 different anatomical regions (>=3 vs <3)

Post-Baseline Subgroups

The following groupings are only observable post-baseline. As such they cannot be used to determine treatment choice, but may be indicative of a mediating effect of the treatment mechanism. They are included as tertiary endpoints in 0.

The primary and secondary endpoints will have summary tables provided using these as subgroups, but not formal regression analysis. Any comparison between such post-baseline subgroups is dependent on there not being any confounding between the subgroup incidence and the endpoint, which is untestable and thus such comparisons will be considered with caution.

- Development of peri-anal fistula by week 48 (development of peri-anal fistula vs no development of peri-anal fistula).
- Development of endoscopic stricture by week 48 (development of stricture vs no development of stricture).
- Three levels of therapy: no anti-TNF therapy; monotherapy; combination therapy.
- Thiopurine at last observation within each participant: no thiopurine; optimised metabolite levels (6-TGN ≥235); non-optimised levels (6-TGN <235).

Potential Data

The following data may be available at the time of final analysis, and if so, will be used to define subgroups.

- Optimised infliximab trough drug levels (≥5ug/g) vs non-optimised infliximab trough drug levels (≤5ug/g). This will be treated as a post-baseline subgroup.
- HLA-DQA1*05 variants vs no HLA-DQA1*05 variants.

Coding Details

The majority of variables to be used as predictor variables in the regression models are binary. The parameterisation used to represent the effect of a binary variable will be of the form +β/2, and -β/2 for each of the two possible values. Thus β directly estimates the difference in the expected values between the two values {+β/2 – (-β/2)}= β . This is achieved in the coding by setting the *contrast* to be (+1/2, -1/2). Furthermore when interactions with a second variable are included, this interpretation of the *main* effect is preserved, as the equal-weighted average of the predicted effect of the first variable within each of the levels of the second variable. To verify this, one can form the design matrix for each possible combination of predictor variables, from which the predicted value is obtained by calculating the matrix product with the vector of coefficients; inverting the design matrix gives the interpretation of the coefficients—differences of weighted averages of the group-level expected values—which is given below for the treatment-biomarker interaction model

Treatment	Top-up		Step-down	
Biomarker	Hi	Lo	Hi	Lo
Intercept	1/4	1/4	1/4	1/4
Treatment Main Effect	1/2	1/2	-1/2	-1/2
Biomarker Main Effect	1/2	-1/2	1/2	-1/2
Interaction	1	-1	-1	1
**Stratified Treatment Effects**				
Treatment in Hi Group =<br>Main Treatment+1/2.Interation	1	0	-1	0
Treatment in Lo Group=<br>Main Treatment -1/2 .Interaction	0	1	0	-1

If 3 such variables are combined, as per a subgroup analysis, then the equivalent set of contrasts is below, using smoking as an example subgroup. The marginal distribution of the subgroups will be used to weight the subgroups when calculating main effects, and 2nd-order interactions. So if the marginal distribution has weights p & q, the contrasts will be (q , -p) . For the smoking illustration below we take p, q as ¼, ¾ respectively.

Treatment	Top-up				Step-down			
Biomarker	Hi		Lo		Hi		Lo	
Smoking	Current	Other	Current	Other	Current	Other	Current	Other
Intercept	0.0625	0.1875	0.0625	0.1875	0.0625	0.1875	0.0625	0.1875
Main Effects								
Treatment	1/8	3/8	1/8	3/8	-1/8	-3/8	-1/8	-3/8
Biomarker	1/8	3/8	-1/8	-3/8	1/8	3/8	-1/8	-3/8
Smoking	1/4	-1/4	1/4	-1/4	1/4	-1/4	1/4	-1/4
2-way interactions								
Treatment-Biomarker	1/4	3/4	-1/4	-3/4	-1/4	-3/4	1/4	3/4
Treatment-Smoking	1/2	-1/2	1/2	-1/2	-1/2	1/2	-1/2	1/2
Smoking-Biomarker	1/2	-1/2	-1/2	1/2	1/2	-1/2	-1/2	1/2
3-way Interaction	1	-1	-1	1	-1	1	1	-1

Missing Data

(ICH E3; 9.7.1, 11.4.2.2. ICH E9;5.3. EMA Guideline on Missing Data in Confirmatory Clinical Trials)

Generally, total population size will be reported in summary tables (and will highlight any missing values). Summary statistics will use complete case analysis, which assumes Missing Completely At Random. Regression analysis that adjust for covariates, or account for within-participant correlation in repeat-measures analysis, will be performed which assumes Missing At Random.

See section 0 for a specific discussion and sensitivity analyses to consider the partially missing component of the HBI score.

If the proportion of missing values falls below 5% for an endpoint then no further sensitivity analyses will be performed as the scope to influence the conclusion is too small.

To deal with any missing baseline covariates Multivariate Imputations using Chain Equations (Buuren & Groothuis-Oudshoorn 2011) is used to provide 5 imputed complete data sets. This is repeated separately for each endpoint/analysis as the endpoint is used as predictor of the missing baseline values; for any longitudinal analysis of repeated observations, the per-patient average of the repeated observations is used, along with an average of the visit numbers with observations. Rubin's rules are used to combine the analysis from the multiple imputed data sets.

Where bootstrapping is used to provide confidence intervals around standardised effects on the additive scale, then bootstrapping is performed as an outer loop around repeated multiple imputations (Schomaker & Heumann 2018)

Multiple Testing

(ICH E3; 9.7.1, 11.4.2.5. ICH E9; 2.2.5)

To formally control for multiple testing, we will perform a closed testing procedure over the primary and five secondary endpoints (see section 0 and 0) testing the biomarker-treatment interaction and restricting the family-wise type 1 error rate to an overall 5% significance level.

The methodology to combine together gate-keeping and Holm-Bonferroni methods in formal hypothesis testing will be used (Bretz et al. Stat Med 2009), with the diagram below defining how the significance levels will be transitioned assuming an initial configuration of 5% at the primary endpoint (relapse-free remission) and 0% on all other tests.

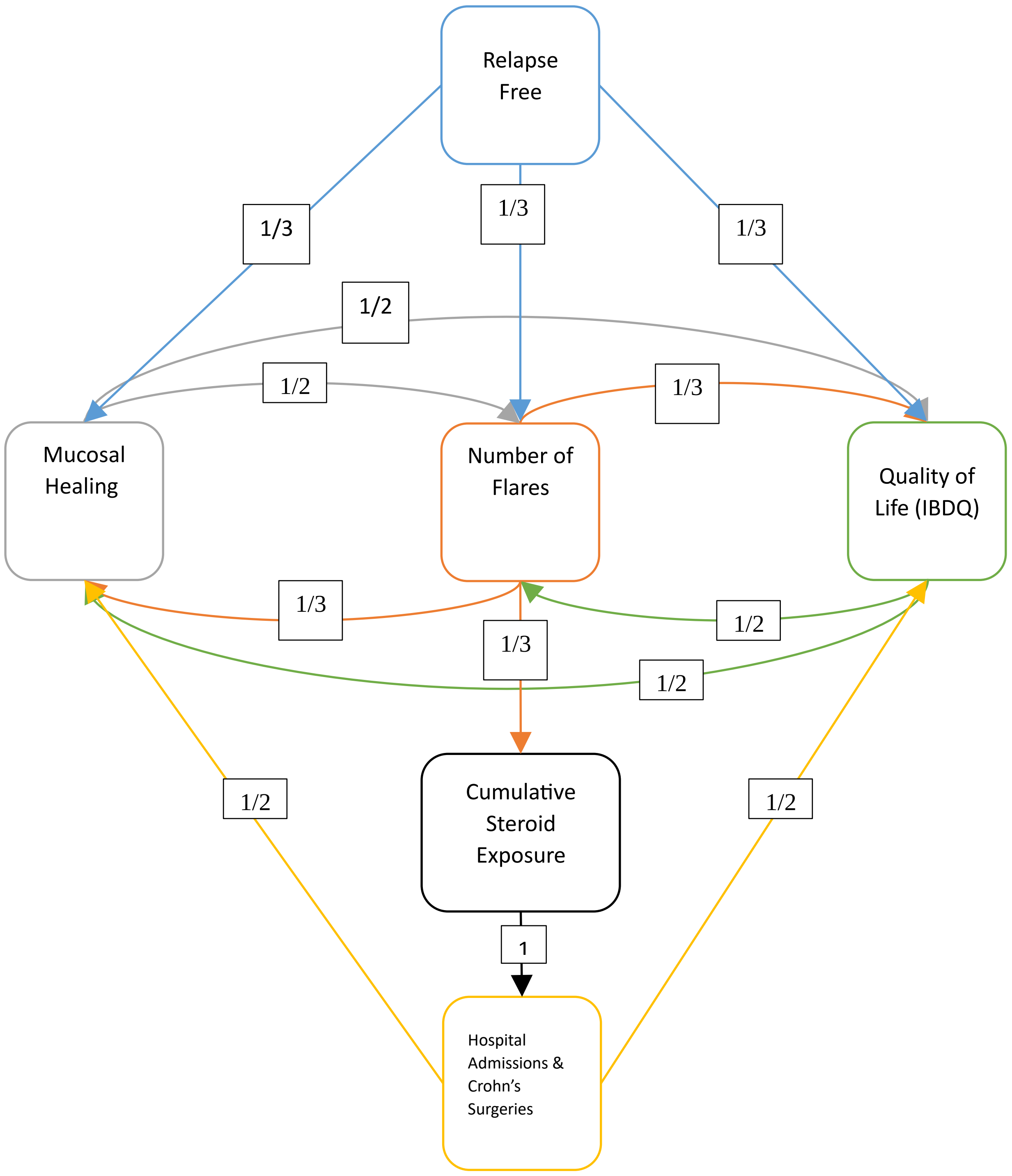

The diagram shows the flow of significance level spending in the sequential multiple testing procedure.

Summary of Trial Data

All continuous variables will be summarised using the following descriptive statistics: n (non-missing sample size), mean, standard deviation, median, maximum and minimum. The frequency and percentages (based on the non-missing sample size) of observed levels will be reported for all categorical measures. In general, all data will be listed, sorted by treatment, biomarker and participant, and when appropriate by visit number within participant. All summary tables will be structured with a column for each treatment-biomarker combination, and marginally for each treatment. The tables will be annotated with the total population size relevant to that table/treatment, including any missing observations.

Participant Disposition

The Visit Completion form and Withdrawal form will be used to determine how far each participant reached within the trial, and provide reasons for withdrawal from data collection, and from treatment.

A figure of cumulative recruitment over time will be provided.

A skeleton CONSORT diagram should be provided in this section that provides an explicit statement of what statistics are to be provided. http://www.consort-statement.org/.

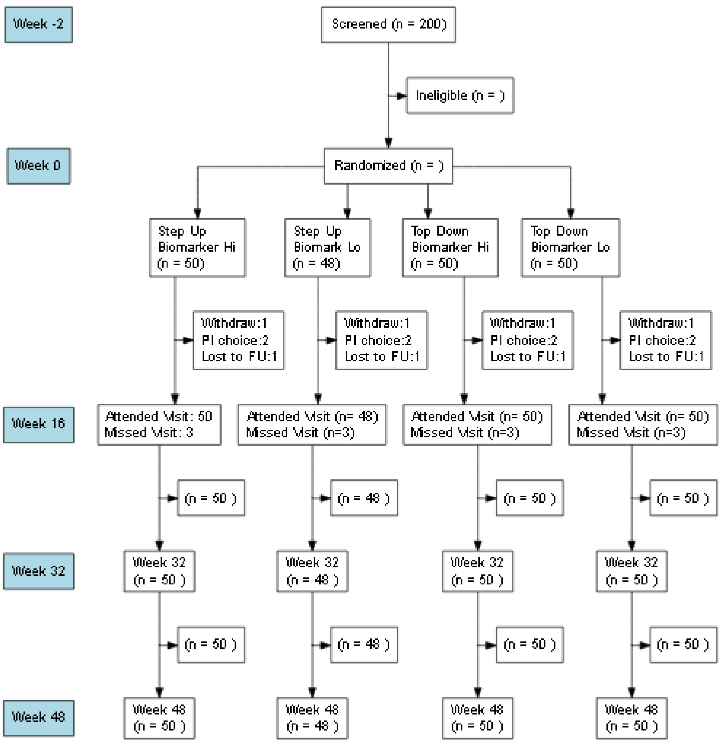

Derived variables

- Primary endpoint, see section 0 and 0 for detailed description of how the endpoint is derived.
- Several endpoints are the cumulative incidence or total over all visits
  - Number of Flares

- Cumulative Steroid Use
- Hospitalisation
- Surgery

Protocol Deviations

Modifications to participants' dosing or medication had a set of guidelines described in the protocol, termed as "escalation". Deviations did occur in both directions of escalating or not escalating, in breach of the protocol guidelines. But this is a very broad description, and the large variety of medications and doses used make it impractical to give a definition based on trial data to identify non-compliance. A 3-member committee will be assembled (Prof Miles Parkes, Dr James Lee, Prof James Lindsay) to take expert judgement based on the totality of a participant's notes as to whether, and at which visit, they were non-compliant, and thus would leave the visit-specific Per Protocol treatment population and a more stringent Per Protocol treatment, schedules and procedures population.

The COVID pandemic meant that face to face clinic visits had to be paused across many sites, and so the data collection process and visit windows were modified. See section 0 for a full description.

Demographic and Baseline Variables

Each participant undergoes a screening visit at which data is observed, and if eligible and consenting they commence an initial treatment, common to both arms. Hence the data from the screening visit is representative of the participants underlying state of health and will be used to present "Baseline" data.

The following variables will be summarised as per section 0

- Gender
- Age, Age group (16-39,40-64, 65+)
- Ethnicity
- Smoking Status
- BMI, BMI group (0-19, 20-24,25-29, 30+)
- HBI score
- Modified HBI score (removing the abdominal examination component)
- IBDQ score
- CRP
- Albumin
- Haemoglobin
- Faecal Calprotectin
- SES-CD score
- Disease Location

- Disease Behaviour
- Time from diagnosis to consent
- Biomarker

Concurrent Illnesses and Medical Conditions

A fixed list of specific medical conditions is assessed systematically at baseline visit, which will be tabulated as to the count and proportion of participants with each condition as per section 0.

Prior and Concurrent Medications

A fixed list of prior steroid medications is assessed systematically at screening, which will be tabulated as to the count and proportion of participants for each medication as per section 0.

Concurrent medications are captured during the trial and will be surmised with line listings.

Treatment Compliance

Treatment compliance is assessed as a binary variable at each visit, and participants being included or excluded from the per-protocol population is where this information is captured. See section 0

Any individual participants and specific visits, where not clear if treatment compliance has occurred, will be discussed and reviewed by the adjudication committee (Prof Miles Parkes, Dr James Lee, Prof James Lindsay). Majority consensus from this committee will be used to determine whether treatment compliance has occurred in accordance with the protocol or not.

Efficacy Analyses

The main focus will be on estimating the interaction between treatment and biomarker for each of the endpoints, defined as a difference in differences. To compliment this, the treatment effect within each biomarker subgroup will also be estimated, along with a marginal effect averaging equally across the subgroups. The marginal effect corresponds to the treatment effect when knowledge of the biomarker is not available for individual participants, for a population where the distribution of the biomarker is equally split 1:1 between Hi and Lo.

Each analysis will have 4 comparisons:

- Treatment effect for Hi.
- Treatment effect for Lo.
- Biomarker-Treatment interaction: the difference between the previous two comparisons.
- Main treatment effect: average of the Hi- and Lo- treatment effects.

For endpoints that are analysed on an absolute difference scale these contrasts are simple linear transformations of the model parameters. For endpoints/models that use odds ratios, relative risks or any non-linear function of the parameter values, the model parameters will be used to obtain predicted values on the scale of the endpoint. To calculate the main treatment effect, averages across biomarkers will be calculated, and the contrasts then potentially transformed back onto the non-linear scale of choice, using the standardisation, a.k.a. parametric g-formula, method (Hernán & Robins, Chapter 13). If adjustment for covariates leads to non-convergence on the absolute difference scale for binary variables, then an alternative will be to use a logistic scale, and the same standardisation approach taken to obtain predicted values on the endpoint scale, averages taken for combinations of treatment, biomarker and subgroup, and contrasts taken on the absolute difference scale as desired. Inference to provide SE, 95% CIs, and p-values will be done analytically for the linear scale, or using non-parametric bootstrapping for the non-linear scale.

The primary endpoint, and mucosal healing (using SES-CD score) are both binary variables, and will be analysed using a generalised linear model with an additive link function to estimate absolute differences in incidence. Number of Flares, number of Steroid Courses, and count of Hospitalisation or Surgery will be analysed using a linear model. Quality of life is observed over repeated visits and will be analysed using a mixed effect repeat measure analysis with a clustered participant-level residual error with unstructured covariance over visits, fixed effects for visit, and all other covariates assumed to have a constant fixed effect over time.

The randomisation stratum will be adjusted for as baseline covariates: biomarker subgroup, (IBD^{hi}/IBD^{lo}), mucosal inflammation (mild / moderate / severe) and disease location (colon-only/other) plus the main effect of the covariates listed in section 0. Treatment and the interaction between biomarker and treatment will also be included in the models. Quality of life and Mucosal Healing (endoscopic remission) will also adjust for the values observed at screening.

The Full Analysis population will be used for all presentations of efficacy variables, complimented by using the Per Protocol population in some select cases. The p-values from the full analysis population will be used in the process described in section 0 to control for multiple testing, using just the treatment-biomarker parameter.

The Safety Population will be used to summarise the safety endpoints.

Pandemic sensitivity analyses will compare pre-1 March 2020 with 1 March 2020 and beyond. This month was selected as this was the date of the first COVID-19 wave building in the UK, resulting in a national period of lockdown. Thereafter, for the duration of recruitment and follow-up, subsequent waves of incident COVID-19 followed in the UK.

In formal statistical terms the null hypothesis is that the interaction between treatment and biomarker is zero. This is asking if the treatment effect in each of the biomarker subgroups is the same. As such it does depend directly on the choice of scale used to measure the (non-zero) treatment effect, and the modelling assumptions. Statistical significance will be assessed in a two-sided manner using a 5% level. However, the focus is on estimation of the magnitude of the interaction and providing a measure of uncertainty around the estimate using 95% confidence intervals. All assumptions for regression models will be assessed by viewing plots of the residual values

Primary Efficacy Analysis

The primary endpoint is defined as:

Incidence of sustained surgery and steroid free remission from completion of a protocolised (maximum 8-week regimen) steroid induction treatment through to week 48.

*remission = HBI≤4 and/or absence of objective evidence of inflammation.

This is equivalent to HBI≤4 at all assessments, or if symptoms are present (HBI≥5) there is no objective evidence of inflammation (CRP<ULN and calprotectin<200). Requirement for an additional or extended induction course of systemic steroids or surgery for active Crohn's disease would result in failure to meet primary outcome measure.

So if the HBI is 5 or greater at any scheduled or unscheduled visit due to active Crohn's disease as assessed by local PI and corroborated by either a raised CRP>ULN or raised calprotectin >200 or both, or surgery occurs, or steroids are prescribed (above the protocolised maximum 8 week induction treatment), then the primary endpoint will not have been met.

The primary analysis will proceed as per section 0 using a generalised linear model, and additionally adjusting for the HBI total score observed at screening, with an additive link function, or logistic link if convergence problems arise.

A sensitivity analysis will be performed using the intersection across all visits of the visit-specific per-protocol populations.

An exploratory figure will present the proportion of participants meeting the primary endpoint at or before each time point using a Kaplan-Meier estimate with 95% confidence intervals.

Secondary Efficacy Analyses

Mucosal Healing

This is the SES-CD score observed at week 48 and will focus on endoscopic remission (defined as absence of ulceration including aphthous ulcerations (ie ulcer subscore=0).

The analysis will use a generalised linear model with an additive link function, as per section 0, and in addition to the standard adjustment covariates, will add the baseline SES-CD score.

The Week 48 Per Protocol population will be used for a sensitivity analysis.

Number of Flares

This endpoint is defined cumulatively across all visits, including ad hoc visits. The analysis will use linear regression as per section 0.

A sensitivity analysis will be performed using the intersection across all visits of the visit-specific per-protocol populations.

Quality of Life IBDQ (Inflammatory Bowel Disease Questionnaire)

There are 32 questions, each of which is recorded on a seven-point Likert scale, where 1 is the worst score and 7 the best (Guyatt et al. Gastroenterology 1989). The total score is derived by adding these numerical values up over the 32 questions for each participant-visit.

Any missing values to individual questions will be ignored and the score scaled to reflect the number of “non-missing” values.

The endpoint is observed over repeated visits and the values at the scheduled visits (but not ad hoc) will be analysed using a mixed effect repeat measure analysis with a clustered participant-level residual error with unstructured covariance over visits, fixed effects for visit, and all other covariates assumed to have a constant fixed effect over time. The standard set of covariates will be used for adjustment with the addition of the baseline value for IBDQ. A treatment effect estimate assumed to be constant over time will be used for the formal closed multiple testing procedure. But individual visit-specific treatment effects at all scheduled visits will also be presented.

Observations taken at participant-visits belonging to the visit-specific per-protocol population will be used for a sensitivity analysis.

Cumulative Steroid Exposure

This endpoint is defined as the total number of courses of steroids taken, including those that were increased in line with the protocol, and any non-compliant increases to treat active Crohn’s disease. The analysis will use linear regression as per section 0.

A sensitivity analysis will be performed using the intersection across all visits of the visit-specific per-protocol populations.

Number of Hospital Admissions and Surgeries for Crohn’s disease

The incidence of surgery for Crohn’s disease is captured directly within the CRF. An SAE that requires hospitalisation will capture hospital admissions. Hence the count is taken across all visits, including ad hoc visits, and aggregated into a single endpoint per participant.

The analysis will proceed as per section 0 using a generalised linear model with an additive link function.

A sensitivity analysis will be performed using the intersection across all visits of the visit-specific per-protocol populations.

HBI

HBI score will be presented using spaghetti plots (with and without ad hoc visits), and mean values with CIs at each visit and biomarker-treatment presented. A mixed effect repeat measure analysis will be performed with a clustered participant-level residual error with unstructured covariance over visits, fixed effects for visit, and all other covariates assumed to have a constant fixed effect over time. The standard set of covariates will be used for adjustment with the addition of the baseline value.

The analysis will performed twice on two variations of the endpoint:

- a version of the score that excludes the components based on a physical examination (Abdominal Mass, Complications) which had to be collected by patients' own examination during the covid lockdown.
- using the participant reported physical examination.

Incidence of Surgery

The individual component of the primary endpoint will be considered in isolation.

The analysis will proceed as per section 0 using a generalised linear model with an additive link function.

Incidence of Steroid Use

The individual component of the primary endpoint will be considered in isolation. This is not the cumulative count of steroids, but rather simply if steroids were used at any point in time above the maximum 8 week initial induction treatment.

The analysis will proceed as per section 0 using a generalised linear model with an additive link function.

COVID sensitivity analyses

For each of the primary and secondary endpoints, the analyses will be repeated for the pre- and peri-covid populations.

Seemingly Unrelated Regression

The components of the primary and secondary endpoints, in particular those that relied on colonoscopy visits, were not able to be performed during the lockdown restrictions, and thus have higher incidence of missing values. Hence a joint model across several key endpoints, for the week 48 value, may increase precision to the comparisons by accounting for the within-participant correlation and thus make better use of participants with partial observations for a subset of the endpoints. A joint linear model that allows an unstructured residual error covariance matrix will be fitted to the following endpoints on their original, untransformed, scales.

- HBI score
- Centrally-read SES-CD
- Locally-read SES-CD
- Number of Flares
- IBDQ

- Cumulative Steroid exposure
- Number of Hospital admissions and surgeries
- Faecal Calprotectin – log transformed

Fixed effects will be stratified by the endpoint, and adjust for treatment-biomarker interaction plus the baseline covariates detailed in 0. The baseline or screening value of the endpoint will be used as an adjustor where a meaningful baseline value exists, or set to a dummy value of 0. The number of non-missing baseline values for centrally-read SES-CD was too small (25) and caused confounding with non-full-rank design matrix, hence it was also set to a dummy value of zero for centrally-read SES-CD.

The numbers of participants in the 65+ age group were low, and so were merged into a combined 40+ category.

Values observed at visits before week 48 will not be considered.

The same set of comparisons as per the primary analysis will be presented with estimates, SE, confidence intervals and p-values.

The R code will use the gls function taken from the nlme package and will be of this nature, as an illustration:

```
gls(value~variable/(treatment*biomarker + covariate +
baseline_value),correlation = corSymm(form=~var_num|subjid),
weights=varIdent(form=~1|variable))
```

where var_num is the endpoint, variable, mapped to a sequence of consecutive integers, as demanded by the software.

Censored observations for Faecal Calprotectin, where the observation is above the limit of detection will be handled using the EM algorithm [Dempster, Laird & Rubin, 1977], and a simulation exercise will be performed to establish that the implementation with bespoke code provides an unbiased estimate with correct coverage of confidence intervals.

Subgroup Analyses

For each of the primary and secondary analysis, the subgroup analyses will be performed using the full analysis population.

Summary tables broken down by treatment-biomarker-subgroup will be produced

Sequentially, and considering in turn each subgroup from section 0, the main effects, 2nd- and 3rd-order interactions will be presented, with the estimates and 95% confidence intervals, but not the p-values. Interpretation of the estimates' magnitude is more important that consideration of statistical significance, including whether the subgroup could modify the optimal choice of treatment. Should a subgroup interaction prompt reporting in academic journals, then a stratified version of the model may be reported, as distinct from the difference-of-difference-of-difference that a 3-way interaction represents, and would be calculated outside the main statistical report.

Tertiary and Exploratory Efficacy Analyses

Summary statistics as described in section 0 will be provided for the tertiary and exploratory endpoints as detailed in sections 0 and 0, stratified by visit, treatment and biomarker. For continuous

variables, the change from baseline (or screening value where appropriate) will be plotted over time, with lines connecting the observations within an individual participant (a spaghetti plot, with and without ad hoc visits), with colour coding used to distinguish between the treatment-biomarkers. Box and Whisker plots will also be provided to represent their distribution in a cross-sectional manner. Kaplan-Meier plots will be presented for time-to-event endpoints.

Safety Analyses

Adverse Events

Safety Analyses will use the Safety Population and be broken down by biomarker-treatment, and treatment (pooling across biomarker).

The data have MedDRA coding provided for each event reported, along with an assessment of causality, seriousness, and expectedness. For each preferred term, there will be a count of the number of participants (any repetitions of an AE within the same participant will be ignored), proportion of the population, and number of events (to include any repetitions), broken down as described above, and also split into serious and non-serious AEs. The preferred terms will be grouped by the System Organ Class. Crohn's disease complications requiring surgical interventions will be coded according to the surgical operation performed.

For more meaningful reporting of SAEs, a grouping of the preferred terms will be defined (see appendix) and the same format of reporting provided.

A figure will be provided, a "Dot Plot" that, for each grouping of preferred terms, gives the absolute risk for each biomarker-treatment on a line representing the unit interval and also give the relative risk estimate and 95% CI on a logarithmic relative risk scale.

A line listing of each AE, split into serious and non-serious AEs, will be provided sorted by treatment-biomarker, SOC, PT, causality and expectedness, in that order of sorting.

Pregnancies

A line list of pregnancies that occurred for participants will be produced.

Reporting Conventions

P-values ≥0.001 will be reported to 3 decimal places; p-values less than 0.001 will be reported as "<0.001". The mean, standard deviation, and any other statistics other than quantiles, will be reported to one decimal place greater than the original data. Quantiles, such as median, or minimum and maximum will use the same number of decimal places as the original data. Estimated parameters, not on the same scale as raw observations (e.g. regression coefficients) will be reported to 3 significant figures. Percentages will be rounded to whole numbers unless they are less than 1%.

Technical Details

R version 4.1 will be the software tool used, correct at the time of writing. Full documentation of all versions and add-on packages will be recorded as the report is generated.

Any outputs will have

- The date and time included
- The name of the code file that produced the analysis

- The author
- A log capturing the version of the software and any external add-on code used.

Summary of Changes

From Protocol

At the TSC held on 10th September 2018, at which 30 participants had been randomised, the biomarker thresholds had been further optimised, and a decision was taken to accept the modified thresholds used to define IBDhi and IBDlo using this biomarker blood-test. A few of the existing participants' biomarker value were changed but given this was very early on in the trial, subsequent stratified randomisation was able to be maintained and remained blinded to the trial management group, the TSC, participants and site principal investigators.

From SAP V1

- Modification of adjusting covariates to include baseline HBI.
- Added details on using multiple imputation in conjunction with bootstrapping, to deal with missing values in baseline covariates.
- Additional endpoints relating to endoscopy (central & local reading) and only using incidence of ulcers, rather than composite with the total SES-CD.
- Additional Kaplan-Meier curve to describe time to primary endpoint.
- Seemingly Unrelated regression uses faecal calprotectin on a log-transformed scale and uses EM algorithm to deal with censored observations above limit of detection.
- Clarification of primary outcome measure to include objective markers of active inflammation (CRP and calprotectin) in the definition of presence and absence of remission (reflecting their inclusion in definition of flare in the protocol). This was agreed by the TSC 26/04/2023:
- Incidence of sustained surgery and steroid free remission from completion of a protocolised (maximum 8-week regimen) steroid induction treatment through to week 48.
- Clarification of secondary endpoint re method to define endoscopic remission
    - Endoscopic remission at week 48 (defined by absence of ulceration i.e. SES-CD ulcer subscore = 0). Centrally-read endoscopic scores will be used where available. Where these are not available locally-read scores will be used
- Modification of secondary endpoint relating to timepoints for assessment of quality of life
    - Quality of life averaged across weeks 16, 32 and 48 (using disease specific IBD-Q score).
- Addition of the following tertiary end-points for analysis
    - Incidence of sustained surgery and steroid free remission from completion of a standard (maximum 8-week regimen) steroid induction treatment through to week 48 (when remission defined using clinical parameters alone, HBI < 5).
    - Clinical remission (defined as HBI < 5) at weeks 4,16, 32 and 48.

   - Average clinical disease activity (comparison of mean HBI scores in each group) at weeks 4,16,32 and 48.
   - Time to event, time from baseline to first flare or need for surgery for Crohn's disease, which may occur during the protocolised induction course of steroid medication.
   - Time to event, time from baseline to second flare or need for surgery for Crohn's disease.
   - Time to event, time from baseline to starting on anti-TNF therapy for Crohn's disease.
   - Patient reported clinical remission (using score generated from abdominal pain and stool frequency components of HBI score – abdominal pain $\leq$1 and stool frequency $\leq$3) at weeks 4,16,32,48.
   - Development of peri-anal abscess or fistula (development of peri-anal abscess / fistula vs no development of peri-anal abscess / fistula) at weeks 4,16,32,48.
   - Anti-TNF therapy at last observation: no anti-TNF therapy; monotherapy anti-TNF; combination therapy anti-TNF.
   - Thiopurine at last observation within each participant: no thiopurine; optimised metabolite levels (6-TGN $\geq$235); non-optimised levels (6-TGN <235).
- Modifications to the details of covariate adjustment in the Seemingly Unrelated Regression model.

From SAP V2

- Additional endoscopy tertiary endpoints
- Extra subgroups
- Extra clause to meet eligibility criteria for the Safety population definition.
- Clarifications and details regarding safety reporting.

Quality Control

The derivation of the primary endpoint is complex as it involves multiple visits and multiple questions from the CRF. Hence it will be derived by a separate QC statistician and compared, and also a random set of 10 participants will be checked by hand.

The primary analysis will be replicated by a separate QC statistician. Overall, the code and data manipulations will be reviewed by a separate statistician.

References

Bretz F, et al. (2009). A graphical approach to sequentially rejective multiple test procedures. *Stat Med* 28:586-604.

Colombel JF, et al. (2010). Infliximab, azathioprine or combination therapy for Crohn's disease. *NEJM* 362:1383-1395.

D'Haens G, et al. (2008). Early combined immunosuppression or conventional management in participants with newly diagnosed Crohn's disease: an open randomised trial. *Lancet* 371:660-667.

D'Haens G, et al. (2010). Top-down therapy for IBD: rationale and requisite evidence. *Nat Rev Gastroent Hepatol* 7:86-92.

Devlin, N., Shah, K., Feng, Y., Mulhern, B. and van Hout, B. (2016). Valuing Health-Related Quality of Life: An EQ-5D-5L Value Set for England. *Health Economics* 27, 7-22.

Echarri A, et al. (2020). The Harvey-Bradshaw Index adapted to a mobile application compared with in-clinic assessment: The MediCrohn Study. *Telemed J E Health* 26, 80-88.

Guyatt G, et al. (1989). A new measure of health status for clinical trials in inflammatory bowel disease. *Gastroenterology* 96, 804-810.

Hernán MA, Robins JM (2020). Causal Inference: What If. Boca Raton: Chapman & Hall/CRC.

White IR and Thompson SG (2005). Adjusting for partially missing baseline measurements in randomised trials. *Stat Med* 24**:**993-1007.

Dempster, A. P., N. M. Laird, and D. B. Rubin. "Maximum Likelihood from Incomplete Data via the EM Algorithm." Journal of the Royal Statistical Society. Series B (Methodological) 39, no. 1 (1977): 1–38. http://www.jstor.org/stable/2984875.

Schomaker, M, Heumann, C. Bootstrap inference when using multiple imputation. *Statistics in Medicine*. 2018; 37: 2252– 2266. https://doi.org/10.1002/sim.7654

Stef van Buuren, Karin Groothuis-Oudshoorn (2011). mice: Multivariate Imputation by Chained Equations in R. Journal of Statistical Software, 45(3), 1-67. DOI 10.18637/jss.v045.i03.

Listing of Tables, Listings and Figures

An appendix document gives precise details for each table, listing or figure to be produced. As a minimum it will be a tabulation of the following aspects unique to each table or listing.

- Title
- Footnotes
- Numbering
- Population
- Endpoint(s)
- Time Points or details of how to conglomerate multiple observations
- Covariates or subgroups used to break down summary statistics
- Which summary statistics will be calculated
- Or, what formal analysis will be used

Further details to aid the writing of code or improve the report, may be included as well, for example a list of variable names from the database; section numbers or titles, subtitles; comments;
